# Environmental stress perception activates structural remodeling of extant *Streptococcus mutans* biofilms

**DOI:** 10.1038/s41522-020-0128-z

**Published:** 2020-03-27

**Authors:** Patrick Marx, Yu Sang, Hua Qin, Qingjing Wang, Rongkai Guo, Carmem Pfeifer, Jens Kreth, Justin Merritt

**Affiliations:** 1 Department of Restorative Dentistry, School of Dentistry, Oregon Health & Science University, Portland, OR 97239 USA; 2 Department of Molecular Microbiology and Immunology, Oregon Health & Science University, Portland, OR 97239 USA

**Keywords:** Microbial genetics, Biofilms

## Abstract

Transcription regulators from the LexA-like Protein Superfamily control a highly diverse assortment of genetic pathways in response to environmental stress. All characterized members of this family modulate their functionality and stability via a strict coordination with the coprotease function of RecA. Using the LexA-like protein IrvR from *Streptococcus mutans*, we demonstrate an exception to the RecA paradigm and illustrate how this evolutionary innovation has been coopted to diversify the stress responsiveness of *S. mutans* biofilms. Using a combination of genetics and biophysical measurements, we demonstrate how non-SOS stresses and SOS stresses each trigger separate regulatory mechanisms that stimulate production of a surface lectin responsible for remodeling the viscoelastic properties of extant biofilms during episodes of environmental stress. These studies demonstrate how changes in the external environment or even anti-biofilm therapeutic agents can activate biofilm-specific adaptive mechanisms responsible for bolstering the integrity of established biofilm communities. Such changes in biofilm community structure are likely to play central roles in the notorious recalcitrance of biofilm infections.

## Introduction

The regulatory interplay between RecA and LexA during the bacterial SOS response is among the most thoroughly characterized prokaryotic stress response mechanisms. In the presence of various genotoxic stressors, such as the antibiotic mitomycin C, stalled DNA replication forks and/or various forms of DNA damage create single-stranded DNA (ssDNA) segments that activate the RecA recombinase into a Mg^2+^/ATP-binding state referred to as RecA*. Once activated, RecA* is able to assemble into nucleoprotein filaments at ssDNA segments^1–3^. RecA* also serves as the coprotease for the SOS response repressor LexA by stimulating endogenous LexA peptidase activity to trigger its autocleavage and subsequent proteolytic degradation^1,4–6^. Following LexA autocleavage, the SOS response genes under LexA control become derepressed, resulting in the synthesis of proteins designed to mitigate the deleterious effects of DNA damage^1,4,5,7^.

LexA is the archetypal member of the eponymous LexA-like Protein Superfamily^5^ (also referred to as Peptidase S24-like Superfamily). All LexA-like proteins contain a C-terminal S24 peptidase domain, which is a serine peptidase catalyzed by a highly conserved serine–lysine dyad within the enzyme catalytic site^8,9^. Members of this family autocleave with high specificity between strictly conserved adjacent alanine and glycine residues^8,9^. Upon autocleavage, embedded C- and N-degrons become terminally exposed and target the autocleavage fragments for processive exoproteolysis via housekeeping proteases such as Clp, Lon, and FtsH^10–15^. Most members of this family are transcription repressors and encode N-terminal DNA binding domains. Proteins such as LexA and lysogenic bacteriophage CI repressors are classic examples^4,5^. Like LexA, members of this protein family also depend upon the coprotease function of RecA* to stimulate their inherent autocleavage abilities^4–6,16,17^. Accordingly, uncharacterized putative LexA-like proteins are often annotated as “RecA-mediated autopeptidases”.

While LexA-like proteins are renowned for their role in the SOS response, there are numerous examples of these proteins controlling other genetic pathways in response to environmental stress^5^. For example, in *Pseudomonas aeruginosa*, the LexA-like protein PrtR participates in the response to genotoxic stress by modulating pyocin synthesis and the expression of type III secretion system genes, rather than components of the SOS response^18,19^. Similar to other LexA-like proteins, the control of PrtR derepression is a RecA-dependent process^19^, which presumably provides a simple mechanism for the cell to coordinate the expression of the PrtR regulon with the perception of environmental stress. This is highly analogous the CI-Cro paradigm controlling the lytic reactivation of lysogenic bacteriophages^20^. Previously, we characterized a similar SOS-independent LexA-like transcription regulator (IrvR) from the human dental caries pathogen *Streptococcus mutans*. As is typical for members of this family, IrvR has a C-terminal S24 peptidase domain and autocleaves between conserved alanine and glycine residues^14^. Following IrvR autocleavage, the N-terminal autocleavage fragment (NTD) containing the IrvR DNA binding domain is targeted for ClpXP proteolysis due to the exposure of a terminal C-degron^14^. Unlike the *E. coli* LexA protein, the IrvR NTD retains its normal transcription repressor function following autocleavage provided its C-degron is mutated or deleted, which is similar to the LexA-like protein HdiR from *Lactococcus lactis*^14,15,21^. Thus, the proper functioning of the C-degron is also critical for the derepression of IrvR-regulated genes.

The primary target of IrvR repression is the gene *irvA*, which is located directly adjacent to *irvR* on the chromosome^22^. While *irvA* gene expression is normally maintained at a low basal level by IrvR, exposure to environmental stress can derepress *irvA* gene expression leading to a variety of phenotypes in various accessory gene pathways controlling bacteriocin production, natural competence development, and the dextran-dependent aggregation (DDAG) stress response^22–25^. Recently, we determined the mechanistic connection between *irvA* gene expression and the DDAG response. This pathway is triggered by environmental stress and has a characteristic phenotype of large cellular aggregates forming in liquid cultures supplemented with dextran polymers^26–28^. The source of the DDAG clumping phenotype is due to stress-induced production of a cell wall anchored dextran-binding surface lectin called glucan-binding protein C (GbpC)^23,26^. Intriguingly, GbpC produced in response to xylitol stress is critically dependent upon a posttranscriptional stabilization of transcribed *gbpC* via seed pairing interactions between *irvA* and *gbpC* mRNAs^23^. The net result of this interaction is a substantial increase in *gbpC* mRNA stability and a concomitant increase in GbpC protein abundance, thus triggering the DDAG phenotype. Presently, it is unclear how environmental stress relieves IrvR repression of *irvA*, while the functional role of GbpC during environmental stress has remained enigmatic for nearly two decades. In the current study, we were interested to address these critical knowledge gaps, and in the process, we uncovered a new regulatory paradigm within the LexA-like Protein Superfamily that is essential for controlling a GbpC-dependent remodeling of extant *S. mutans* biofilms in response to environmental stress.

## Results

### IrvR is an exception to the RecA regulatory paradigm for LexA-like proteins

**Fig. 1:**
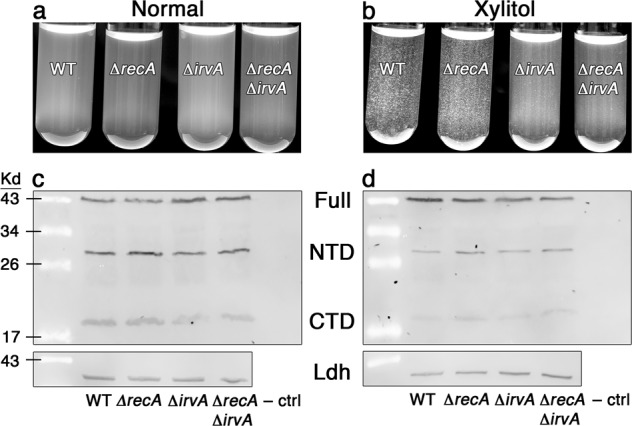
IrvR is RecA-independent and autocleaves constitutively. **a** DDAG assay of the *ldh-irvR* strain and its derivatives performed in the absence of environmental stress. The strains from left to right are: parent *ldh-irvR* (WT), *recA* deletion mutant (Δ*recA*), *irvA* deletion mutant (Δ*irvA*), and *recA/irvA* double deletion mutant (Δ*recA* Δ*irvA*). **b** The same strains were assayed in the presence of xylitol stress. **c** Western blot analysis of the *ldh-irvR* strain and its derivatives performed in the absence of environmental stress. The *ldh-irvR* strain was engineered to express both a C-terminal FLAG tag as well as an internal FLAG tag, which supports the detection of the full-length protein (Full), N-terminal autocleavage fragment (NTD), and C-terminal autocleavage fragment (CTD). The strains from left to right are: parent *ldh-irvR* (WT), *recA* deletion mutant (Δ*recA*), *irvA* deletion mutant (Δ*irvA*), *recA/irvA* double deletion mutant (Δ*recA* Δ*irvA*), and *ldh-irvR* strain lacking the FLAG epitope (–ctrl). The bottom panel is an HA-tagged lactate dehydrogenase loading control. **d** The same strains were assayed in the presence of xylitol stress. See also Supplementary Fig. 1.

**Fig. 2:**
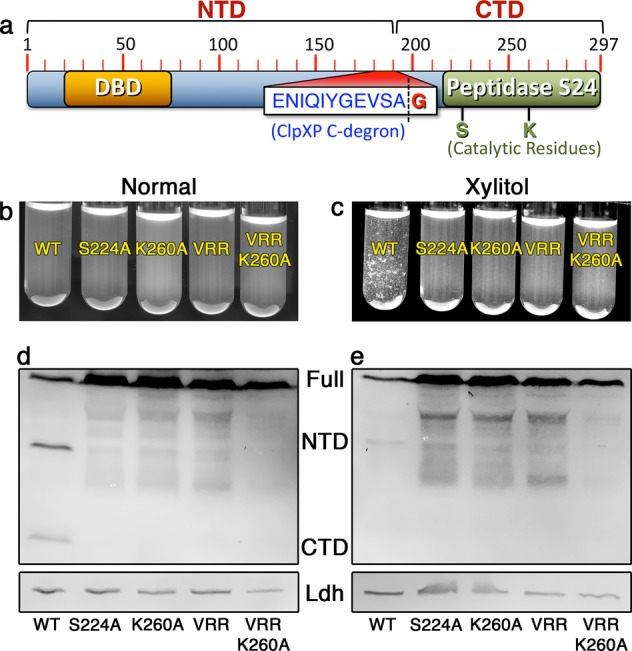
IrvR has the characteristic features of a LexA-like protein. **a** Schematic map of the IrvR protein. The numbered red hash marks indicate the corresponding amino acid positions in the protein. The sequence of the ClpXP C-degron is written in blue font and the position of the autocleavage site is indicated by an adjacent dashed black line. The location of the DNA binding domain (DBD) is indicated in orange, while the peptidase S24 domain is shown in green with the positions of its catalytic residues indicated underneath. **b** DDAG assay of the *ldh-irvR* strain and its derivatives performed in the absence of environmental stress. The strains from left to right are: parent *ldh-irvR* (WT), S224A mutant IrvR (S224A), K260A mutant IrvR (K260A), C-degron/autocleavage site mutant IrvR (VRR), and C-degron/autocleavage site + K260A double mutant IrvR (VRR K260A). **c** The same strains were assayed in the presence of xylitol stress. **d** Western blot analysis of the *ldh-irvR* strain and its derivatives performed in the absence of environmental stress. The strains from left to right are: parent *ldh-irvR* (WT), S224A mutant IrvR (S224A), K260A mutant IrvR (K260A), C-degron/autocleavage site mutant IrvR (VRR), and C-degron/autocleavage site + K260A double mutant IrvR (VRR K260A). The bottom panel is an HA-tagged lactate dehydrogenase loading control. **e** The same strains were assayed in the presence of xylitol stress. See also Supplementary Fig. 2.

In our previous studies, we identified a number of IrvR features that are typical of LexA-like proteins, such as a putative C-terminal S24 peptidase domain, a conserved alanine/glycine autocleavage site, and an embedded ClpXP C-degron^14^. Given that IrvR is the primary repressor of *irvA* gene expression^14,22^, we hypothesized that the derepression of *irvA* previously observed during xylitol stress^23^ was likely due to the activation of the coprotease function of RecA* and a resulting stimulation of IrvR autocleavage. To test this, we examined whether IrvR autocleavage fragments accumulate during exposure to xylitol stress. However, IrvR was found to be exceptionally unstable, as it was difficult to detect via western blots, despite its highly robust reporter signal when expressed as a luciferase fusion protein (Supplementary Fig. 1a, b). Therefore, we transcriptionally fused *irvR* to the highly expressed *S. mutans* lactate dehydrogenase (*ldh*) promoter to simplify its detection (Supplementary Fig. 1a). Next, we confirmed that the *ldh-irvR* strain yields an identical DDAG response as the wild-type, indicating that this strain exhibits normal IrvR function despite its increased expression (Fig. 1a, b, Supplementary Fig. 1c, d). We were surprised to discover that in both the wild-type and *ldh-irvR* backgrounds, a *recA* deletion mutation had no impact upon the DDAG phenotype as hypothesized. This is in stark contrast to an *irvA* mutation, which potently suppressed the DDAG phenotype (Fig. 1a, b, Supplementary Fig. 1c, d). Even more unexpectedly, we observed a constitutive autocleavage of IrvR that occurred irrespective of xylitol stress and RecA (Fig. 1c, d, and Supplementary Fig. 1a). The only observable stress-dependent effect upon IrvR was an apparent reduction in IrvR abundance, as longer western blot exposure times were required to yield comparable images to the non-stressed samples (Fig. 1c, d). Based upon these results, we questioned whether IrvR is truly a LexA-like protein. As shown in Fig. 2a, IrvR has the expected features of a classic LexA-like protein, including a characteristic S24 peptidase domain, which we predicted to utilize serine 224 and lysine 260 as its catalytic residues due to sequence alignments with known LexA-like proteins. Consistent with this prediction, these catalytic residues and the IrvR C-degron/autocleavage site were all essential for the stress-induced DDAG response as well as IrvR autocleavage in both the wild-type and *ldh-irvR* strains (Fig. 2b–e and Supplementary Fig. 2a–c). Likewise, all of the autocleavage mutant strains similarly exhibited an accumulation of the full-length form of IrvR as a result of autocleavage deficiencies (Fig. 2d, e, and Supplementary Fig. 2a). From these results, we conclude that IrvR is indeed a LexA-like protein, despite its RecA-independent autocleavage ability.

### IrvR has a homodimerization domain within the NTD that mediates its autocleavage

**Fig. 3:**
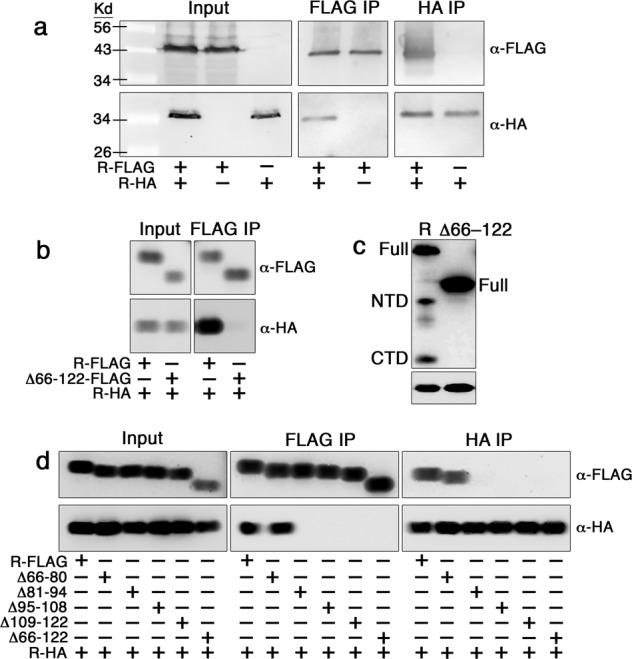
IrvR contains an internal homodimerization domain. **a** *S. mutans* was engineered to express two copies of *irvR*, one encoding an N-terminal FLAG tag (R-FLAG) and the other encoding an N-terminal HA tag (R-HA). Homodimerization between the FLAG and HA tagged IrvR proteins was assessed via coimmunoprecipitation. Figure columns labeled FLAG IP and HA IP indicate the antibody-conjugated resins used for immunopurification, while the rows labeled α-FLAG and α-HA indicate the antibodies used for western blot detection. **b** N-terminal epitope tagged IrvR proteins were assessed for homodimerization via coimmunoprecipitation. The full-length FLAG tagged IrvR (R-FLAG) and Δ66–122 internal deletion FLAG tagged IrvR (Δ66–122-FLAG) were compared for their ability to dimerize with the full-length HA tagged IrvR (R-HA). **c** Western blot was used to compare the autocleavage abilities of the parent *ldh-irvR* strain (R) and its Δ66–122 internal deletion mutant (Δ66–122) derivative. The bottom panel is a lactate dehydrogenase loading control. **d** The *ldh-irvR* strain encoding an N-terminally FLAG tagged full-length IrvR as well as its internal deletion mutant derivatives were tested via coimmunoprecipitation for their homodimerization abilities with a full-length N-terminally HA tagged IrvR. Strains from top to bottom are: full-length FLAG tagged IrvR (R-FLAG), Δ66–80 internal deletion FLAG tagged IrvR (Δ66–80), Δ81–94 internal deletion FLAG tagged IrvR (Δ81–94), Δ95–108 internal deletion FLAG tagged IrvR (Δ95–108), Δ109–122 internal deletion FLAG tagged IrvR (Δ109–122), Δ66–122 internal deletion FLAG tagged IrvR (Δ66–122), and full-length HA tagged IrvR (R-HA). See also Supplementary Fig. 3.

Given the dispensable role of RecA for IrvR autocleavage, we were curious whether IrvR might interact with another protein capable of serving a similar coprotease function. However, after repeated IrvR pull-down screening experiments, we failed to identify any convincing protein interactions with IrvR other than its self-interactions (i.e., homodimerization). While the bacteriophage λ CI repressor primarily autocleaves as a monomer^29,30^, both LexA and the 434 CI repressor autocleave while bound to DNA as a dimer^17,31^. Therefore, we were curious whether IrvR might directly trigger its own autocleavage via dimerization and/or DNA binding. As shown in Supplementary Fig. 3a, b, the repressor function of IrvR is almost entirely abrogated by appending an N-terminal FLAG epitope onto the protein, indicating a severe DNA-binding defect. Despite this, the N-terminal FLAG tagged IrvR as well as an IrvR Δ1–65 N-terminal truncation mutant were both fully proficient in IrvR dimerization and autocleavage (Fig. 3a, Supplementary Fig. 3c, d), which indicates that both processes occur independent of DNA binding. Using coimmunoprecipitation, we assayed a variety of IrvR truncation fragments and determined that IrvR likely contains a homodimerization domain in the NTD between amino acids 66–122 (Supplementary Fig. 3e), which is a region of the protein devoid of other predicted functional domains (Fig. 2a). As an independent confirmation, we deleted residues 66–122 from the full length IrvR and similarly observed a total loss of dimerization and autocleavage abilities (Fig. 3b, c). Using successive 15 amino acid deletions, we further determined that residues 81–122 are essential for IrvR dimerization, whereas residues 66–80 are dispensable (Fig. 3d). Thus, IrvR contains an internal interaction site within the NTD that mediates its autocleavage and minimally consists of residues 81–122.

### IrvR evolution away from RecA-dependence diversifies DDAG responsiveness

**Fig. 4:**
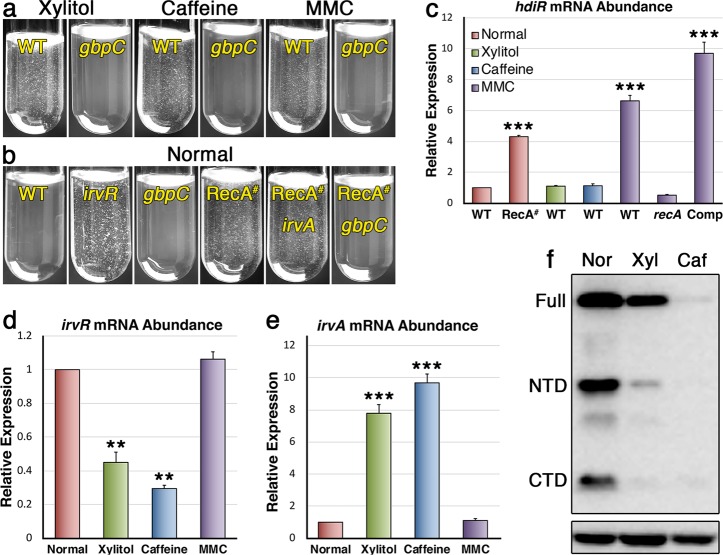
GbpC production is controlled by two parallel stress-dependent pathways. **a** The DDAG phenotypes of the *ldh-irvR* strain (WT) and its Δ*gbpC* derivative (*gbpC*) were compared in the presence of xylitol stress, caffeine stress, and mitomycin C (MMC) stress. **b** The DDAG phenotypes of the *ldh-irvR* strain and its mutant derivatives were compared in the absence of added stress. Strains from left to right are: *ldh-irvR* parent strain (WT), Δ*irvR* deletion mutant (*irvR*), Δ*gbpC* deletion mutant (*gbpC*), P80D/D171R mutant RecA (RecA^#^), P80D/D171R mutant RecA + Δ*irvA* deletion mutation (RecA^#^ *irvA*), and P80D/D171R mutant RecA + Δ*gbpC* deletion mutation (RecA^#^ *gbpC*). **c** The expression of the LexA paralog *hdiR* was assessed via qRT-PCR in both normal and stress growth conditions. Expression values (±s.d.) and statistical comparisons are presented relative to the *ldh-irvR* parent strain cultured in normal growth conditions, which was arbitrarily assigned an expression value of 1. Bars colored in red indicate samples cultured in normal conditions, whereas green indicates xylitol stress, blue indicates caffeine stress, and purple indicates mitomycin C (MMC) stress. Strains from left to right are: *ldh-irvR* parent strain (WT), P80D/D171R mutant RecA (RecA^#^), *ldh-irvR* parent strain (WT), *ldh-irvR* parent strain (WT), *ldh-irvR* parent strain (WT), Δ*recA* deletion mutant (*recA*), and complemented Δ*recA* deletion mutant (Comp). **d** The *ldh-irvR* strain was cultured in normal and stress growth conditions and then assayed for *irvR* transcript abundance via qRT-PCR. Expression values (±s.d.) and statistical comparisons are presented relative to the *ldh-irvR* parent strain cultured in normal growth conditions, which was arbitrarily assigned an expression value of 1. **e** The *ldh-irvR* strain was cultured in normal and stress growth conditions and then assayed for *irvA* transcript abundance via qRT-PCR. Expression values (±s.d.) and statistical comparisons are presented relative to the *ldh-irvR* parent strain cultured in normal growth conditions, which was arbitrarily assigned an expression value of 1. **f** The *ldh-irvR* strain was assayed via western blot to compare IrvR abundance in normal and stress growth conditions. The bottom panel is a lactate dehydrogenase loading control. Error bars represent the standard deviations from at least three independent experiments. ***P < 0.001 and **P < 0.01, Unpaired two-tailed Student’s *t* test. See also Supplementary Fig. 4.

Our results indicated that IrvR possesses all of the highly conserved features found in typical LexA-like proteins, except for a dependence upon RecA* coprotease activity. Thus, it appeared as if the ancestral IrvR protein began as a typical RecA-dependent LexA-like regulator and then evolved a unique ability to independently mediate its own autocleavage. We were curious whether the evolution of this unusual autocleavage mechanism might be due to some selective advantage afforded by RecA-independence. Previous studies have reported a variety of stress conditions capable of triggering the DDAG response in *S. mutans* and other related species^28,32,33^. Therefore, we suspected that IrvR could have evolved to respond to non-SOS (i.e., RecA-independent) stresses. To test this, we compared the DDAG phenotypes of a non-cleavable IrvR mutant and a Δ*recA* mutant in response to a variety of different classes of stress (Table 1). Similar to the xylitol DDAG results, caffeine, ethanol, and puromycin all triggered DDAG via IrvR and independent of RecA. Unexpectedly, mitomycin C, the classic inducer of the SOS response, as well ammonium sulfate and sodium fluoride were also capable of inducing DDAG, but in an IrvR-independent and RecA-dependent manner (Table 1). As further confirmation, we repeated the DDAG assays using both the parent *ldh-irvR* strain and its *gbpC* mutant derivative in the presence of the IrvR-dependent stressors xylitol and caffeine as well as the RecA-dependent stressor mitomycin C. Each of these stresses was similarly incapable of triggering DDAG from the *gbpC* mutant strain (Fig. 4a), indicating that there are at least two distinct stress-regulated pathways to activate GbpC production (i.e., the DDAG response). We next asked whether the newly identified RecA-dependent DDAG pathway was truly associated with the SOS response, as would be expected of mitomycin C stress (Table 1). A variety of constitutive RecA*-activating mutations have been previously identified in *E. coli*^34–37^. Therefore, we identified the equivalent residues for mutagenesis in the *S. mutans* RecA (Supplementary Fig. 4a). After testing a variety of *recA* point mutations, we found that a double point mutant *recA* encoding both P80A and D171R substitutions was able to trigger a constitutive DDAG^+^ phenotype (Fig. 4b and Supplementary Fig. 4b–f). This phenotype could not be suppressed by an *irvA* mutation, whereas the P80D/D171R mutant RecA is hypostatic to *gbpC*, the final step in the DDAG genetic pathway (Fig. 4b). To determine whether the P80D/D171R RecA mutations also activate the SOS response, we measured the expression of *hdiR*, which is the SOS stress-inducible (i.e., RecA-dependent) LexA paralog encoded by streptococci and other closely related species^12,15,38,39^. As predicted, the P80D/D171R RecA strain exhibited both constitutive DDAG and *hdiR* derepression, even in the absence of added stress (Fig. 4b, c). Likewise, the SOS stressor mitomycin C derepressed *hdiR* in a *recA*-dependent manner, whereas the IrvR-dependent stressors xylitol and caffeine completely lacked this ability (Fig. 4c). To determine how stress could inhibit IrvR function in the absence of RecA control, we assayed *irvR* and *irvA* gene expression in normal vs. stress growth conditions and found that unlike mitomycin C, both xylitol and caffeine inhibit *irvR* gene expression and stimulate *irvA* production (Fig. 4d, e). Likewise, xylitol and caffeine also strongly reduce IrvR protein abundance with caffeine triggering a more robust effect (Fig. 4f), which is consistent with the greater inhibition of *irvR* gene expression and stronger DDAG phenotypes generated by caffeine (Table 1 and Fig. 4d). We found no obvious stress-induced changes in IrvR protein half-life (Supplementary Fig. 4g). Therefore, the primary effects of both xylitol and caffeine likely occur at the *irvR* transcriptional and/or posttranscriptional levels. Overall, our results indicate that the intriguing ability of *S. mutans* to activate the DDAG response in the presence of such a wide assortment of stresses is primarily due to it having both a non-SOS stress-regulated pathway for GbpC production (i.e., IrvR-dependent) as well as an SOS stress-regulated pathway for GbpC production (i.e., RecA-dependent).

**Table 1:** DDAG phenotypes from different classes of environmental stress.

Stress	WT^*^	IrvR^VRR*^	recA^*^
None	−	−	−
Ammonium sulfate	+++	+++	−
Caffeine	++++	−	+++
Ethanol	+++	−	+++
Mitomycin C	++	++	−
Puromycin	+++	+	+++
Sodium fluoride	+++	+++	−
Xylitol	+++	−	+++

^*^++++ indicates strong DDAG in ≤10 s.

^*^+++ indicates strong DDAG in 11–30 s.

^*^++ indicates moderate DDAG in 31–60 s.

^*^+ indicates weak DDAG in 61–120 s.

### GbpC modulates the biophysical properties of extant *S. mutans* biofilms

**Fig. 5:**
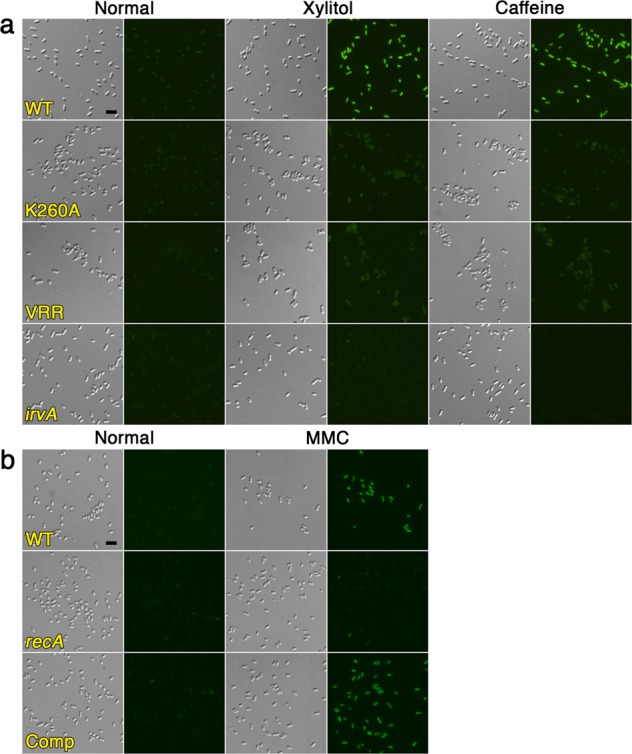
IrvR- and RecA-dependent stresses trigger *gbpC* expression within flow cell biofilms. **a** An *S. mutans gbpC-gfp* transcription fusion reporter strain and its derivatives were cultured in a drip flow biofilm reactor for 16 h to develop biofilms. The biofilms were subsequently cultured for an additional 4 h ± the indicated environmental stresses before dispersing the cells via sonication and then imaging via differential interference contrast microscopy and epifluorescence microscopy. Fluorescent images were captured with identical camera settings and a 300 ms. exposure time. Strains from top to bottom are: parent *gbpC-gfp* reporter strain (WT), IrvR K260A mutant (K260A), C-degron/autocleavage site mutant IrvR (VRR), and Δ*irvA* deletion mutant (*irvA*). **b** An identical experimental set up was used to assess the effect of mitomycin C (MMC) stress. Strains from top to bottom are: parent *gbpC-gfp* reporter strain (WT), Δ*recA* deletion mutant (*recA*), and complemented Δ*recA* deletion mutant (Comp). Scalebars indicate 1 µm. See also Supplementary Fig. 5.

**Fig. 6:**
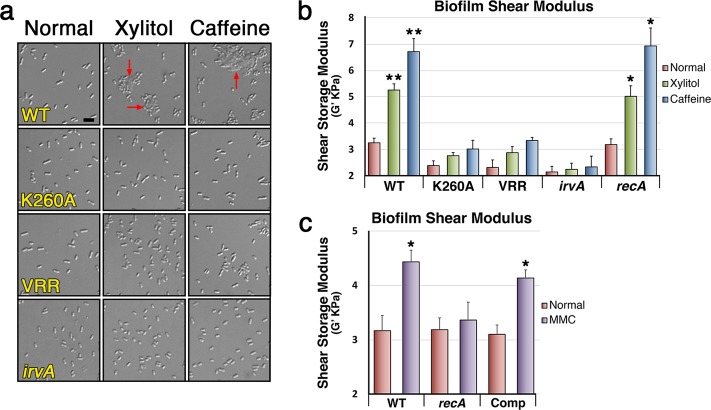
Environmental stress alters the biophysical properties of extant biofilms. **a** The *ldh-irvR* strain and its mutant derivatives were cultured on hydroxyapatite (HA) disks for 16 h in a drip flow biofilm reactor to develop biofilms. The biofilms were subsequently cultured for an additional 4 h ± the indicated environmental stresses before dispersing the cells using identical sonication conditions and then imaging via differential interference contrast microscopy. Red arrows indicate biofilm fragments resistant to sonication. The strains from top to bottom are *ldh-irvR* parent strain (WT), IrvR K260A mutant (K260A), C-degron/autocleavage site mutant IrvR (VRR), and Δ*irvA* deletion mutant (*irvA*). Scalebar indicates 1 µm. **b** The *ldh-irvR* parent strain and its mutant derivatives were cultured in static growth conditions for 16 h to develop biofilms directly onto detachable rheometer plates. The biofilms were subsequently cultured for an additional 4 h ± the indicated environmental stresses before attaching the biofilm-coated rheometer plates onto a rheometer and then measuring the storage modulus of the resulting biofilms. Results are presented as the average shear storage modulus values ± s.d. Strains from left to right are: *ldh-irvR* parent strain (WT), K260A IrvR mutant (K260A), C-degron/autocleavage site mutant IrvR (VRR), Δ*irvA* deletion mutant (*irvA*), and Δ*recA* deletion mutant (*recA*). **c** An identical experimental set up was used to assess the effect of mitomycin C (MMC) stress. Strains from left to right are: *ldh-irvR* parent strain (WT), Δ*recA* deletion mutant (*recA*), and complemented Δ*recA* deletion mutant (Comp). All statistical comparisons were performed relative to the parent *ldh-irvR* strain cultured in normal growth conditions. **P < 0.01 and *P < 0.05, Unpaired two-tailed Student’s *t* test. See also Supplementary Fig. 6.

As a dextran/glucan-binding surface lectin, basal levels of GbpC are critical for the proper development of *S. mutans* biofilms due to its lectin interactions with the abundant dextran/glucan polymers present within the biofilm matrix^40^. However, it has remained a longstanding mystery why *S. mutans* requires increased GbpC production during environmental stress. Since the vast majority of *S. mutans* in the oral cavity is located within biofilms, we suspected that the characteristic planktonic aggregation of the DDAG response may have minimal biological relevance, rather it may in fact be indicative of another process that would normally occur within biofilms. Consequently, we were interested to determine whether environmental stress similarly induces GbpC production within biofilms and to what extent, if any, this influences biofilm structure. Using a *gbpC-gfp* reporter strain, we first grew biofilms on standard 5 mm diameter hydroxyapatite (HA) disks to provide biologically relevant substrates for biofilm development in both static and flow cell conditions^41^. Afterward, we exposed the preformed biofilms to xylitol, caffeine, and mitomycin C. For the flow cell experiments, custom 3-D printed flow cell inserts were used to house the HA disks (Supplementary Fig. 5a). Like planktonic cultures, both static and flow cell biofilms responded similarly to all three stresses by activating *gbpC* expression (Fig. 5a, b, Supplementary Fig. 5b, c). For xylitol and caffeine stress, this effect was strongly abrogated in the non-cleavable IrvR and Δ*irvA* backgrounds (Fig. 5a, b, Supplementary Fig. 5b, c). Likewise, mitomycin C failed to induce GFP fluorescence in the *recA* background, but did induce fluorescence in the wild-type and complemented *recA* mutant. During the process of disrupting the biofilms to examine single cell GFP fluorescence, we also noticed that it required more aggressive sonication conditions to fully disperse the GFP-expressing biofilms. To independently confirm this observation, we repeated the biofilm stress experiments by exposing preformed biofilms to xylitol and caffeine stress and then simultaneously subjecting all of the biofilms to identical sonication conditions in a single waterbath sonicator using power and time settings previously determined to completely disperse untreated wild-type biofilms formed on HA disks. Next, the supernatants from each of the sonicated biofilms were examined by microscopy to compare the degree of biofilm disruption in each sample. As shown in Fig. 6a and Supplementary Fig. 6, supernatants from the untreated wild-type biofilms only contained single and double cells as expected, indicating a complete dispersion of the biofilms formed on HA disks. Identical results were also observed from the stress-treated non-cleavable IrvR and Δ*irvA* mutant biofilms. In contrast, exposure to environmental stress transformed only the wild-type static and flow cell biofilms into a sonication resistant state, as large aggregates of undisrupted biofilm cells were still visible, indicating an incomplete dispersion of these biofilms. To further quantify this effect, we used a rheometer to measure the shear moduli (G′) of preformed biofilms following exposure to environmental stress. In agreement with the DDAG and *irvR/A* expression phenotypes, both xylitol and caffeine triggered significant increases in the shear storage modulus of wild-type biofilms with caffeine exerting the stronger effect (Table 1, Figs. 4d–f and 6b). In contrast, the non-cleavable IrvR and Δ*irvA* mutant biofilms were largely unresponsive to stress treatments, whereas the Δ*recA* mutant biofilms yielded a wild-type increase in shear storage modulus (Fig. 6b). An analogous RecA-dependent change in biofilm shear modulus was obtained with mitomycin C treatment as well, albeit to a lesser extent compared to xylitol and caffeine, which is consistent with its similarly weaker *gbpC* expression and DDAG phenotypes (Table 1, Fig. 5b, Supplementary Figs. 5c and 6c). Given the dynamic biophysical properties of *S. mutans* biofilms, we conclude that stress-induced GbpC production serves to create a highly crosslinked network of lectin interactions between cells and the glucan polymers within the biofilm matrix to bolster structural integrity during adverse growth conditions. Since this change can occur even in extant biofilms, it demonstrates a mechanism whereby *S. mutans* is able to structurally remodel its biofilms in situ in response to hostile changes in the external environment.

## Discussion

**Fig. 7:**
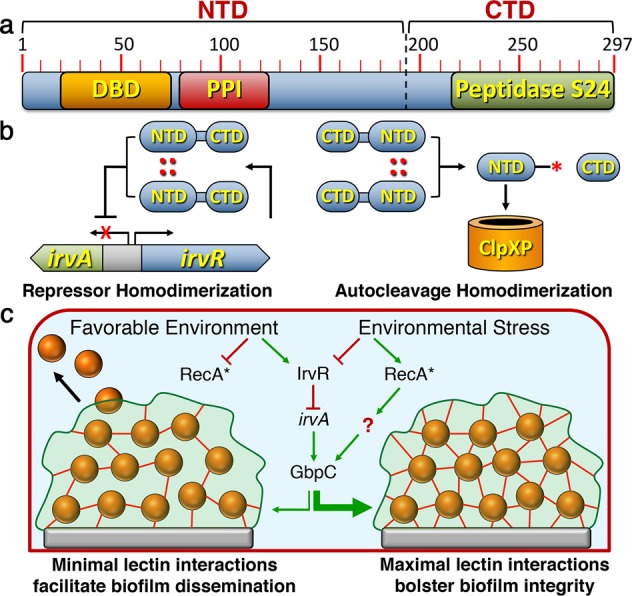
Model of IrvR interactions and stress-induced biofilm remodeling. **a** Schematic map of IrvR. The protein-protein interaction (PPI) domain required for IrvR homodimerization is shown in red, while the autocleavage site is indicated by a dashed black line. **b** NTD dimerization facilitates DNA binding and the repression of *irvA* gene expression. In addition, NTD dimerization is essential for autocleavage, presumably supporting a trans cleavage reaction of one or both IrvR monomers. Autocleavage exposes a terminal C-degron (represented by a red asterisk), which targets the IrvR NTD for ClpXP proteolysis. **c** Illustration of the two parallel pathways controlling GbpC production during environmental stress. In a favorable growth environment, IrvR represses *irvA* gene expression, whereas RecA exists in its basal nonactivated state. Due to the unusually short half-life of *gbpC* mRNA, only a modest amount of cell wall anchored GbpC is constitutively produced (represented by red lines extending from the cells). This creates a biofilm with a minimal network of GbpC lectin interactions with the glucan polymers present in the biofilm matrix. The resulting biofilm exhibits a flexible architecture that is readily dispersible. Under conditions of environmental stress, GbpC production is stimulated in situ within biofilms. Depending upon the source of stress, either IrvR production could be inhibited to trigger the IrvR-dependent pathway or RecA could be activated to trigger the RecA-dependent pathway. For the IrvR-dependent pathway, the derepression of *irvA* results in a substantial stabilization of *gbpC* mRNA and a concomitant increase in GbpC production. As GbpC concentration increases, biofilm mechanical properties are modified due to the formation of a highly rigid structure supported by an increasingly complex network of GbpC lectin interactions with glucan polymers in the biofilm matrix. Biofilms exposed to environmental stress become progressively more difficult to disperse proportionally with GbpC abundance. Since GbpC production is also proportional to the severity of environmental stress, the internal structure of *S. mutans* biofilms is able to maintain synchronization with the current environmental conditions.

One of the key regulatory features of the IrvR-dependent pathway is a unique constitutive autocleavage ability encoded within IrvR (Fig. 1c, d). IrvR autocleavage is critically dependent upon a homodimerization domain located within the NTD (Figs. 3a–d and 7a). In contrast, the model LexA-like proteins LexA and λ CI have their primary dimer interfaces located within the CTD^29,42^. This distinct mode of IrvR dimerization may also explain why IrvR and possibly HdiR from *L. lactis* both retain their normal transcription repressor abilities after their autocleavage^14,15^. Since the IrvR dimer interface is located within the NTD, autocleavage should not impair dimerization of the IrvR DNA binding domain (Fig. 3b, d, Supplementary Figs. 3e and 7a, b), which contrasts with both LexA and λ CI, where NTD dimerization is too weak to support their repressor activities following autocleavage^29,42^. In addition to the IrvR-dependent pathway, we also discovered a separate RecA-dependent pathway that similarly responds to environmental stress by increasing GbpC production (Figs. 4a, b, 5b, and 7c). The regulatory mechanism employed by this pathway remains to be determined, but several lines of evidence indicate that it is active concurrent with the SOS response: (1) the pathway is mitomycin C-inducible (Table 1, Figs. 4a and 5b), (2) the constitutively activated P80D/D171R RecA mutant exhibits a constitutive DDAG^+^ phenotype (Fig. 4b), and (3) both mitomycin C and P80D/D171R RecA mutations derepress expression of the SOS-responsive LexA paralog *hdiR* (Fig. 4c). Based upon the presented results, we propose the following model to describe how *S. mutans* is able to connect environmental stress perception with the structural remodeling of extant biofilms. During favorable growth conditions, IrvR efficiently represses *irvA* gene expression and RecA is maintained in its nonactivated state. This results in a low basal level of GbpC produced on the cell surface, which is sufficient for normal biofilm formation^40^. These biofilms exhibit low structural rigidity and are readily dispersible (Fig. 6a–c), which we propose as facilitating the biological imperative for biofilm dissemination (Fig. 7c). A favorable growth environment would also signal the opportune conditions to establish new communities. In contrast, during episodes of severe environmental stress, GbpC production is strongly activated within biofilms (Fig. 5a, b, Supplementary Fig. 5b, c), resulting in a remodeling of biofilms into a highly resilient rigid structure that resists dispersion (Fig. 6a–c). In a hostile growth environment, we speculate that the community’s basic survival needs would supersede the imperative for dispersion and dissemination, particularly if the current environment is detrimental for the establishment of new biofilm communities. Furthermore, by placing these two parallel GbpC pathways under separate IrvR and RecA control, *S. mutans* is able to trigger GbpC production by a vast array of different classes of stress. This provides a mechanistic explanation for the unusually broad diversity of stressors capable of triggering the DDAG response (Table 1)^27,28^.

From an evolutionary perspective, the stress-dependent regulation of GbpC is apparently critical for *S. mutans* persistence, as it has evolved at least two independent stress-regulated pathways to do so (Fig. 7c). We suspect that the benefits gained by connecting GbpC production to an increasing diversity of stresses may have provided the selective advantage facilitating IrvR evolution away from a dependence upon RecA* coprotease activity. Certainly, we cannot exclude the possibility that the ancestral IrvR protein was similarly RecA-independent, but this seems highly unlikely given the lack of other RecA-independent LexA-like regulators known to exist. It is also worth noting that IrvR and IrvA share extensive sequence homology to multiple streptococcal lysogenic phage CI (37% identical/50% similar to IrvR) and Cro (65% identical/79% similar to IrvA) regulators, despite being components of the core *S. mutans* genome, rather than bona fide lysogenic phage proteins^14,23^. This suggests that the *irvR/A* genes were likely ancient acquisitions via horizontal gene transfer and further implicates degraded phage genomes as potential reservoirs of novel LexA-like regulators. As previously mentioned, bacteriophage CI regulators are classic examples of RecA-dependent LexA-like proteins^4,5^, which is yet another reason to suspect a RecA-dependent ancestral origin for IrvR.

The unique constitutive autocleavage ability of IrvR poses an interesting conundrum: if autocleavage is not regulated, what role might it play in the IrvR-dependent pathway? Despite being constitutive, IrvR autocleavage is indeed essential. Non-cleavable IrvR mutants constitutively repress *irvA* gene expression, lack the ability to engage the DDAG response, and fail to structurally remodel biofilms in response to environmental stress (Figs. 1b, c and 6a, b)^14^. We propose that the autocleavage step plays a key role in maintaining the equilibrium between IrvR synthesis and degradation. IrvR is a highly unstable protein that is difficult to detect via western blot unless its autocleavage ability is disrupted (Supplementary Figs. 1a and 2a). Its high turnover rate suggests that IrvR production requires constant synthesis to maintain an equilibrium favoring *irvA* repression. As a LexA-like protein, IrvR already has an inherent proteolytic degradation mechanism encoded within, due to its embedded C-degron located adjacent to the autocleavage site (Fig. 2a). Accordingly, one might expect that IrvR could have simply evolved a truncation mutation terminating the protein at the autocleavage site to permanently expose the C-degron and achieve a similar destabilization of IrvR. However, the ClpXP proteolysis targeted by this C-degron is likely too efficient to leave permanently exposed, which may explain why it has remained buried within IrvR throughout its evolution. For example, truncating IrvR at the autocleavage site to permanently expose its C-degron results in a complete derepression of *irvA* gene expression mirroring a Δ*irvR* deletion mutation, whereas mutagenesis of the C-degron in the same truncated IrvR mutant renders *irvA* constitutively repressed^14^. By incorporating an autocleavage step into IrvR degradation, this introduces a short time delay before its C-degron is exposed to trigger rapid proteolysis of the IrvR DNA binding domain. As the rate of IrvR synthesis slows due to environmental stress (Fig. 4d), the constitutive degradation of IrvR should quickly outpace its synthesis resulting in a swift change in equilibrium favoring *irvA* derepression. Thus, the constant synthesis and degradation of IrvR provides an alternate strategy to RecA* coprotease regulation for the creation of a sensitive and rapidly responding stress-dependent switch mechanism.

A recent Tn-seq analysis of *S. mutans* identified *irvR* mutants as having >2 orders of magnitude increased fitness in a rodent caries model, whereas *irvA* and *gbpC* mutants both exhibited greatly reduced fitness^43^. Likewise, *S. mutans gbpC* mutants exhibit severely impaired cariogenicity in humans as well as in animal models^44–46^. This underscores the key role of the IrvR-dependent pathway for persistence and virulence in the oral cavity. Our results indicate that one of the principal functions of this pathway is to ensure that *S. mutans* biofilm structural characteristics are appropriately reflective of the current environmental conditions (Fig. 6a–c). In favorable growth conditions, *S. mutans* biofilms are maintained in a state of low structural rigidity, yielding a shear storage modulus of ~3 kPa in our assay system (Fig. 6b, c), which is highly consistent with previous measurements using microindentation^47^. However, we found that the storage modulus of *S. mutans* biofilms is not a fixed property and can actively increase to ~7 kPa if these same biofilms are subsequently exposed to environmental stress (Fig. 6b). This change presumably represents a substantial alteration of biofilm biophysical properties, considering that enzymatic digestion of the exopolysaccharides in wild-type *S. mutans* biofilms results in a similar magnitude change in shear storage modulus^48^. In addition, our results reveal that environmental stress-induced changes in biofilm rheological properties are strongly proportional to GbpC abundance. For example, as shown in Table 1, mitomycin C triggers a weaker DDAG response compared to xylitol, while xylitol is a less potent inducer than caffeine. This same pattern is reflected in the biofilm shear moduli measured for these stresses (i.e., mitomycin C < xylitol < caffeine) (Fig. 6b, c). Similarly, both xylitol and caffeine yield greater *gbpC-gfp* fluorescence in biofilms compared to mitomycin C (Fig. 5a, b, Supplementary Fig. 5b, c). These results also provide an explanation for the enduring mystery regarding the functional role of GbpC during adverse growth conditions. Stress-induced stimulation of GbpC production serves to bolster the mechanical properties of *S. mutans* biofilms, presumably by increasing the number of lectin interactions with the glucan polymers within the biofilm matrix^26,48–50^. Given the similar stress- and GbpC-dependent DDAG phenotypes found in other related bacteria^32,33,51^, we suspect that this role of GbpC is not unique to *S. mutans* biofilms. In fact, there is increasing evidence that other distantly related biofilm forming species also actively modulate the viscoelastic properties of their biofilms, especially as a survival strategy in response to environmental stress^52–54^. In addition, multiple studies in both Gram-positive and Gram-negative bacteria have reported intimate connections between the RecA-dependent SOS response and biofilm development^55–59^. If such pathways were activated in extant biofilms, it is conceivable that they could similarly bolster the viscoelastic properties of those biofilms. Certainly, such a result could also have profound clinical implications for the effective treatment of biofilm associated diseases, as the same therapeutic agents used to treat a biofilm infection could inadvertently impede its clearance and/or promote its persistence^52,54,60–64^.

## Methods

### Bacterial strains, plasmids, and culture conditions

The bacterial strains used/created in this study are listed in Supplementary Table 1. All *S. mutans* strains were grown anaerobically (90% N_2_, 5% CO_2_, and 5% H_2_) at 37 °C. *S. mutans* strains were cultivated in TH broth (Difco) containing 0.3% yeast extract (THYE) and in BTR-G medium (1% Bacto Tryptone, 0.1% bacto Yeast extract, 0.05% Sodium thioglycolate, 0.61% K_2_HPO_4_, 0.2% KH_2_PO_4_, 1 mM MgSO_4_, 0.1 mM MnSO_4_, 0.2% Glucose). THYE medium was used for overnight cultures and for genetic manipulations to create the strains used in this study. For all other experiments, BTR-G medium was used.

### Construction of strains

Specific details of strain construction are described in Supplementary Methods. Strains and plasmids are described in Supplementary Table 1, while primer sequences are listed in Supplementary Table 2. *S. mutans* and its derivatives generated in this study were constructed via overlap extension polymerase chain reaction (OE-PCR). Individual PCR amplicons were generated using Phusion DNA polymerase (Fisher), while OE-PCR reactions were performed with AccuPrime DNA polymerase (Life Technologies). Oligonucleotides were designed using sequence data obtained from the NCBI database (http://www.ncbi.nlm.nih.gov/genome/) and OligoAnalyzer Software 3.1 from Integrated DNA Technologies (https://www.idtdna.com/calc/analyzer). All oligonucleotides were synthesized by Integrated DNA Technologies (Coralville, IA) with a melt temperature adjusted to a minimum of 53 °C following the calculations of OligoAnalyzer Software 3.1. All constructs were integrated onto the *S. mutans* chromosome using homologous recombination via allelic exchange. Both marked and markerless mutations were created in the study. Marked mutations contained the antibiotic resistance cassettes *aphAIII* for kanamycin resistance, *aad9* for spectinomycin resistance, and *ermB* for erythromycin resistance. Transformants were selected on THYE plates containing either 850 µg ml^−1^ kanamycin, 850 µg ml^−1^ spectinomycin, or 12.5 µg ml^−1^ erythromycin. Markerless mutations were created using a two-step integration/excision strategy compatible with OE-PCR amplicons^65^. The counterselectable IFDC2 cassette was first integrated onto the chromosome through allelic exchange and selected with acquired erythromycin resistance. Next, these mutants were transformed with a second construct to remove the IFDC2 cassette via negative selection on THYE plates supplemented with 0.4% (wt/vol) 4-chlorophenylalanine (4-CP).

### Dextran-dependent aggregation (DDAG) assay

Single colonies of *S. mutans* UA159 and its derivatives were used to inoculate THYE broth overnight at 37 °C. The cells were diluted 1:25 into 5 ml fresh BTR-G medium and incubated anaerobically in glass culture tubes at 37 °C until reaching mid-log growth phase. For samples exposed to environmental stress, BTR-G cultures were supplemented with either 2.5% (wt/vol) ammonium sulfate (Fisher), 1% (wt/vol) caffeine (Sigma), 4% (vol/vol) ethanol (Sigma), 25 nM mitomycin C (Sigma), 5 µg ml^−1^ puromycin (Sigma), 0.1 mg ml^−1^ sodium fluoride (Sigma), or 0.6% (wt/vol) xylitol (Sigma). After the incubation period, Dextran T2000 from *Leuconostoc* spp. (Sigma) was added to the cultures at a final concentration of 100 µg ml^−1^ and then the tubes were briefly vortexed at low speed to mix before gently swirling by hand for up to 2 min until obvious DDAG is present in the tubes. The tubes were subsequently placed in front of dark background and illuminated from underneath for imaging.

### Preparation of protein lysates

Overnight THYE cultures of *S. mutans* UA159 and its derivates were diluted 1:25 in 100 ml BTR-G medium ± environmental stress and incubated anaerobically until reaching mid-log growth phase. The cells were harvested by centrifugation (Eppendorf 5810R, 10 min, 3200 *×* *g*, 4 °C), washed twice in phosphate-buffered saline (PBS) buffer (pH 7.4), and then resuspended in 500 µl PBS buffer (pH 7.4) containing 0.2 mM phenylmethylsulfonyl fluoride and 1 mM benzamidine. The suspension was transferred to a 2 ml screw-cap tube containing 1 ml of 0.1 mm silica beads (Biospec) and then lysed in an Omni Bead Ruptor 24 via six 15 s homogenization cycles set at 5 m/s with 1 min incubation periods on ice between cycles. The homogenate was centrifugated for 20 min at 16,000 × *g* in a table top centrifuge at 4 °C and the supernatant was used as the cell lysate for downstream applications. Protein concentrations were determined by Bradford assay using the Protein Assay Dye Reagent (BioRad). Standard curves were created using a bovine serum albumin (Sigma) standard.

### Western blots

A 50 µg of *S. mutans* protein lysate was separated via 14% sodium dodecyl sulfate (SDS) polyacrylamide gel electrophoresis and transferred to a nitrocellulose blotting membrane with 0.45 μm pore size (transfer buffer: 25 mM Tris, 200 mM glycine, and 20% [vol/vol] methanol) for 75 min at 100 V. The membrane was blocked in PBS containing 3% (wt/vol) skim milk (Sigma) for 30 min at room temperature and subsequently incubated with primary antibodies overnight at 4 °C. Monoclonal anti-FLAG M2 primary antibody (Sigma # F3165) was used at a 1:2000 dilution, while the HA Epitope Tag primary antibody (ThermoFisher # 26183) was used at a 1:4000 dilution in PBS with 3% (wt/vol) skim milk. The membrane was washed with PBS, washed twice with tris-buffered saline containing 0.1% (vol/vol) Tween-20 (TBST), followed by a final wash in PBS. Afterward, the membrane was incubated with anti-IgG secondary antibody (Sigma # 12-349) at a 1:2000 dilution in TBST for 1–2 h at room temperature. Washing steps were repeated as described above. For signal detection, the membrane was incubated with Amersham ECL Prime Western Blotting Reagent (GE Healthcare) and imaged using an ImageQuant LAS 4000 (GE Healthcare). All blots presented as part of the same series were derived from the same experiment and were processed in parallel. Original blots are provided in Supplementary Information.

### Coimmunoprecipitation

Overnight cultures of constitutive FLAG-IrvR and/or HA-IrvR expressing *S. mutans* were diluted 1:25 in 500 ml BTR-G medium and incubated anaerobically until reaching mid-log growth phase. Cell lysates were prepared as described above, clarified by centrifugation for 20 min at 16,000 × *g* in a table top centrifuge at 4 °C, and then the protein concentrations in the supernatants were measured via Bradford Assay. Prior to the co-IP binding procedure, 50 µg of protein lysate was removed and saved for western blot analysis as the input samples. Eighty microlitre of anti-FLAG and anti-HA resins (Sigma) were mixed with 1 mg of protein lysate and incubated overnight at 4 °C on a roller shaker. The resin suspension was collected by centrifuging at 5000 × *g* for 1 min and then incubated on ice for 2 min before discarding the supernatant. The resin was washed three times with 1× wash buffer (50 mM Tris-HCl [pH 7.4], 150 mM NaCl) and eluted with 30 µl 2× sample buffer (125 mM Tris-HCl [pH 6.8], 4% (wt/vol) SDS, 20% (vol/vol) glycerol, and 0.004% (vol/vol) bromophenol blue). After a 5 min incubation on a roller shaker at room temperature, the suspension was centrifuged at 5000 × *g* for 1 min. The resulting supernatants were split into two aliquots and used for anti-FLAG and anti-HA immunoblots as the output samples.

### RNA extraction

Overnight cultures of *S. mutans* UA159 and its derivates were diluted 1:25 dilution in 50 ml fresh BTR-G medium ± stress. The cultures were incubated anaerobically until reaching mid-log growth phase, harvested by centrifugation (Eppendorf 5810 R, 10 min, 3200 × *g*, 4 °C), washed twice with 4 °C TE-buffer (10 mM Tris-HCl, 1 mM EDTA, pH 7.8), mixed with 1 ml TRIzol Reagent (Ambion), and then transferred to a 2 ml screwcap tube containing 500 µl 0.1 mm silica beads (Biospec). The suspension was lysed in an Omni Bead Ruptor 24 with two 30 s homogenization cycles at a speed of 6 m/s with a 30 s incubation step on ice between cycles. A 200 µl chloroform (IBI Scientific) was added to the lysates, vortexed for 30 s, and centrifuged in a table top centrifuged at 16,000 × *g* for 15 min at 4 °C. The resulting supernatant was transferred to a 1.7 ml microfuge tube and precipitated with an equal volume of chilled isopropanol + 0.1 volume of 3 M sodium acetate (pH 5) for 2 h at −20 °C. The precipitate was centrifuged in a tabletop centrifuge at 16,000 × *g* for 15 min at 4 °C and washed twice with 70% ethanol. The pellet was resuspended in 135 µl DEPC-treated H_2_O + 15 µl RQ1 DNase buffer and 3 µl RQ1 DNase (Promega). The reaction was incubated at 37 °C for 45 min with an additional 1.5 µl RQ1 DNase added after 30 min. The RNA was purified with the RNA Clean & Concentrator kit (ZymoResearch) according to the manufacturer’s instructions and then eluted in 50 µl DEPC-treated H_2_O. The resulting RNA concentration was measured with a Biospectrometer (Eppendorf).

### cDNA synthesis and qRT-PCR

cDNA synthesis was performed using the SuperScript III First Strand Synthesis system for RT-PCR (Invitrogen) according to the manufacturer’s protocol. In brief, 1 µg of total RNA was combined with 2 µl (50 ng μl^−1^) random hexamers and 2 µl (10 mM) dNTP in a total volume of 20 µl DEPC-treated H_2_O and then incubated at 65 °C for 5 min. The sample was next split into two 10 µl aliquots and combined with 9 µl cDNA synthesis mix (2 µl 10× reverse transcriptase buffer, 4 µl 25 mM MgCl, 2 µl 0.1 M DTT, 1 µl RNase OUT). One aliquot received 1 µl (200 U) Superscript III Reverse Transcriptase while, the other aliquot received 1 µl DEPC-treated H_2_O to serve as a No-RT negative control to assess genomic DNA contamination. The cDNA reactions were incubated at 25 °C for 10 min followed by an additional 1 h incubation at 42 °C. Afterward, 1 µl RNase H (Invitrogen) was added to the reactions and incubated at 37 °C for 20 min. Primers used for qPCR were designed using Primer Express 3.0 software (Applied Biosystems), which selects primers optimized for “delta-delta threshold cycle” (ΔΔ*C*_*T*_) analysis. qPCR was performed using a StepOnePlus Real-Time PCR System (Applied Biosystems) and the reaction mixtures were prepared in triplicate for each sample using the Power SYBR Green PCR Master Mix (Life Technologies). The 16S rRNA gene was used as housekeeping gene reference. Changes in transcript abundance were calculated automatically with the StepOne Software v2.3 using the ΔΔ*C*_*T*_ method, which is briefly described as follows: Δ*C*_*T*_ = *C*_*T*_ (target) − *C*_*T*_ (housekeeping gene); ΔΔ*C*_*T*_ = Δ*C*_*T1*_ − Δ*C*_*T2*_; expression changes are calculated as 2^−ΔΔ*CT*^.

### Biofilm development

In vitro biofilm development was performed using a previously described methodology^41,66^. Briefly, overnight cultures of the *ldh-irvR S. mutans* parent strain and its mutant derivatives were diluted 1:1000 in BTR-G medium supplemented with 0.5% (wt/vol) sucrose and incubated anaerobically at 37 °C for 2 h in wells containing 5 mm diameter HA disks. Following the incubation, the HA disks were loaded into custom 3-D printed coupons, inserted into a Drip Flow biofilm reactor (Biosurface Technologies), and then incubated anaerobically at 37 °C for 16 h with a 0.5 ml min^−1^ flow rate of BTR-G medium supplemented with 0.1% (wt/vol) sucrose.

### Microscopy

Biofilms were developed for 16 h as described above using an *S. mutans gbpC-gfp* transcription fusion reporter strain and its derivatives. The biofilms were subsequently cultured for an additional 4 h ± environmental stress. Following the incubation period, the HA disks were removed from the Drip Flow biofilm reactor and transferred to 1.7 ml microfuge tubes containing 750 µl PBS (pH 7.4). The microfuge tubes were loaded into a cup horn sonicator (QSonica) filled with ice-cold water. Biofilm cells were sonicated at 100% amplitude for 12 cycles of 10 s “on” + 10 s “off” for GFP images or 4 cycles of 10 s “on” + 10 s “off” for the sonic dispersion assay. Images were captured using oil immersion at 100× magnification (1000× total) with an Olympus IX73 inverted epifluorescence microscope and attached Olympus XM10 camera. All fluorescent images were exposed for 300 ms. with identical capture settings and processed using Olympus cellSens software ver. 1.11. All strains and growth conditions assayed via microscopy were imaged using a minimum of three separate fields. Representative images were presented.

### Biofilm rheology

The *ldh-irvR* parent strain and its mutant derivatives were cultured anaerobically at 37 °C in BTR-G medium supplemented with 0.5% (wt/vol) sucrose in static growth conditions for 16 h. to develop biofilms. Biofilms were grown directly onto 8 mm flat rheometer plates and treated ± environmental stress for an additional 4 h. The biofilm-coated rheometer plates were subsequently secured onto the spindle of a Discovery Hybrid Rheometer (DH-R1, TA Instruments), which was then positioned parallel to the flat surface of a Peltier plate, forming a 100 µm gap. The biofilm was then subjected to a dynamic shear test in oscillating mode at 0.05% strain and a 10 Hz shear rate at 25 °C. These parameters were selected to ensure that measurements occurred in the linear viscoelastic regime, where shear thinning would be less likely to occur. For each biofilm sample, data are presented as the average of the shear storage moduli measured during the first 120 s.

## Quantification and statistical analysis

All statistical analyses utilized GraphPad software (https://www.graphpad.com/quickcalcs/) to perform unpaired two-tailed Student’s *t* tests. The threshold for statistical significance was set at a *P* value of *P* ≤ 0.05. All data are presented as the means ± standard deviations derived from a minimum of three biological replicates. Additional details are presented in the figure legends.

### Reporting summary

Further information on research design is available in the Nature Research Reporting Summary linked to this article.

## Supplementary information

Reporting Summary

Supplementary Information

## Data Availability

All data generated or analyzed during this study are included in this published article (and its Supplementary information files).
