# Supplementary material for: Environmental stress perception activates structural remodeling of extant Streptococcus mutans biofilms
Source: NPJ Biofilms Microbiomes. 2020 Mar 27;6:17. doi: 10.1038/s41522-020-0128-z (PMC7101444; doi:10.1038/s41522-020-0128-z)
Supplement: Supplementary file 2 — Supplementary Information [file 41522_2020_128_MOESM2_ESM.pdf]

SUPPLEMENTARY INFORMATION

Supplementary Figures

Supplementary Figure 1

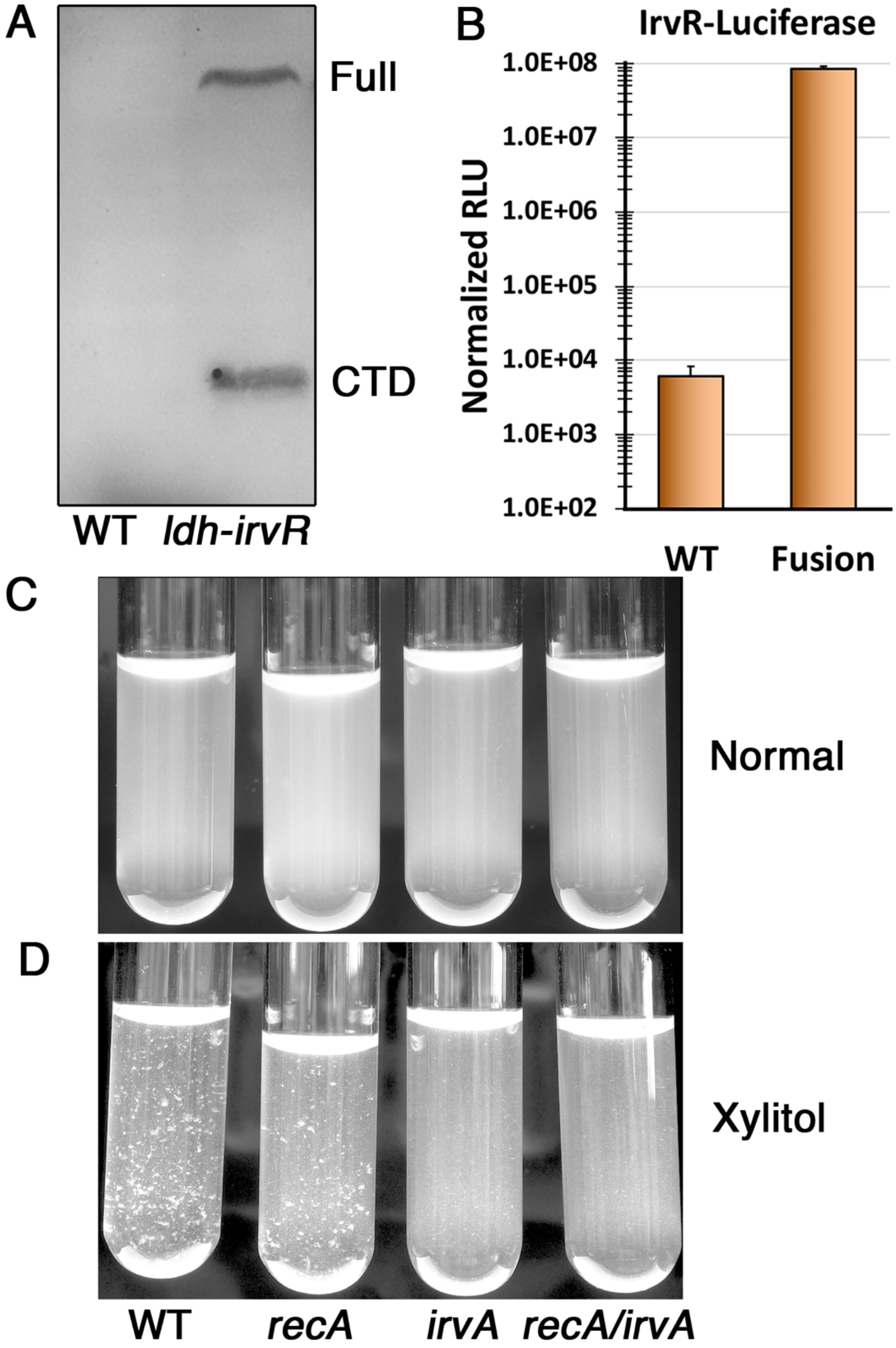

Supp. Fig. 1. Protein abundance and DDAG phenotypes of the wild-type *irvR*. Related to Figure 1. A) A C-terminal FLAG tag was appended onto IrvR and expressed from both its endogenous promoter (WT) as well as the lactate dehydrogenase promoter (*ldh-irvR*). The C-terminal FLAG tag supports immunodetection of the full-length protein (Full) as well as the C-terminal autocleavage fragment (CTD). B) A luciferase assay was used

to quantify production of the IrvR protein. The unmodified wild-type (WT) was compared to an IrvR-luciferase fusion protein (Fusion) reporter strain. The presented luciferase data were normalized by dividing reporter activity values (RLU) by optical density (OD_{600}). C) DDAG assay of wild-type *S. mutans* and its derivatives performed in the absence of environmental stress. The strains from left to right are: parent wild-type (WT), *recA* deletion mutant (Δ*recA*), *irvA* deletion mutant (Δ*irvA*), and *recA/irvA* double deletion mutant (Δ*recA* Δ*irvA*). D) The same strains were assayed in the presence of xylitol stress.

Supplementary Figure 2

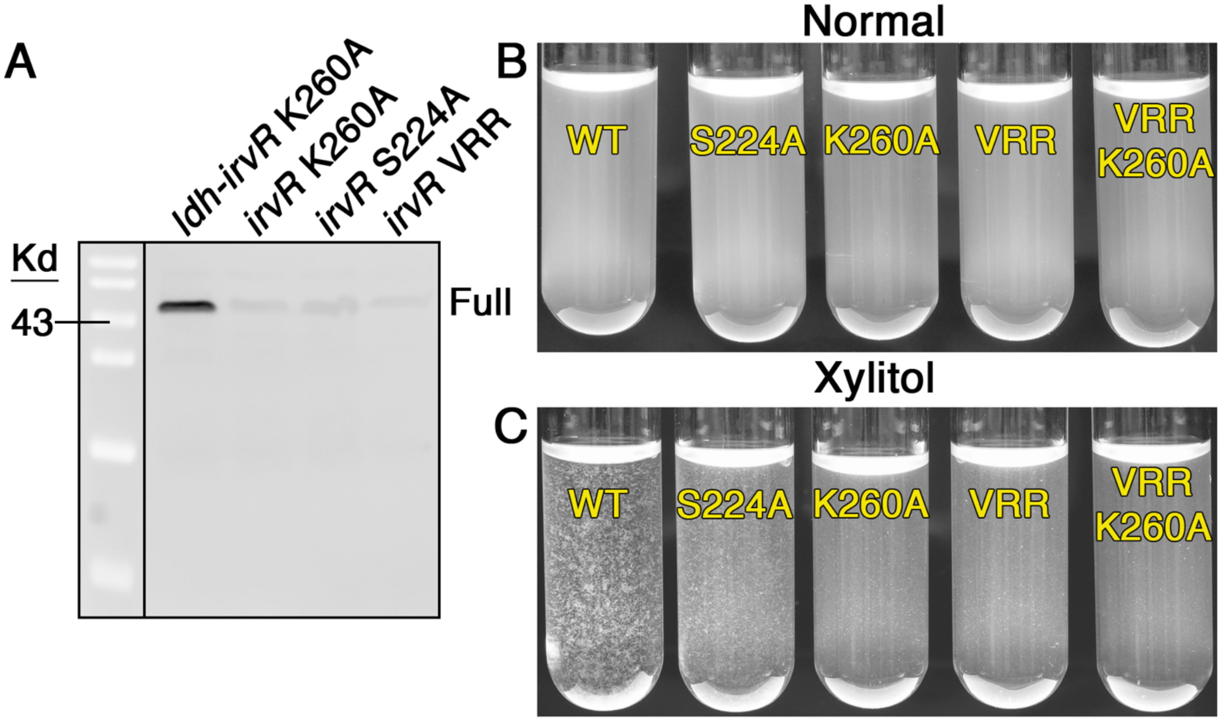

Supp. Fig. 2. Protein abundance and DDAG phenotypes of IrvR autocleavage mutants. Related to Figure 2. A) The protein abundances of various non-cleavable IrvR mutants were compared via western blot. Strains from left to right are: *ldh-irvR* parent strain with IrvR K260A mutation (*ldh-irvR* K260A), wild-type parent strain with IrvR K260A mutation (*irvR* K260A), wild-type parent strain with IrvR S224A mutation (*irvR* S224A), and wild-type parent strain with C-degron/autocleavage site mutation (*irvR* VRR). B) DDAG assay of the wild-type strain and its derivatives performed in the absence of environmental stress. The strains from left to right are: parent wild-type (WT), S224A mutant IrvR (S224A), K260A mutant IrvR (K260A), C-degron/autocleavage site mutant IrvR (VRR), and C-degron/autocleavage site + K260A double mutant IrvR (VRR K260A). C) The same strains were assayed in the presence of xylitol stress.

Supplementary Figure 3

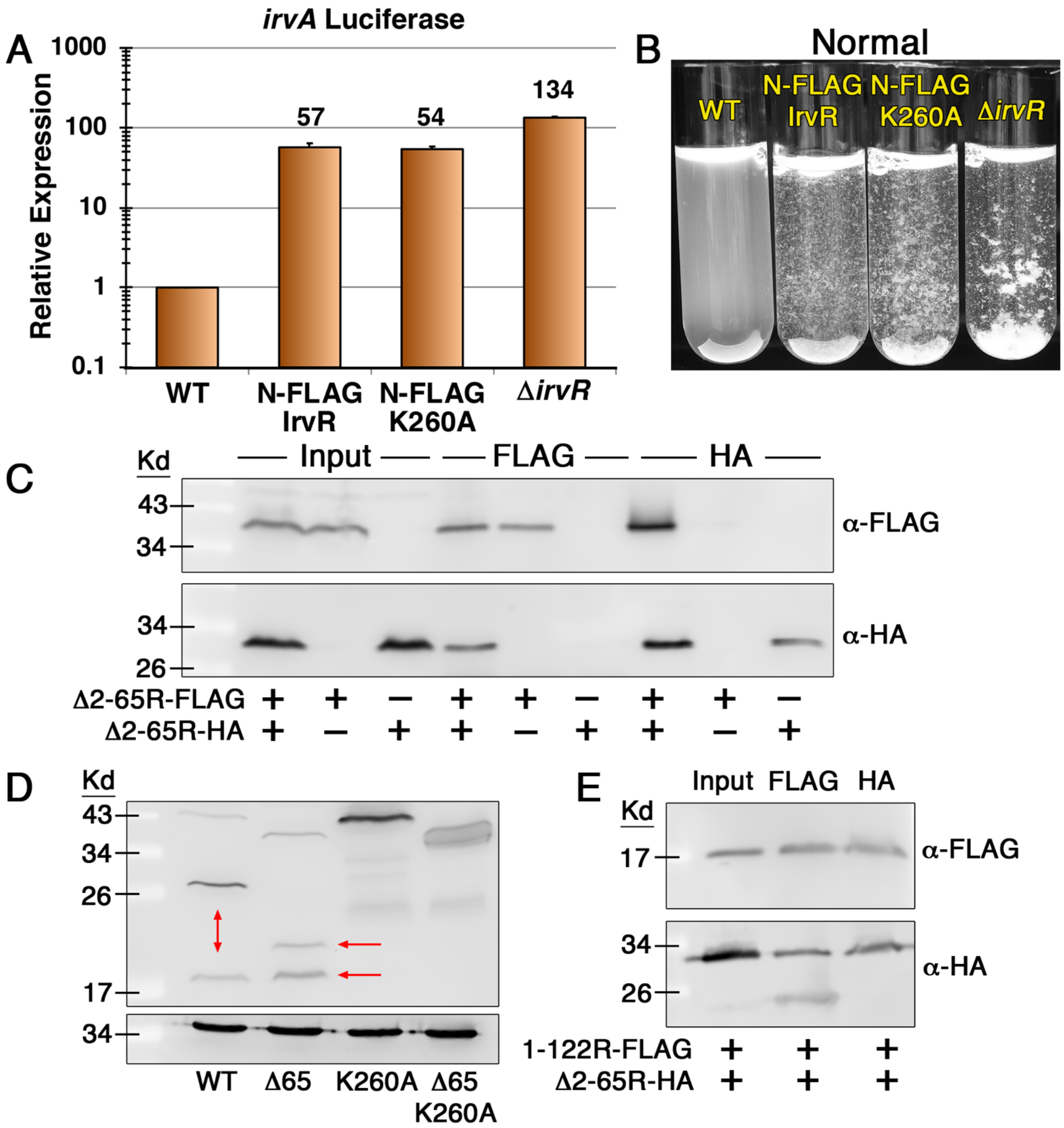

Supp. Fig. 3. Functional role of IrvR N-terminal homodimerization. Related to Figure 3. A) An *irvA-luc* luciferase transcription fusion reporter strain and its derivatives were tested for activity in the absence of added stress. The numbers listed above the bars indicate the fold changes relative to the wild-type reporter, which was arbitrarily assigned a value of 1. Strains from left to right are: parent wild-type reporter strain (WT), N-terminal FLAG tagged IrvR (N-FLAG IrvR), N-terminal FLAG tagged K260A mutant IrvR (N-FLAG K260A), and a Δ*irvR* deletion mutant (Δ*irvR*). B) The same strains were assayed for their DDAG phenotypes in the absence of added stress. C) Coimmunoprecipitation was used to detect homodimerization between N-terminal FLAG and HA tagged IrvR proteins containing deletions of amino acids 2 – 65. Figure columns labeled FLAG and HA indicate the antibody-conjugated resins used for immunopurification, while the rows labeled α-FLAG and α-HA indicate

the antibodies used for western blot detection. D) Western blot was used to detect IrvR autocleavage from the *ldh-irvR* strain and its mutant derivatives, all of which encode an N-terminal IrvR FLAG tag. Red arrows indicate IrvR autocleavage fragments. Strains from left to right: parent *ldh-irvR* strain (WT), deletion of IrvR residues 2 – 65 (Δ65), IrvR K260A mutant (K260A), and deletion of IrvR residues 2 – 65 + K260A mutation (Δ65 K260A). The bottom panel is a lactate dehydrogenase loading control. E) Coimmunoprecipitation was used to detect dimerization between an N-terminal FLAG tagged IrvR containing amino acids 1 – 122 (1-122R-FLAG) and an N-terminal HA tagged IrvR containing a deletion of amino acids 2 – 65 (Δ2-65R-HA).

Supplementary Figure 4

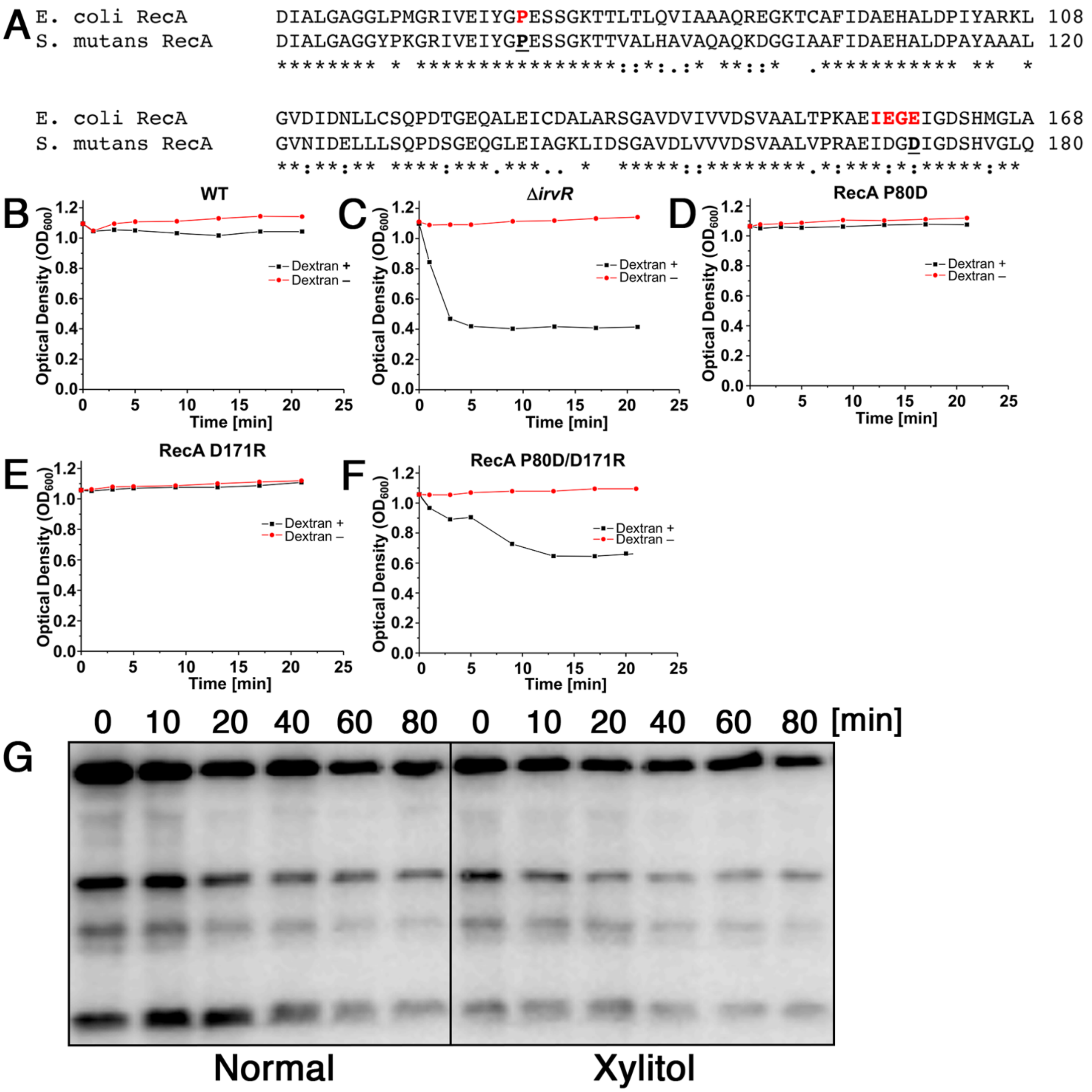

Supp. Fig. 4. DDAG phenotype of a constitutive RecA* mutant and IrvR protein stability. Related to Figure 4. A) The RecA proteins from *E. coli* and *S. mutans* were aligned using the Clustal Omega webserver (https://www.ebi.ac.uk/Tools/msa/clustalo/). Shown here are the partial alignment results with conserved residues indicated by asterisks. Mutations in the *E. coli* residues shown in red font result in a constitutive RecA*

phenotype. The residues mutated in *S. mutans* to create a constitutive RecA* mutant are underlined in bold font. B) A DDAG precipitation assay was used to quantify the effect of candidate *recA* point mutations. A drop in optical density over time is indicative of DDAG-induced cellular aggregates settling out of suspension. Cultures were grown to mid log phase, concentrated to an optical density of OD_{600} ~1.1, vortexed, and then incubated at room temperature in spectrophotometer cuvettes. Optical density measurements were recorded over a time course. G) The protein stability of IrvR was compared between normal and xylitol stress conditions. Cells were cultured to mid log phase before halting transcription and translation with a combination of rifampicin, chloramphenicol, and erythromycin. Cultures were incubated in the presence of the antibiotic cocktail for the times indicated above the respective samples. Afterward, the cells were collected for western blot.

Supplementary Figure 5

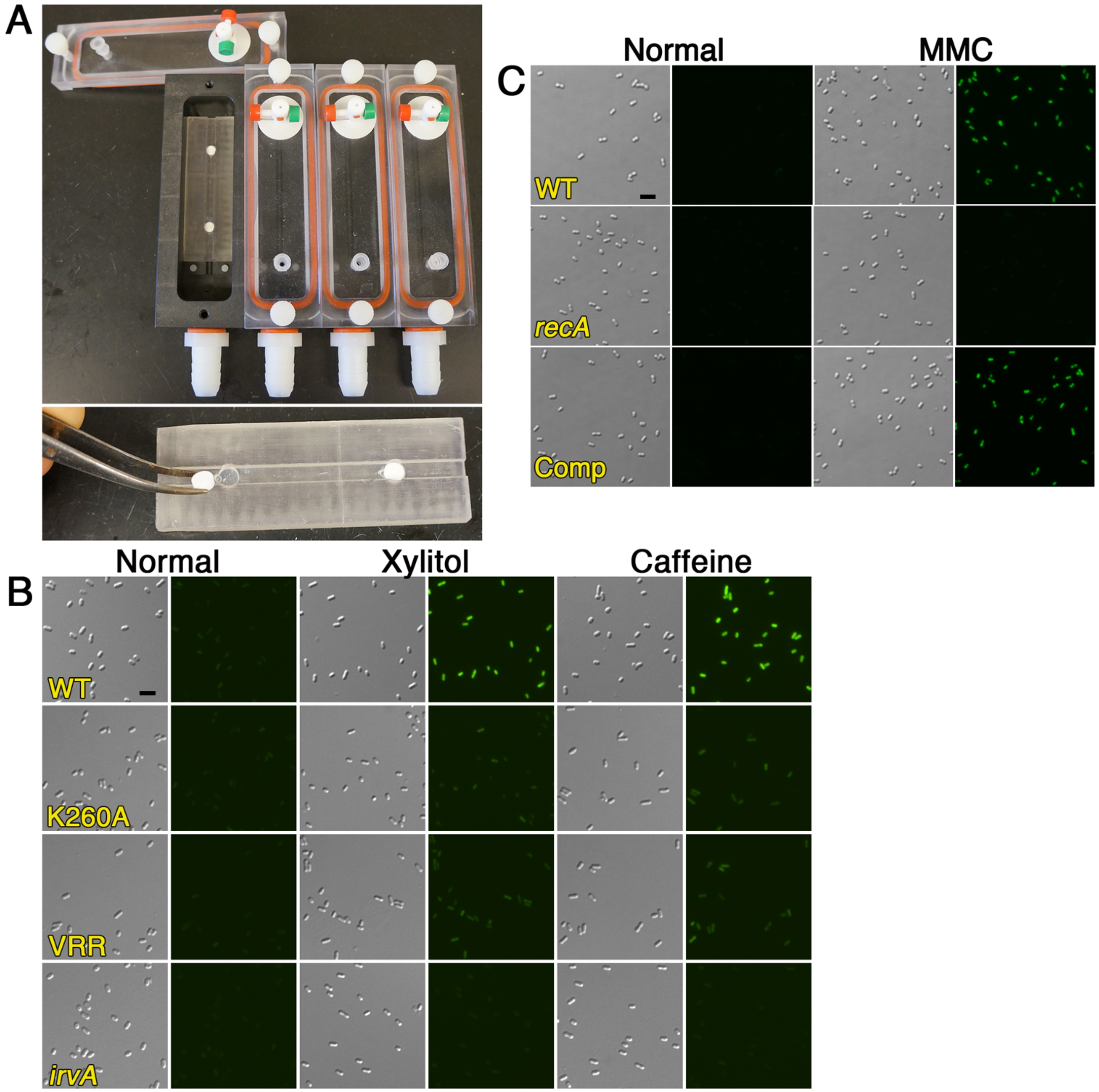

Supp. Fig. 5. IrvR- and RecA-dependent stresses trigger *gbpC* expression within static biofilms. Related to Figure 5. A) Drip flow biofilm reactor system. The bottom image shows the custom 3-D printed inserts used to house the 5 mm diameter hydroxyapatite (HA) disks. To image biofilm cells, the HA disks are removed from the drip flow reactor and then sonicated to disperse the attached cells. B) An *S. mutans gbpC-gfp* transcription fusion reporter strain and its derivatives were cultured in static growth conditions for 16 hr. to develop mature biofilms. The biofilms were subsequently cultured for an additional 4 hr. ± the indicated environmental stresses before dispersing the cells via sonication and then imaging via differential interference contrast microscopy and epifluorescence microscopy. Fluorescent images were captured with identical camera settings and a 300 msec. exposure time. Strains from top to bottom are: parent *gbpC-gfp* reporter strain (WT), IrvR K260A mutant (K260A), C-degron/autocleavage site mutant IrvR (VRR), and Δ*irvA* deletion mutant (*irvA*). C) An identical experimental set up was used to assess the effect of mitomycin C (MMC) stress. Strains from top to bottom are: parent *gbpC-gfp* reporter strain (WT), Δ*recA* deletion mutant (*recA*), and complemented Δ*recA* deletion mutant (Comp). Scalebars indicate 1*μ*m.

Supplementary Figure 6

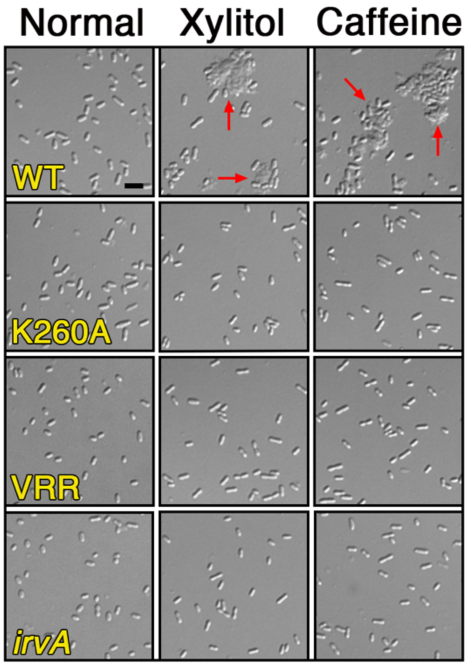

Supp. Fig. 6. Environmental stress impedes sonic dispersion of static biofilms. Related to Figure 6. The *ldh-irvR* strain and its mutant derivatives were cultured for 16 hr. on hydroxyapatite disks in static growth conditions to develop mature biofilms. The biofilms were subsequently cultured for an additional 4 hr. ± the indicated environmental stresses before dispersing the cells using identical sonication conditions and then imaging via differential interference contrast microscopy. Red arrows indicate biofilm fragments resistant to sonication. The strains from top to bottom are: *ldh-irvR* parent strain (WT), IrvR K260A mutant (K260A), C-degron/autocleavage site mutant IrvR (VRR), and Δ*irvA* deletion mutant (*irvA*). Scalebar indicates 1 *μ*m.

Supplementary Tables

Supplementary Table 1. Strains and Plasmids.

Strains	Characteristics	Description	Figures/ Tables	Reference/ Source
S. mutans				
UA159	Wild-type *S. mutans*	Genome reference strain	Fig. S1, S2, S4, Tab. 1	(Ajdic *et al*., 2002)
RF	UA159 *irvR*-FLAG, Km^r	C-terminal FLAG tagged IrvR		
LRF	UA159 *ldh-irvR*-FLAG, Km^r	Constiutive *irvR* overexpression	Fig. 1, 2, 4, 6, S1, S6	This work
LRFrecKO	UA159 Δ*recA, ldh-irvR*-FLAG, Km^r	*recA* deletion, constiutive *irvR* overexpression	Fig. 1, 6	This work
LRFAKO	UA159 Δ*irvA, ldh-irvR*-FLAG, Km^r	*irvA* deletion, constiutive *irvR* overexpression	Fig. 1, 6, S6	This work
LRFArecKO	UA159 Δ*irvA,*Δ*recA, ldh-irvR*-FLAG, Km^r	*irvA* and *recA* double deletion, constiutive *irvR* overexpression	Fig. 1	This work
RF-INT	UA159 *irvR*-FLAG(INT)	Internal FLAG tagged IrvR		This work
RFF	UA159 *irvR*-FLAG(INT)-FLAG, Km^r	Internal and C-terminal FLAG tagged IrvR		This work
LhARFF	UA159 *ldh*-HA-*irvR*-FLAG(INT)-FLAG, Km^r	Internal and C-terminal FLAG tagged IrvR constiutive overexpression	Fig. 1, 4, S4	This work
LhARFFrec KO	UA159 Δ*recA, ldh*-HA-*irvR*-FLAG(INT)-FLAG, Km^r	*recA* deletion, internal and C-terminal FLAG tagged IrvR constiutive overexpression	Fig. 1	This work
LhARFFAK O	UA159 Δ*irvA, ldh*-HA-*irvR*-FLAG(INT)-FLAG, Km^r	*irvA* deletion, internal and C-terminal FLAG tagged IrvR constiutive overexpression	Fig. 1	This work
LhARFFAre cKO	UA159 Δ*irvA,*Δ*recA, ldh*-HA-*irvR*-FLAG(INT)-FLAG, Km^r	*irvA* and *recA* double deletion, internal and C-terminal FLAG tagged IrvR constiutive overexpression	Fig. 1	This work
RrenG	UA159 *irvR-renG*, Sp^r	IrvR-luciferase reporter strain	Fig. S1	This work
recKO-IFD2	UA159 Δ*recA*, Em^r, *p*-Cl-Phe^s	*recA* deletion with IFDC2 cassette		This work
recKO	UA159 Δ*recA*, Em^s, *p*-Cl-Phe^r	markless *recA* deletion	Fig. 4, S1, Tab. 1	This work
AKO	UA159 Δ*irvA*, Em^s, *p*-Cl-Phe^r	*irvA* deletion with IFDC2 cassette	Fig. 4, S1	(Liu *et al*., 2014)

ArecKO	UA159 Δ*recA*, Δ*irvA*, Em^s, *p*-Cl-Phe^r	markless *recA* deletion, *irvA* deletion with IFDC2 cassette	Fig. S1	This work
LRS224A	UA159 *ldh*-*irvR*-FLAG S224A, Km^r	Constiutive *irvR* S224A overexpression	Fig. 2	This work
LRK260A	UA159 *ldh*-*irvR*-FLAG K260A, Km^r	Constiutive *irvR* K260A overexpression	Fig. 2, 6, S2, S6	This work
LRVRR	UA159 *ldh*-*irvR*-FLAG VRR, Km^r	Constiutive *irvR* VRR overexpression	Fig. 2, 6, S6	This work
LRK260AVRR	UA159 *ldh*-*irvR*-FLAG K260A VRR, Km^r	Constiutive *irvR* K260A VRR overexpression	Fig. 2	This work
LRFFS224A	UA159 *ldh*-HA-*irvR*-FLAG-FLAG S224A, Em^s, *p*-Cl-Phe^r, Km^r	Internal and C-terminal FLAG tagged *irvR* S224A overexpression	Fig. 2	This work
LRFFK260A	UA159 *ldh*-HA-*irvR*-FLAG-FLAG K260A, Em^s, *p*-Cl-Phe^r, Km^r	Internal and C-terminal FLAG tagged *irvR* K260A overexpression	Fig. 2	This work
LRFFVRR	UA159 *ldh*-HA-*irvR*-FLAG-FLAG VRR, Em^s, *p*-Cl-Phe^r, Km^r	Internal and C-terminal FLAG tagged *irvR* VRR overexpression	Fig. 2	This work
LRFFK260A VRR	UA159 *ldh*-HA-*irvR*-FLAG-FLAG K260A VRR, Em^s, *p*-Cl-Phe^r, Km^r	Internal and C-terminal FLAG tagged *irvR* K260A VRR overexpression	Fig. 2	This work
ARKO-IFD2	UA159 Δ*irvRA*, Em^r, *p*-Cl-Phe^s	*irvRA* deletion with IFDC2 cassette		
RS224A	UA159 IrvR S224A, Em^s, *p*-Cl-Phe^r	IrvR S224A point mutant	Fig. S2	This work
RK260A	UA159 IrvR K260A, Em^s, *p*-Cl-Phe^r	IrvR K260A point mutant	Fig. S2	This work
RVRR	UA159 IrvR VRR, Em^s, *p*-Cl-Phe^r	IrvR VRR point mutant	Fig. S2, Tab. 1	This work
RK260AVRR	UA159 IrvR K260A&VRR, Em^s, *p*-Cl-Phe^r	IrvR K260A&VRR double point mutant	Fig. S2	This work
IFDC3	UA140 pheS T260S/A314G	IFDC3 cassette template		
F-R/H-R	*ldh*-FLAG-*irvR* K260A, *gapC*-HA-*irvR* K260A, Δ*irvAR*	*irvRA* deletion, overexpression of FLAG tagged IrvR K260A and HA tagged IrvR K260A	Fig. 3	This work
F-R66-122KO/H-R	*ldh*-FLAG-*irvR* Δ66-122 K260A, *gapC*-HA-*irvR* K260A, Δ*irvAR*	*irvRA* deletion, overexpression of FLAG tagged IrvR Δ66-122 K260A and HA tagged IrvR K260A	Fig. 3	This work

LFRF	*ldh*-FLAG-*irvR*-FLAG, *ldh*-HA, Sp^r	N-terminal and C-terminal FLAG tagged IrvR overexpression, HA tagged lactate dehydrogenase (Ldh)	Fig. 3, S3	This work
LFRF66-122KO	*ldh*-FLAG-*irvR* Δ66-122-FLAG, *ldh*-HA, Sp^r	N-terminal and C-terminal FLAG tagged IrvR Δ66-122 overexpression, HA tagged Ldh	Fig. 3	This work
F-R66-80KO/H-R	*ldh*-FLAG-*irvR* Δ66-80 K260A, *gapC*-HA-*irvR* K260A, Δ*irvAR*	*irvRA* deletion, overexpression of FLAG tagged IrvR Δ66-80 K260A and HA tagged IrvR K260A	Fig. 3	This work
F-R81-94KO/H-R	*ldh*-FLAG-*irvR* Δ81-94 K260A, *gapC*-HA-*irvR* K260A, Δ*irvAR*	*irvRA* deletion, overexpression of FLAG tagged IrvR Δ81-94 K260A and HA tagged IrvR K260A	Fig. 3	This work
F-R95-108KO/H-R	*ldh*-FLAG-*irvR* Δ95-108 K260A, *gapC*-HA-*irvR* K260A, Δ*irvAR*	*irvRA* deletion, overexpression of FLAG tagged IrvR Δ95-108 K260A and HA tagged IrvR K260A	Fig. 3	This work
F-R109-122KO/H-R	*ldh*-FLAG-*irvR* Δ109-122 K260A, *gapC*-HA-*irvR* K260A, Δ*irvAR*	*irvRA* deletion, overexpression of FLAG tagged IrvR Δ109-122 K260A and HA tagged IrvR K260A	Fig. 3	This work
ArenG	UA159 *irvA-renG*, Sp^r	IrvA-luciferase reporter strain	Fig. S3	This work
ArenG/FR	UA159 *irvA-renG*, N-FLAG IrvR, Sp^r	N-terminal FLAG tagged IrvR, IrvA-luciferase reporter strain	Fig. S3	This work
ArenG/FRK 260A	UA159 *irvA-renG,* N-FLAG IrvR K260A, Sp^r	N-terminal FLAG tagged IrvR K260A, IrvA-luciferase reporter strain	Fig. S3	This work
ArenG/RKO	UA159 *irvA-renG,* Δ*irvR*, Sp^r	*irvR* deletion, IrvA-luciferase reporter strain	Fig. S3	This work
RF65KO/R H65KO	*ldh*-FLAG-*irvR* Δ2-65 K260A, *gapC*-HA-*irvR* Δ2-65 K260A, Δ*irvAR*	*irvRA* deletion, overexpression of FLAG tagged IrvR Δ2-65 K260A and HA tagged IrvR Δ2-65 K260A	Fig. S3	This work
LFRF65KO	*ldh*-FLAG-*irvR* Δ2-65-FLAG, *ldh*-HA, Sp^r	N-terminal and C-terminal FLAG tagged IrvR Δ2-65 overexpression, HA tagged Ldh	Fig. S3	This work
LFRFK260A	*ldh*-FLAG-*irvR* K260A-FLAG, *ldh*-HA, Sp^r	N-terminal and C-terminal FLAG tagged IrvR K260A overexpression, HA tagged Ldh	Fig. S3	This work
LFRFK260A 65KO	*ldh*-FLAG-*irvR* Δ1-65 -FLAG, K260A, *ldh*-HA, Sp^r	N-terminal and C-terminal FLAG tagged IrvR Δ1-65 K260A overexpression, HA tagged Ldh	Fig. S3	This work

LFR122KO/ RH65KO	*ldh*-FLAG-*irvR* 1-122, *gapC*-HA-*irvR* Δ2-65 K260A, Δ*irvAR*	*irvRA* deletion, overexpression of FLAG tagged IrvR 1-122 and HA tagged IrvR Δ2-65 K260A	Fig. S3	This work
CKOLR	UA159 Δ*gbpC, ldh-irvR*-FLAG, Km^r	*gbpC* deletion, constiutive irvR overexpression	Fig. 4	This work
RKO	UA159 Δ*irvR*, Em^s, *p*-Cl-Phe^r	markless *irvR* deletion,	Fig. 4, S4	This work
LRFrec#	UA159 *recA* P80D D171R, *ldh-irvR*-FLAG, Km^r	*recA* P80D D171R double point mutant, constiutive *irvR* overexpression	Fig. 4, 6	This work
LRFrec#AKO	UA159 Δ*irvA*, *recA* P80D D171R, *ldh-irvR*-FLAG, Km^r	*irvA* deletion, *recA* P80D D171R double point mutant, constiutive *irvR* overexpression	Fig. 4	This work
CKO	UA159 Δ*gbpC*, Em^s, *p*-Cl-Phe^r	markless *gbpC* deletion	Fig. 4	(Liu *et al.*, 2014)
LRFrec#CKO	UA159 Δ*gbpC*, *recA* P80D D171R, *ldh-irvR*-FLAG, Km^r	*gbpC* deletion, *recA* P80D D171R double point mutant, constiutive irvR overexpression	Fig. 4	This work
recComp	UA159 $gyrA_P$::*recA*, Δ*recA*, Sp^r	*recA* complementation		This work
LRFrecComp	UA159 $gyrA_P$::*recA*, Δ*recA*, *ldh-irvR*-FLAG, Sp^r, Km^r	*recA* complementation, constiutive irvR overexpression	Fig. 4, 6	This work
recP80D	UA159 *recA* P80D, Em^s, p-Cl-Phe^r	*recA* P80D point mutant	Fig. S4	This work
recD171R	UA159 *recA* D171R, Em^s, p-Cl-Phe^r	*recA* D171R point mutant	Fig. S4	This work
recA#	UA159 *recA* P80D D171R, Em^s, p-Cl-Phe^r	*recA* P80D D171R double point mutant	Fig. S4	This work
CGFP	UA159 $gbpC_P$-*gbpC*::*gfp*, Em^s, *p*-Cl-Phe^r	GFP tagged GbpC	Fig. 5, S5	This work
CGFPK260A	UA159 $gbpC_P$-*gbpC*::*gfp*, IrvR K260A, Em^s, *p*-Cl-Phe^r	GFP tagged GbpC, IrvR K260A point mutant	Fig. 5, S5	This work
CGFPVRR	UA159 $gbpC_P$-*gbpC*::*gfp*, IrvR VRR, Em^s, *p*-Cl-Phe^r	GFP tagged GbpC, IrvR VRR point mutant	Fig. 5, S5	This work
CGFPAKO	UA159 $gbpC_P$-*gbpC*::*gfp*, Δ*irvA*, Em^s, *p*-Cl-Phe^r	GFP tagged GbpC, *irvA* deletion	Fig. 5, S5	This work
CGFPrecKO	UA159 $gbpC_P$-*gbpC*::*gfp*, Δ*recA*, Em^s, *p*-Cl-Phe^r	GFP tagged GbpC, *recA* deletion	Fig. 5, S5	This work

CGFPrecA Comp	UA159 *gbpC*$_P$-*gbpC*::*gfp*, *gyrA*$_P$::*recA*, Δ*recA*, Sp^r, Em^s, *p*-Cl-Phe^r	GFP tagged GbpC, *recA* complementation	Fig. 5, S5	This work
**Plasmids**				
pDL278		*E. coli*-*Streptococcus* shuttle vector, Sp^r		(Chen and LeBlanc, 1992)
pWVKTs		Harboring kanamycin resistance cassette *aphAIII*, Km^r		(Gutierrez *et al.*, 1996)

Strains and plasmids used in this study are described with their relevant genotypes. The Figure column indicates the manuscript figures utilizing the respective strains. Antibiotic resistances are abbreviated as follows: Em^r, erythromycin resistance; p-Cl-Phe^r, 4-chlorophenylalanine resistance; Sp^r, spectinomycin resistance; and Km^r, kanamycin resistance.

Supplementary Table 2. Primers.

Primer	Sequence (5'→3')	Purpose
irvR-fwd3	CTTCTAGCTGAGAGCGGAATGAAAC	*irvR*-3xFLAG (native irvR promoter, tagged at C-terminus)
irvR-FLAG-r1	CGTGATCCTTATAGTCTCCATCATGGTCTTTGTAGTCGGTAATGACCGTTCCGATGAC	*irvR*-3xFLAG (native irvR promoter, tagged at C-terminus)
irvR-FLAG-r2	CTTATCGTCATCGTCCTTGTAATCGATGTCGTGATCCTTATAGTCTCCATCATGGTC	*irvR*-3xFLAG (native irvR promoter, tagged at C-terminus)
irvR+*kan*R-FLAG_dn-f	CACGACATCGATTACAAGGACGATGACGATAAGTAAGAGGATGAAGAGGATGAGGAGG	*irvR*-3xFLAG (native irvR promoter, tagged at C-terminus)
*kan*R-rev	CTCGGGACCCCTATCTAGCG	*irvR*-3xFLAG (native irvR promoter, tagged at C-terminus)
irvR+*kan*R_dn-f	CGCTAGATAGGGGTCCCGAGAAATTAAAGAAAGTCCCATAAAAGACACTTG	*irvR*-3xFLAG (native irvR promoter, tagged at C-terminus)
SMU1400c-fwd	CGTGCCTGAGTGGTTGATAC	*irvR*-3xFLAG (native irvR promoter, tagged at C-terminus)
ldh-fwd	GTTGGTGATGGTGCTGTAGG	adding the *ldh*-RBS directly upstream of *irvR* coding region
ldh+RBS_dn-r	GTTCTAAACATCTCCTTATTAGTTACGAG	adding the *ldh*-RBS directly upstream of *irvR* coding region
irvR-*ldh*-OL_dn-f	CTCGTAACTAATAAGGAGATGTTTAGAACATGGGACGCGGTAAATTAACTCC	adding the *ldh*-RBS directly upstream of *irvR* coding region
*kan*R-rev	CTCGGGACCCCTATCTAGCG	adding the *ldh*-RBS directly upstream of *irvR* coding region
ldh-*irvR*-FLAG-dn	CGCTAGATAGGGGTCCCGAGACAATAAAAATCCATAAAAATACCAAGCATTG	adding the *ldh*-RBS directly upstream of *irvR* coding region
ldh-rev	CCACAGCAACGTCATCTCTCC	adding the *ldh*-RBS directly upstream of *irvR* coding region

recA-fwd	GATCCCACAATTGCTCCTTATGC	*recA* mutagenesis
recA-rev	CGATAAGGAGCCGTTAATGCTTC	*recA* mutagenesis
IFDC2-u_f	CCGAGCAACAATAACACTCATAGC	*recA* mutagenesis
IFDC2-rev	GATAAATTATTAGGTATACTACTGACAGCTTC	*recA* mutagenesis
recA-IFDC2-d_f	GATAAATTATTAGGTATACTACTGACAGCTTCTAAATAAAAATAGGTCCCAAGACCG	*recA* mutagenesis
recA-IFDC2-u_r	GCTATGAGTGTTATTGTTGCTCGGTTAATCTTCAAGCTCAATTGTATCATCC	*recA* mutagenesis
*recA*ml-fwd	GCTTTAAAACATAGAATAGGAGAAAAAGATTGATTAAATAAAAATAGGTCCCAAGACCG	*recA* mutagenesis
*recA*ml-rev	CGGTCTTGGGACCTATTTTTATTTAATCAATCTTTTTCTCCTATTCTATGTTTTAAAGC	*recA* mutagenesis
Int-*irvR*-FLAG-up_r1	CCTTATAATCACCATCGTGATCCTTATAATCTCCAAAGGTTTGATACAAATCCTTAGC	*irvR*-3xFLAG (internal – native promoter IrvRDBD-3xFLAG)
Int-*irvR*-FLAG-up_r2	GTCATCTTTATAGTCAATATCGTGATCCTTATAATCACCATCGTGATCCTTATAATCTC	*irvR*-3xFLAG (internal – native promoter IrvRDBD-3xFLAG)
Int-*irvR*-FLAG-f1	GATATTGACTATAAAGATGACGATGACAAGCGTCAATACGAAAACATTCAGATTTATGG	*irvR*-3xFLAG (internal – native promoter IrvRDBD-3xFLAG)
Int-*irvR*-FLAG-f2	TTATAAGGATCACGATATTGACTATAAAGATGACGATGACAAGCG	*irvR*-3xFLAG (internal – native promoter IrvRDBD-3xFLAG)
ldh-fwd	GTTGGTGATGGTGCTGTAGG	*ldh*-HA, *ldh*-*irvR*-FLAG-FLAG
ldh-rev2	GAGGAACGTGGTATGGTTATGTTG	*ldh*-HA, *ldh*-*irvR*-FLAG-FLAG
ldh-HA-up_r	TTAAGCGTAGTCTGGAACGTCGTATGGGTAGTTACGAGCTGCAGCAGCAA	*ldh*-HA, *ldh*-*irvR*-FLAG-FLAG
FLAG-*irvR*-r1	CTTATAATCACCATCGTGATCCTTATAATCCATGTTCTAAACATCTCCTTATTAAGCG	*ldh*-HA, *ldh*-*irvR*-FLAG-FLAG
FLAG-*irvR*-r2	CGTCATCTTTATAGTCAATATCGTGATCCTTATAATCACCATCGTGATCCTTATAATCC	*ldh*-HA, *ldh*-*irvR*-FLAG-FLAG
FLAG-*irvR*-f	GGATCACGATATTGACTATAAAGATGACGATGACAAGGGACGCGGTAAATTAACTCCAC	*ldh*-HA, *ldh*-*irvR*-FLAG-FLAG
ldh-rbs-flag-r1	ATAATCCATGTTCTAAACATCTCCTTATTAGTTACGAGCTGCAGCAGCAAATTCTT	*ldh*-FLAG-*irvR*-FLAG
ldh-rbs-flag-r2	TAATCACCATCGTGATCCTTATAATCCATGTTCTAAACATCTCCTTATTAG	*ldh*-FLAG-*irvR*-FLAG
FLAG-*irvR*-f1	TAAGGATCACGATGGTGATTATAAGGATCACGATATTGACTATAAAGATGACGATGAC	*ldh*-FLAG-*irvR*-FLAG
FLAG-*irvR*-f2	ACGATATTGACTATAAAGATGACGATGACAAGGGACGCGGTAAATTAACTCCACA	*ldh*-FLAG-*irvR*-FLAG
ldh-HA-up_r	TTAAGCGTAGTCTGGAACGTCGTATGGGTAGTTACGAGCTGCAGCAGCAA	*ldh*-HA fusion
ldh-HA-dn_f	CCGATTAAGATACTGCCTACTAACTCCACAATAAAAATCCATAAAAATACCAAGCATTG	*ldh*-HA fusion
ldh-HA-dn_r	CGTATGTATTCAAATATATCCTCCTCAC	*ldh*-HA fusion
irvA-rev1	GCCAAACCTTGTGCAACTTC	*irvR*-*renG* fusion

irvR-N-*RenG*-up_r	CCACTTCCACCACCTCCACTTCCACCACCTCCTCCAAAGGTTTGATACAAATCCTTAGC	*irvR-renG* fusion
irvR-N-*RenG*-up_r2	TCAGGATCATAAACTTTACTAGCCATACTTCCACCACCTCCACTTCCACCACCTCCACT	*irvR-renG* fusion
IrvR-*RenG*-dn_f	TGGAGGTGGTGGAAGTGGAGGTGGTGGAAGTATGGCTAGTAAAGTTTATGATCCTGAAC	*irvR-renG* fusion
RenG-linker-rev	CCACCTCCGATAGAACGTTGCTCATTCTTCAAAAC	*irvR-renG* fusion
irvR-C-*RenG*-dn_f2	GAAGAATGAGCAACGTTCTATCGGAGGTGGTGGAAGTGGAGGTGGTGGAAGTGGAGG	*irvR-renG* fusion
irvR-C-*RenG*-dn_f	TGGAGGTGGTGGAAGTGGAGGTGGTGGAAGTCGTCAATACGAAAACATTCAGATTTATG	*irvR-renG* fusion
irvR-*Spec*-up_r	CTCCTCATCCTCTTCATCCTCTTCTTAGGTAATGACCGTTCCGATG	*irvR-renG* fusion
irvR S224A F	CAATGGTAATGCTATGGAACCTTTATT	point mutation
irvR S224A R	CCGTTGTAAAATAAAGGTTCCATAGC	point mutation
irvR K260A F	ATGTCGCAAAACTCTACCGAAAAAACAAAG	point mutation
irvR K260A R	CTTTGTTTTTTCGGTAGAGTTTTGCGACAT	point mutation
irvR VRR F	GGTGAAGTTAGACGTGGTACTGGTATCTGGG	point mutation
irvR VRR R	CCCAGATACCAGTACCACGTCTAACTTCACC	point mutation
gapC-fwd	CACTTACATGCTAATGGTGGAGC	*gapC*-HA-*irvR*-HA
gapC-IFDC2-rev	ATGAGTGTTATTGTTGCTCGGTTATTTAGCGATTTTTGCAAAGTACTC	*gapC*-HA-*irvR*-HA
IFDC2-down-f	AGGTATACTACTGACAGCTTCTACTCATTATTTTGTAAAAGGGGACC	*gapC*-HA-*irvR*-HA
gapC-rev	GCCAAATCAGCAGCTACAGG	*gapC*-HA-*irvR*-HA
gapC+RBS_dn-r1	GTACATTAGTGATTTCCTCCTTATGTTATTTAGCGATTTTTGCAAAGTACTC	*gapC*-HA-*irvR*-HA
RBS-HA_dn-R2	AGCGTAGTCTGGTACATCGTATGGGTACATTAGTGATTTCCTCCTTATG	*gapC*-HA-*irvR*-HA
HA-*irvR*-f	CATACGATGTACCAGACTACGCTGGACGCGGTAAATTAACTCCACA	*gapC*-HA-*irvR*-HA
irvR-down-r	TCATCGGAACGGTCATTACCTAATACTCATTATTTTGTAAAAGGGGACC	*gapC*-HA-*irvR*-HA
ldh N *irvR*-3-F1	TTACTCAAGTTATAGAATCAGGC	IrvR 66-122 truncation
ldh N *irvR*-3-R1	TATGAGTGTTATTGTTGCTCGGTGCGATTTTTTGAATATTCCCTAT	IrvR 66-122 truncation
ldh N *irvR*-3-F3	TAGGTATACTACTGACAGCTTCGAATGAAACAATCTCACTTAGCT	IrvR 66-122 truncation
ldh N *irvR*-3-R3	CGTTTCATCACTGGCAGACAATGCGATTTTTTGAATATTCCCTAT	IrvR 66-122 truncation
ldh N *irvR*-3-F4	ATCGCATTGTCTGCCAGTGATGAAACG	IrvR 66-122 truncation

ldh N *irvR*-3-R4	CAGGAGACATTCCTTCCGTAT	IrvR 66-122 truncation
66-80 R3	GAGTAGAAGAGTCCTTTTTACTTGCGATTT TTTGAATATTCCCTAT	IrvR 66- 80 truncation
66-80 F4	AGTAAAAAGGACTCTTCTACTC	IrvR 66- 80 truncation
81-94 R1	TATGAGTGTTATTGTTGCTCGGTGCAAATC TAGGGTCAATATCG	IrvR 81-94 truncation
81-94 F3	TAGGTATACTACTGACAGCTTCCTTAACAT TCCTACAAGTAGTCT	IrvR 81-94 truncation
81-94 R3	CTCTTCATTTCTAATTGTTTGGCTGCAAAT CTAGGGTCAATATCG	IrvR 81-94 truncation
81-94 F4	GCCAAACAATTAGAAATGAAGAG	IrvR 81-94 truncation
95-108 F3	TAGGTATACTACTGACAGCTTCGAGTTGCC AAAAATAGGGAATA	IrvR 95-108 truncation
95-108 R1	TATGAGTGTTATTGTTGCTCGGAATATGAT TAATTTTATCAAGAGTAG	IrvR 95-108 truncation
95-108 R3	TTCTTTTGATTGTTGGTAACAGAAAATATG ATTAATTTTATCAAGAGTAG	IrvR 95-108 truncation
95-108 F4	TTCTGTTACCAACAATCAAAAGAA	IrvR 95-108 truncation
109-122 R1	TATGAGTGTTATTGTTGCTCGGATCTAAGA CTTTTATCTGTCTCTT	IrvR 109-122 truncation
109-122 F3	TAGGTATACTACTGACAGCTTCCGCAGATT ACTTTGGTTTACAA	IrvR 109-122 truncation
109-122 R3	CGTTTCATCACTGGCAGACAAATCTAAGAC TTTTATCTGTCTCTT	IrvR 109-122 truncation
irvA-fwd1	GTCCAACCCAGATACCAGTACC	*irvA-renG* fusion
*irvA_renG*_up-r	CTAGCCATTCTCTAAACATCTCCTTCTTAC CAAAAAAAAATAGAGTCTAAACTGATA	*irvA-renG* fusion
RenG-F	GAAGGAGATGTTTAGAGAATGGCTAGTA	*irvA-renG* fusion
*renG_Spc*_dn-r	CTCCTCATCCTCTTCATCCTCTTCTTAGAT AGAACGTTGCTCATTCTTCAAAAC	*irvA-renG* fusion
pDL278-fwd	GAAGAGGATGAAGAGGATGAGGAG	*irvA-renG* fusion
pDL278-rev	GGAGTTAGTAGGCAGTATCTTAATCGG	*irvA-renG* fusion
*irvA_renG*_dn_f	GATTAAGATACTGCCTACTAACTCCAAATT CCGATTTAAAACAGAAATTCAAAAATAAG	*irvA-renG* fusion
irvA-rev1	GCCAAACCTTGTGCAACTTC	*irvA-renG* fusion
recA-P80D-fwd	GATGAATCTTCTGGTAAGACAACTGTCGC	point mutation
recA-P80D-rev	GCGACAGTTGTCTTACCAGAAGATTCATCC CCGTAAATCTCAACGATACGCC	point mutation
recA-D171R-fwd	CGTATTGGTGATAGTCATGTTGGCTTACAA GC	point mutation
recA-D171R-rev	GCCAACATGACTATCACCAATACGTCCGT CAATCTCCGCACGTGG	point mutation
gyrA-recA F1	AATGATGATGACTTTGTTCGTGA	*recA* complementation
gyrA-recA R1	TTATACTAAATTTTGCTAGCTACTCATTTTC ATTAT	*recA* complementation

gyrA-recA F2	GAGTAGCTAGCAAAATTTAGTATAATGAAAAGACGGC	*recA* complementation
gyrA-recA R2	ATTGTCCTGGGTTAATCTTCAAGCTCAATTGTATC	*recA* complementation
gyrA-recA F3	GCTTGAAGATTAA CCCAGGACAATAACCTTATAGC	*recA* complementation
gyrA-recA R3	GTTCTTTTTTCACTAGAGTCGATACAAATTCCTCGTAGGC	*recA* complementation
gyrA-recA F4	GTATCGACTCTAGTGAAAAAAGAACGTCAATCTAGG	*recA* complementation
gyrA-recA R4	AGCGTACGTACTATTAGACATAT	*recA* complementation
gbpC-gfp F1	GGCTAAGGACAACCAATATAAAG	*gbpC-gfp* fusion
gbpC-gfp R1	ATGAGTGTTATTGTTGCTCGGCTAGTTTTCTTTTTTTCTAGCTGC	*gbpC-gfp* fusion
gbpC-gfp F3	AGGTATACTACTGACAGCTTCAGTGTTAATTTCTATAAAAAGAGGTT	*gbpC-gfp* fusion
gbpC-gfp R3	TATTATCTAACAGTTGTTCTAAGAC	*gbpC-gfp* fusion
gbpC-gfp F4	GGCATGGATGAGCTTTATAAGTAAAGTGTTAATTTCTATAAAAAGAGGTT	*gbpC-gfp* fusion
gbpC-Pg*gbpC*-*spgfp* R4 (Pg R4)	CTCCTTTTGACATAAAAACCATCCTTTCTAGTTTTCTTTTTTTCTAGCTGC	*gbpC-gfp* fusion
sp gfp F	AAAGGATGGTTTTTATGTCAAAAGGAG	*gbpC-gfp* fusion
gfp R	TTACTTATAAAGCTCATCCATGCC	*gbpC-gfp* fusion

Supplementary Methods

DNA manipulation and strain construction

The strains used in this study are described in Table S1, while the primers used for PCR are listed in Table S2. The *S. mutans* genome reference strain UA159 was employed as the wild-type parental strain for all *S. mutans* strains described in this study.

Construction of epitope tagged IrvR

To add a C-terminal FLAG epitope onto IrvR for the detection of the CTD autocleavage fragment, the *irvR* open reading frame (ORF) was amplified from strain UA159 using the primer pair *irvR*-fwd3 & *irvR*-FLAG-r1/*irvR*-FLAG-r2, which add the FLAG ORF onto the 3' of the *irvR* amplicon. The kanamycin resistance cassette was amplified from plasmid pWVKTs with the primer pair *irvR*+KanR-FLAG_dn-f/kanR-rev, while the downstream homologous fragment amplified from UA159 with the primer pair *irvR*+kanR_dn-f/*SMU1400*c-fwd. These PCR amplicons were mixed and assembled via overlap extension PCR (OE-PCR) with the primers *irvR*-fwd3/*SMU1400c*-fwd. The OE-PCR amplicon was transformed into strain UA159 and selected on agar plates supplemented with kanamycin to create the FLAG tagged strain RF. To construct an *irvR* transcription fusion to the constitutively expressed lactate dehydrogenase gene *ldh*, we inserted an *irvR* ORF + 3' FLAG ORF immediately downstream of the chromosomal copy of the *ldh* ORF. The upstream *ldh* flanking region was amplified from UA159 with the primer pairs *ldh*-fwd/*ldh*+RBS_dn-r. The FLAG-encoding *irvR* ORF + kanamycin resistance cassette was amplified from strain RF with the primer pair *irvR*-*ldh*-OL_dn-f/kanR-rev. The downstream homologous fragment was amplified from UA159 with the primer pair *ldh*-*irvR*-FLAG-dn/*ldh*-rev. The three resulting PCR amplicons were mixed and assembled via OE-PCR with the primer pair *ldh*-fwd/*ldh*-rev. The OE-PCR amplicon was transformed into UA159 and selected on agar plates supplemented with 850 μg ml-

^{1} kanamycin to create the strain LRF. To construct *irvA*, *recA*, and *irvA/recA* double mutants in the *ldh-irvR* fusion background, each of the following described *irvA* and *recA* mutagenesis protocols was repeated using the LRF fusion strain as the parental strain.

Construction recA *deletions for DDAG analysis*
The *recA* deletion mutant was generated using a previously described 2-step markerless mutagenesis protocol (Xie et al., 2011). The counterselectable resistance cassette IFDC2 was inserted immediately downstream of the *recA* ORF by transforming an OE-PCR product containing the IFDC2 cassette flanked by sequences homologous the *recA* locus. The IFDC2 cassette was amplified from plasmid pIFDC2 (Xie et al., 2011) using the primer pair IFDC2-u_f/IFDC2-rev. The flanking regions were amplified using the primer pairs *recA*-fwd/*recA*-IFDC2-u_r and *recA*-IFDC2-d_f/*recA*-rev with UA159 wildtype as the DNA template. The individual amplicons were mixed and assembled via OE-PCR with the primer pair *recA*-fwd/*recA*-rev. The OE-PCR amplicon was transformed and selected on agar plates supplemented with 12.5 μg ml^{-1} erythromycin to generate the strains recKO-IFD2 (and the corresponding LRF version). To remove the IFDC2 cassette and generate a markerless *recA* deletion mutant, these strains were subsequently transformed with a second OE-PCR amplicon created by first PCR amplifying two DNA fragments flanking the *recA* ORF using the primers *recA*-fwd/*recA*ml-rev and *recA*ml-fwd/*recA*-rev. The two amplicons were mixed and assembled via OE-PCR using the primer pair *recA*-fwd/*recA*-rev. Transformants were selected on agar plates supplemented with 0.4% (wt/vol) *p*-Cl-Phe (Sigma) to create the strains recKO (and the corresponding LRF version). To create *irvA* and *recA* double mutant strain ArecKO, *irvA* was deleted as previously described (Liu et al., 2015) from parental strain recKO. To create the complemented *recA* mutant recComp, the *recA* ORF was inserted immediately downstream of the constitutively expressed *gyrA* ORF. The upstream *gyrA* flanking region was PCR amplified using the primers *gyrA-recA*_F1/ *gyrA-recA*_R1 and UA159 gDNA as a DNA template. The *recA* ORF + Shine-Dalgarno sequence (51bp upstream of start codon) was amplified using the primers *gyrA-recA*_F2/ *gyrA-recA*_R2. The spectinomycin resistance cassette was amplified with the primer pair *gyrA-recA*_F3/*gyrA-recA*_R3 and plasmid pDL278 (Chen and LeBlanc, 1992) as a DNA template. The downstream fragment was amplified with the primer pair *gyrA-recA*_F4/ *gyrA-recA*_R4. The amplicons were mixed and assembled using OE-PCR PCR with the primer pair *gyrA-recA*_F1/*gyrA-recA*_R4. The OE-PCR amplicon was subsequently transformed into strain UA159 (and the corresponding and LRF version) and selected on agar plates supplemented with 850 μg ml^{-1} spectinomycin. The markerless *recA* deletion was then engineered into this strain as described above to create the complemented *recA* mutant strain recComp (and LRFrecComp).

Construction of double FLAG tagged IrvR for western blot
Firstly, to insert an internal FLAG tag for the detection of the NTD autocleavage fragment, the FLAG ORF was inserted into the *irvR* ORF 3 codons upstream of the ClpXP degron sequence. The upstream homologous fragment was amplified from UA159 with the primer pair *irvR*-fwd3/Int-*irvR*-FLAG-up_r1. The downstream homologous fragment containing the FLAG ORF was amplified from UA159 with the primer pair Int-*irvR*-FLAG-f1/Int-*irvR*-FLAG-f2 & *SMU1400c*-fwd. These two PCR amplicons were mixed and assembled by OE-PCR with the primer pair *irvR*-fwd3/*SMU1400*c-fwd. The OE-PCR amplicon was transformed into strain ARKO-IFD2 and selected on agar plates containing 0.4% (wt/vol) *p*-Cl-Phe plates to generate the strain RF-INT. To construct the RF-INT derivatives (RF-INTS224A, RF-INTK260A, RF-INTVRR or RF-INTK260VRR), strains RS224A, RK260A, RVRR, or RK260AVRR were used as separate templates to amplify the respective downstream homologous fragments.

Secondly, an intermediate strain was constructed that contains both internal and C-terminal FLAG tags on IrvR and is expressed from the native *irvR* promoter. The *irvR* locus was amplified from strain RF-INT and tagged with the 3x FLAG ORF on the *irvR* 3' with two successive PCR reactions using the primer pairs *irvR*-fwd3 & *irvR*-FLAG-r1/*irvR*-FLAG-r2. The kanamycin resistance cassette was amplified with the primer pair *irvR*+KanR-FLAG_dn-f/kanR-rev, while the downstream fragment was amplified with the primer pair *irvR*+kanR_dn-f/SMU1400c-fwd. These PCR amplicons were mixed and assembled via OE-PCR with the primer pair *irvR*-fwd3/SMU1400c-fwd and transformed into UA159 to create the double FLAG tagged strain RFF. To construct

RFF derivatives (RFFS224A, RFFK260A, RFFVRR, or RFFK260AVRR), RF-INT derivative strains (RF-INTS224A, RF-INTK260A, RF-INTVRR, or RF-INTK260VRR) were used as separate templates to amplify the respective downstream homologous fragments.

To construct a constitutively expressed doubly FLAG tagged IrvR (both internal and C-terminal FLAG tag) with an HA epitope-tagged lactate dehydrogenase (Ldh), we inserted the HA epitope ORF before the *ldh* stop codon along with the doubly FLAG-tagged *irvR* ORF immediately downstream of the *ldh* ORF. The upstream flanking region was amplified from UA159 with the primer pair *ldh*-fwd/*ldh*-HA-up_r. The doubly FLAG-tagged *irvR* ORF + kanamycin resistance cassette was amplified from strain RFF with the primer pair HA-*irvR*-fwd/HA-irvR-fwd_2/kanR-rev. The downstream homologous fragment was amplified from UA159 with the primers *ldh*-*irvR*-FLAG-dn/*ldh*-rev. These resulting PCR amplicons were mixed and assembled by OE-PCR with the primer pair *ldh*-fwd/*ldh*-rev. The OE-PCR amplicon was transformed into UA159 and selected on agar plates supplemented with 850 μg ml^{-1} kanamycin to create the strain LhARFF. To construct the LhARFF derivatives (LhARFFS224A, LhARFFK260A, LhARFFVRR, or LhARFFK260AVRR), RFF derivative strains (RFFS224A, RFFK260A, RFFVRR or RFFK260VRR) were used as separate templates to amplify the respective downstream homologous fragments.

Creation of IrvR autocleavage deficient mutants

To construct *irvR* point mutations, we first replaced the adjacent *irvR/A* genes with the IFDC2 cassette and then markerlessly knocked-in both *irvA* and a mutagenized *irvR* to replace the IFDC2 cassette. The IFDC2 cassette was amplified from plasmid pIFDC2 (Xie et al., 2011) using the primer pair IFDC2-u_f/IFDC2-rev. The *irvR/A* flanking regions were amplified using the primer pairs *irvA*-rev1/ *irvAR*ml-rev and *irvAR*ml-fwd/*SMU1400*c-fwd with UA159 gDNA as a DNA template. The resulting amplicons were mixed and assembled via OE-PCR with the primers *irvA*-rev1/*SMU1400*c-fwd. The OE-PCR amplicon was transformed into strain UA159 and selected with 12.5 μg ml^{-1} erythromycin to generate the strain ARKO-IFD2. To create the S224A IrvR mutation, the upstream and downstream homologous fragments were amplified with the primer pairs *irvA*-rev1/ *irvR* S224A-R and *irvR* S224A-F/*SMU1400*c-fwd. To create the K260A IrvR mutation, the upstream and downstream fragments were amplified with primer pairs *irvA*-rev1/*irvR* K260A-R and *irvR* S224A-F/*SMU1400*c-fwd. To create the VRR IrvR mutations, the upstream and downstream fragments were amplified with the primer pairs *irvA*-rev1/*irvR* VRR-R and *irvR* VRR-F/*SMU1400*c-fwd. Each of these amplicon pairs were mixed and assembled via OE-PCR with the primers *irvA*-rev1/*SMU1400*c-fwd. The resulting OE-PCR amplicons were transformed into strain ARKO-IFD2 and selected on agar plates supplemented with 0.4% (wt/vol) *p*-Cl-Phe to generate the markerless point mutant strains RS224A, RK260A, and RVRR. For the K260/VRR double mutant, the downstream fragment was amplified from strain RK260A with the primer pair *irvR* VRR-F/*SMU1400*c-fwd. To create autocleavage deficient point mutant versions of strain LRF, the same assembly protocol was performed as LRF except that strains RS224A, RK260A, RVRR, or RK260AVRR were used as separate templates to amplify the respective downstream homologous fragments.

Construction of luciferase reporters

The homologous flanking regions of *irvA* were amplified using the primer pairs *irvA*-fwd1/*irvA_renG*_up-r and *irvA_renG*_dn-f/*irvA*-rev1 with UA159 gDNA as a DNA template. The green renilla ORF (*renG*) was amplified using the primer pair RenG-F/*renG*_Spc_dn-r and gDNA from strain *ldh-renG* (Merritt et al., 2016) as a DNA template. The spectinomycin resistance cassette was amplified from plasmid pDL278 with the primer pair pDL278-fwd/ pDL278-rev. The resulting four PCR amplicons were mixed and assembled via OE-PCR using the primer pair *irvA*-fwd1/ *irvA*-rev1. The OE-PCR amplicon was transformed into UA159 and selected on agar plates supplemented with 850 μg ml^{-1} spectinomycin to create the strain ArenG.

Construction of strains used for coimmunoprecipitation

S. mutans was engineered to express two copies of *irvR*, one encoding an N-terminal FLAG tag (F-R) and the other encoding an N-terminal HA tag (H-R). Firstly, an N-terminal FLAG tagged IrvR was engineered for expression via the *ldh* locus. The upstream region was amplified with two successive PCR reactions using the

primer pairs *ldh*-fwd and *ldh*-rbs-flag-r1/*ldh*-rbs-flag-r2 using UA159 gDNA as a template. The downstream fragment containing the epitope tagged *irvR* ORF, kanamycin resistance cassette, and downstream homologous region was amplified from strain LRF in two successive PCR reactions with the primer pairs FLAG-*irvR*-f1/ FLAG-*irvR*-f2 and *ldh*-rev. These two final PCR amplicons were mixed and assembled via OE-PCR with the primer pair FLAG-*irvR*-down-f/*ldh*-rev. The OE-PCR amplicon was transformed into UA159 and selected on agar plates supplemented with kanamycin. To create the strain LFRFK260A, the same procedure was employed using a downstream fragment amplified from strain RFK260A in two successive PCR reactions using the primer pairs *ldh*-fwd and *ldh*-rbs-flag-r1/*ldh*-rbs-flag-r2. Once these strains were constructed, the next step was to markerlessly insert an ORF encoding an N-terminal HA tagged IrvR immediately downstream of the constitutively expressed *gapC* gene. The IFDC2 cassette was amplified from plasmid pIFDC2 (Xie et al., 2011) using the primer pair IFDC2-u_f/IFDC2-rev. The *gapC* flanking regions were amplified from UA159 using the primer pairs *gapC*-fwd/*gapC*-IFDC2-rev and *IFDC2-down*-f/*gapC*-rev. The resulting amplicons were mixed and assembled via OE-PCR with the primers *gapC*-fwd/*gapC*-rev. The OE-PCR amplicon was transformed into LFRFK260A and selected on agar plates supplemented with 12.5 μg ml^{-1} erythromycin. To replace the IFDC2 cassette with DNA encoding an N-terminal HA tagged IrvR, the upstream flanking region was amplified in two successive PCR reactions using the primer pairs *gapC*-fwd and *gapC*+RBS_dn-r1/RBS-HA_dn-R2, the *irvR* K260A ORF was amplified with the primer pair HA-*irvR*-f/*irvR*-down-r, and the downstream flanking region was amplified using the primer pair *irvR*-down-r/*gapC*-rev. The resulting amplicons were mixed and assembled via OE-PCR using the primers *gapC*-fwd/*gapC*-rev. The OE-PCR amplicon was transformed into the IFDC2 intermediate strains and selected on agar plates supplemented with 0.4% (wt/vol) *p*-Cl-Phe. Lastly, the native copy of *irvR* was deleted as previously described to generate the strain F-R/H-R*.* To create the IrvR truncation mutants tested via coimmunoprecipitation, a direct repeat-mediated markerless mutagenesis (DR-CIMM) approach was employed as previously described (Zhang et al., 2017). For the deletion of amino acids 66-122 from the FLAG tagged IrvR in strain F-R/H-R, the upstream and downstream flanking regions were amplified from strain F-R/H-R with the primer pairs *ldh*_N_*irvR*-3-F1/*ldh*_N_*irvR*-3-R1 and *ldh*_N_*irvR*-3-F5/*ldh*_N_*irvR*-3-R5. The IFDC3 counterselection cassette was amplified from strain T260S (Zhang et al., 2017) with the primer pair IFDC2-u_f/IFDC2-rev, while a fragment of *irvR* from the 66th codon was amplified from UA159 with the primer pair *ldh*_N_*irvR*-3-F3/*ldh*_N_*irvR*-3-R3. The direct repeat sequence used for DR-CIMM was amplified from UA159 with the primer pair *ldh*_N_*irvR*-3-F4/*ldh*_N_*irvR*-3-R4. These two PCR amplicons were assembled by overlap extension PCR with primer pair *ldh*_N_*irvR*-3-F1/*ldh*_N_*irvR*-3-R5 and transformed in *ldh*-FLAG-*irvR* K260A & *gapC*-HA-irvR K260A and selected with erythromycin. Transformants were cultured without selection and then plated on agar plates supplemented with 0.4% (wt/vol) *p*-Cl-Phe to isolate the markerless *irvR* truncation mutants. The same approach was used to create the 66-80, 81-94, 95-108, and 109-122 IrvR truncation mutants using the respective primers listed in Table S2.

Site-directed mutagenesis of recA

To create the constitutively activated RecA mutant, the IFDC2 cassette was inserted immediately downstream of *recA* on the chromosome and then a mutagenized copy of *recA* was used to markerlessly replace both IFDC2 and the wild-type copy of *recA*. For the RecA P80D mutant, the homologous flanking regions were amplified from UA159 using the primer pairs *recA*-fwd/*recA*-P80D-rev and *recA*-P80D-fwd/*recA*-rev. For the RecA D171R mutant, the flanking regions were amplified from UA159 using the primer pairs *recA*-fwd/*recA*-D171R-rev and *recA*-D171R-fwd/*recA*-rev. For the RecA P80D/D171R double mutant, three homologous fragments were amplified using the primer pairs *recA*-fwd/*recA*-P80D-rev, *recA*-P80D-fwd/*recA*-D171R-rev and *recA*- D171R-fwd/*recA*-rev. For each of the *recA* mutant constructs, individual PCR amplicons were mixed and assembled via OE-PCR with the primers *recA*-fwd/*recA*-rev. The resulting OE-PCR amplicons were transformed into the previously described strain recKO-IFD2 and selected on agar plates supplemented with 0.4% (wt/vol) *p*-Cl-Phe to create the strains recP80D, recD171R, and recA#.

Construction of gbpC-gfp *transcription fusion reporter*

To construct the *gbpC-gfp* transcription fusion reporter strain, the *gfp* ORF was markerlessly inserted immediately downstream of the stop codon of *gbpC*. The upstream *gbpC* flanking homologous region was

amplified from UA159 with the primer pair *gbpC-gfp*_F1/ *gbpC-gfp*_R1. The IFDC2 cassette was amplified from plasmid pIFDC2 (Xie et al., 2011) using the primer pair IFDC2-u_f/IFDC2-rev, while the downstream flanking homologous fragment was amplified from UA159 with the primer pair *gbpC-gfp*_F3/ *gbpC-gfp*_R3. These three PCR amplicons were mixed and assembled via OE-PCR with the primer pair gbpC-gfp_F3/ gbpC-gfp_R3 and then transformed into UA159 with selection on agar plates supplemented with 12.5 μg ml^{-1} erythromycin. Next, the upstream *gbpC* homologous region was amplified from UA159 with the primer pair *gbpC-gfp*_F1/ *gbpC-gfp*_R4, the *gfp* ORF was amplified from strain Ndeg-gfp (Liu et al., 2017) with the primer pair sp_*gfp*_F/*gfp*_R, and the downstream homologous fragment was amplified from UA159 with the primer pair *gbpC-gfp*_F5/ *gbpC-gfp*_R3. These three PCR amplicons were mixed and assembled via OE-PCR with the primer pair *gbpC-gfp*_F3/ *gbpC-gfp*_R3. The OE-PCR amplicon was transformed into the IFDC2 intermediate strain and selected on plates supplemented with 0.4% (wt/vol) *p*-Cl-Phe to create the *gbpC-gfp* reporter strain CGFP.

Luciferase Assays

Luciferase reporter assays with the green renilla luciferase were performed as previously described (Merritt et al., 2016). Overnight cultures of *S. mutans* reporter strains were diluted 1:25 in THYE or BTR-G medium and incubated anaerobically at 37 °C until reaching mid-log growth phase. 100 μl of culture was mixed with 1 μl (0.75 mg ml^{-1}) coelenterazine-h (NanoLight Technologies). Bioluminescence was measured with a GloMax plate reader (Promega) using the Luminescence Quick Read Program and an integration time of 0.5 sec. Luminescence values were normalized by dividing sample RLU values by culture optical density OD_{600} values.

Supplementary References

Chen, Y.Y., and LeBlanc, D.J. (1992). Genetic analysis of scrA and scrB from Streptococcus sobrinus 6715. Infect Immun *60*, 3739-3746.
Liu, N., Chaudhry, M.T., Xie, Z., Kreth, J., and Merritt, J. (2017). Identification of New Degrons in Streptococcus mutans Reveals a Novel Strategy for Engineering Targeted, Controllable Proteolysis. Front Microbiol *8*, 2572.
Liu, N., Niu, G., Xie, Z., Chen, Z., Itzek, A., Kreth, J., Gillaspy, A., Zeng, L., Burne, R., Qi, F.*, et al.* (2015). The Streptococcus mutans irvA gene encodes a trans-acting riboregulatory mRNA. Mol Cell *57*, 179-190.
Merritt, J., Senpuku, H., and Kreth, J. (2016). Let there be bioluminescence: development of a biophotonic imaging platform for in situ analyses of oral biofilms in animal models. Environ Microbiol *18*, 174-190.
Xie, Z., Okinaga, T., Qi, F., Zhang, Z., and Merritt, J. (2011). Cloning-independent and counterselectable markerless mutagenesis system in Streptococcus mutans. Appl Environ Microbiol *77*, 8025-8033.
Zhang, S., Zou, Z., Kreth, J., and Merritt, J. (2017). Recombineering in Streptococcus mutans Using Direct Repeat-Mediated Cloning-Independent Markerless Mutagenesis (DR-CIMM). Front Cell Infect Microbiol *7*, 202.

Original blots presented in the manuscript

Figure 1C

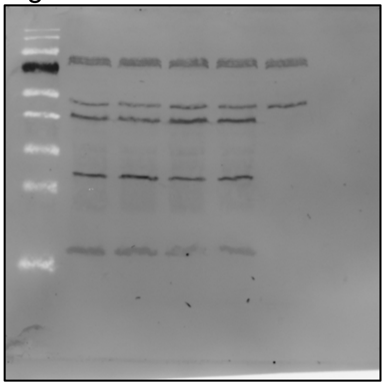

IrvR Autocleavage

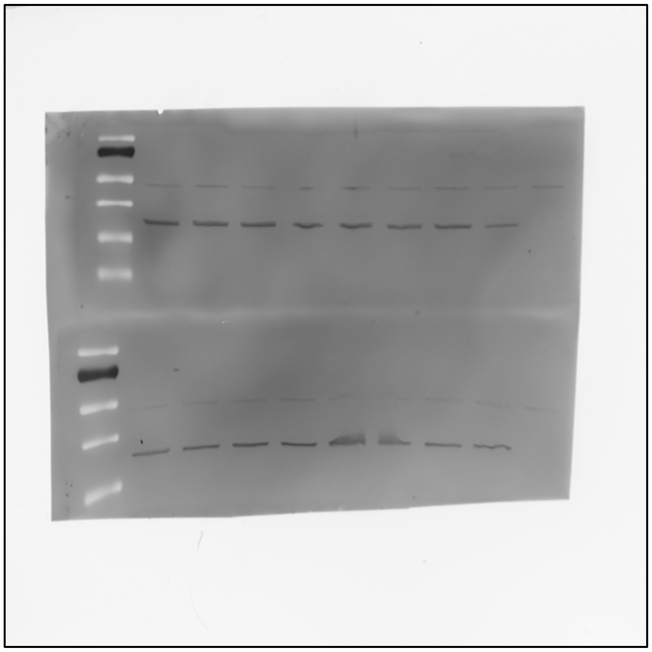

Loading control (Top, Lanes #2–5)

Figure 1D

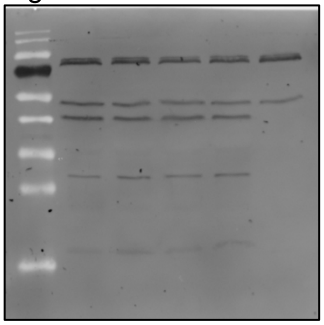

IrvR Autocleavage

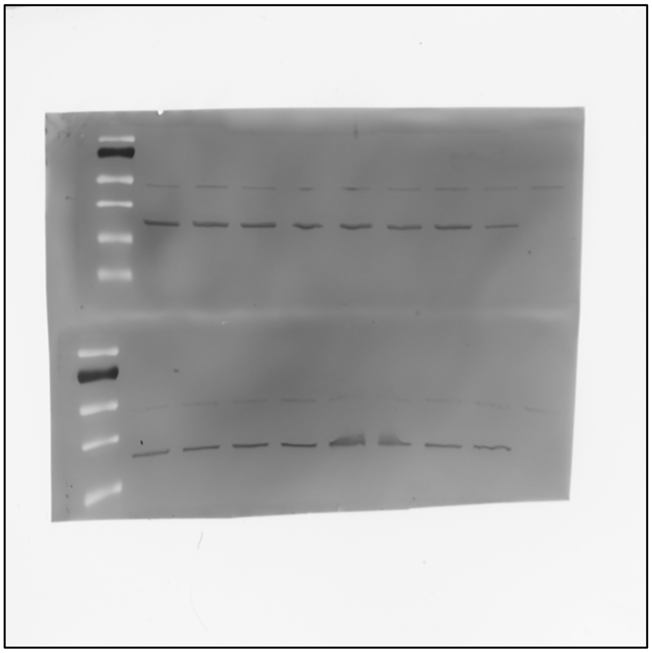

Loading control (Bottom, Lanes #2–5)

Figure 2D

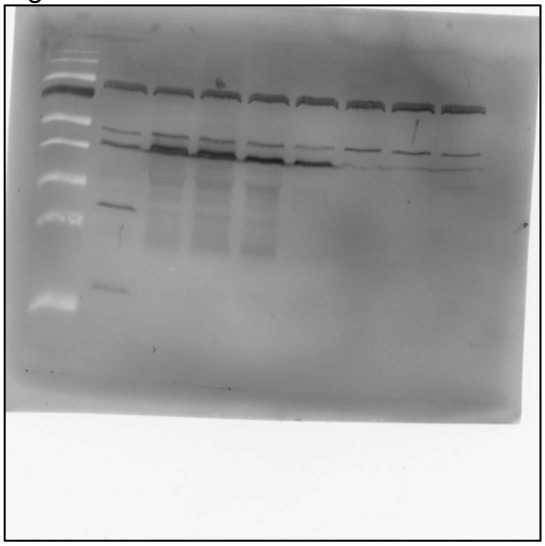

IrvR Autocleavage (Lanes #2–6)

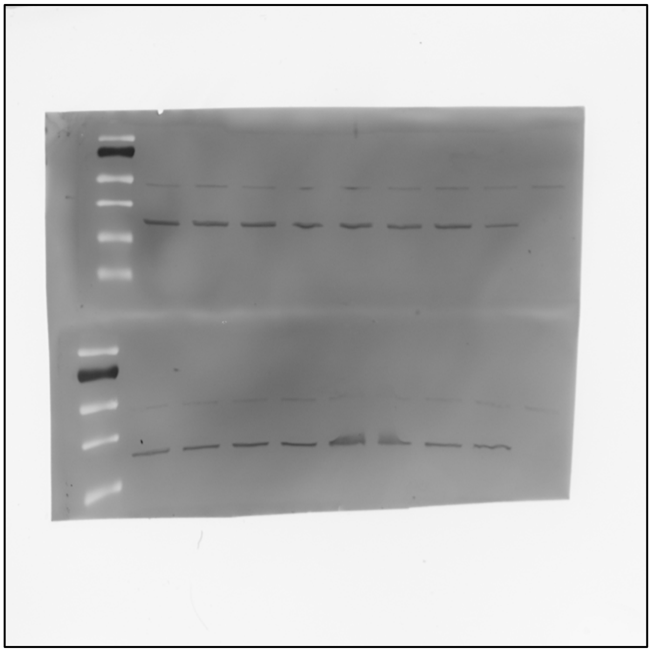

Loading control (Top, Lanes #5–9)

Figure 2E

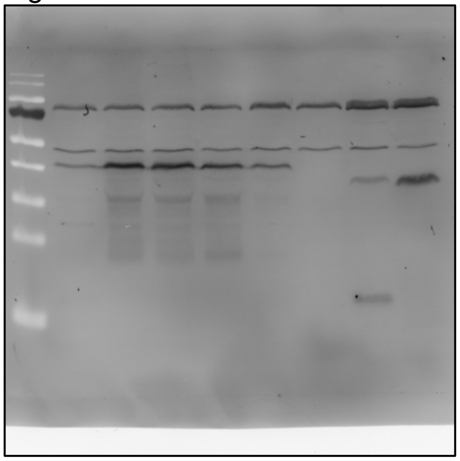

IrvR Autocleavage (Lanes #2–6)

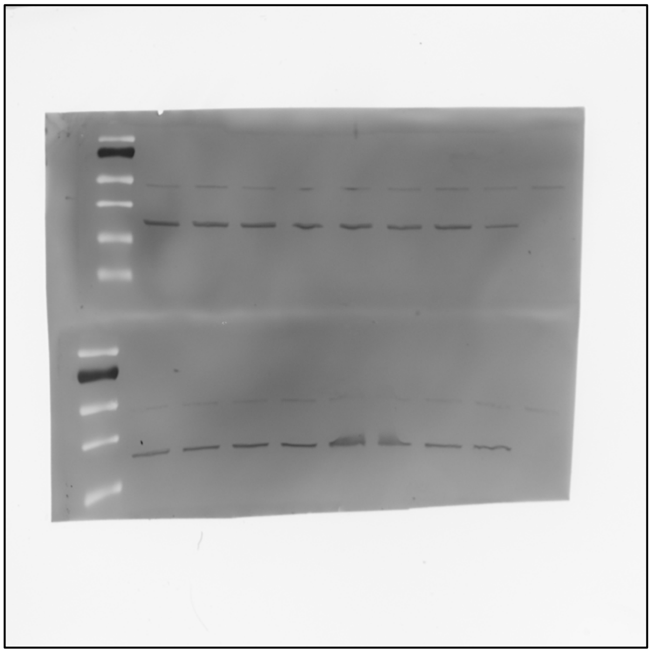

Loading control (Bottom, Lanes #5-9)

Figure 3A

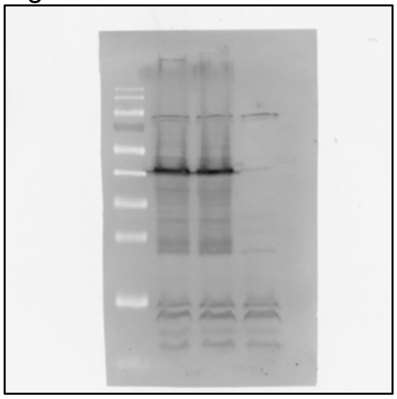

Anti-FLAG input (Lanes #2-4)

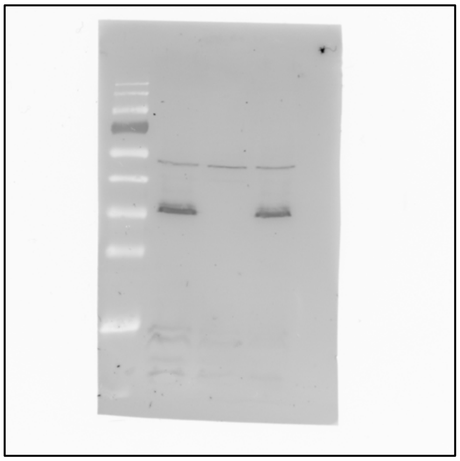

Anti-HA input (Lanes #2-4)

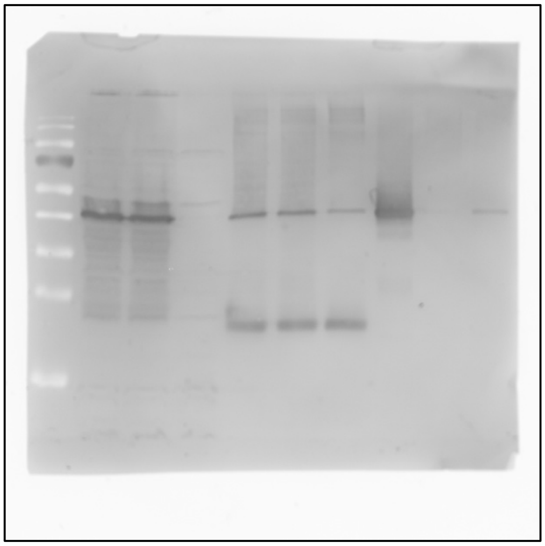

FLAG IP (Lanes #5-6)

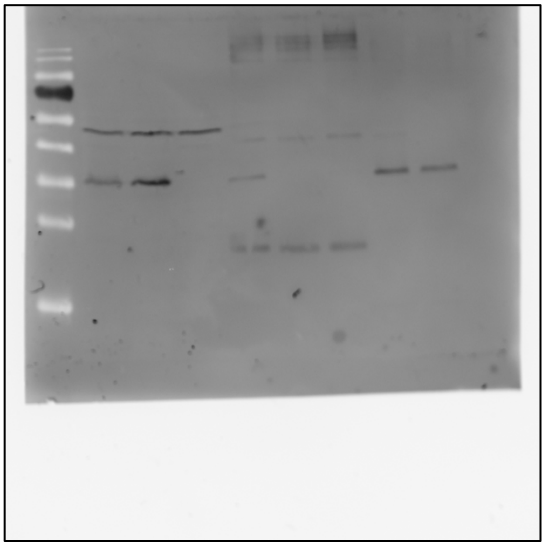

FLAG co-IP Anti-HA western (Lanes #5-6)

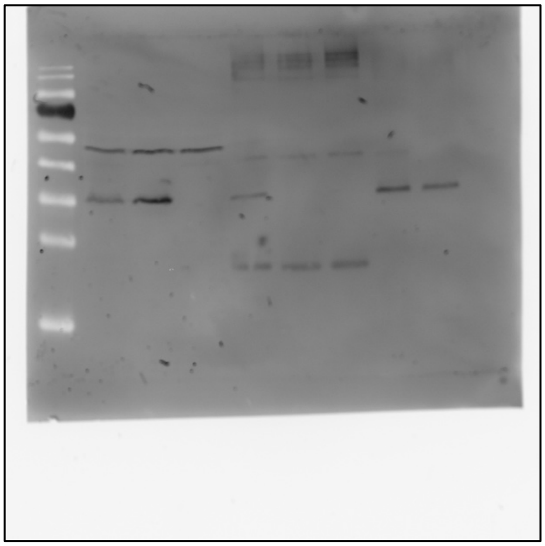

HA IP (Lanes #8-9)

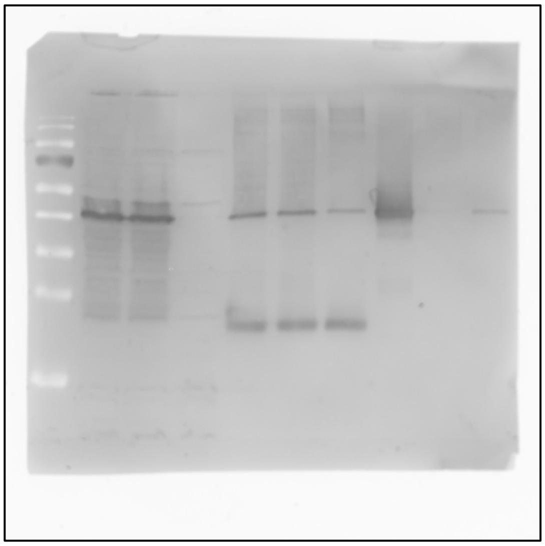

HA co-IP Anti-FLAG western (Lanes #8-9)

Figure 3B

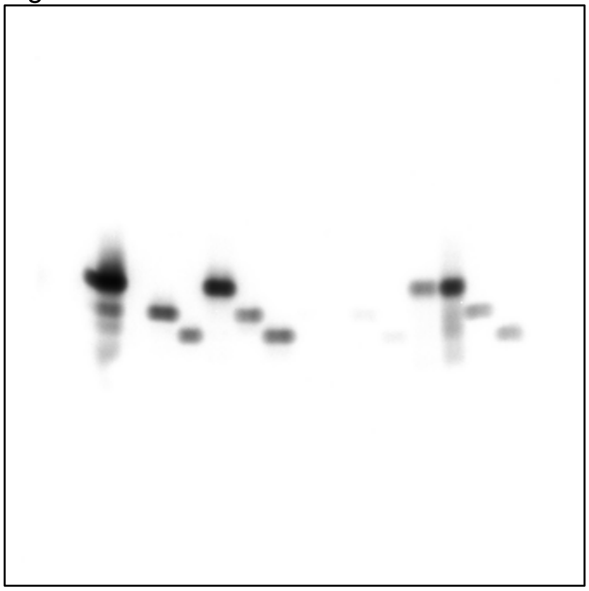

FLAG input western (Lanes #3-4)

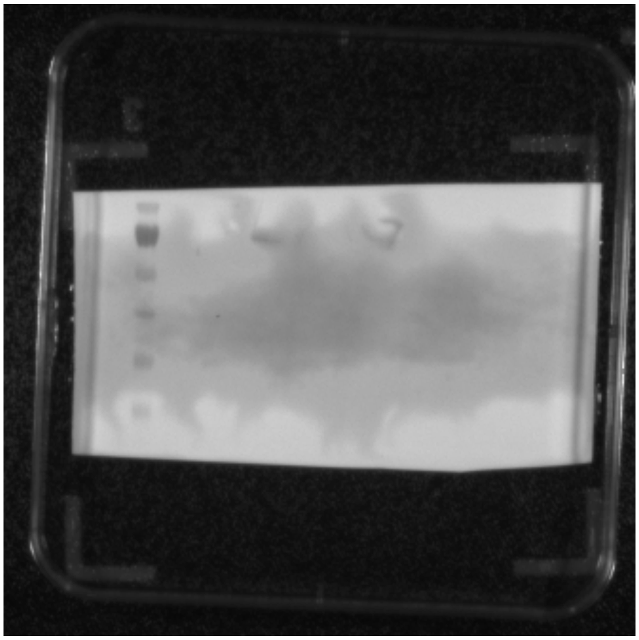

FLAG input (MW marker)

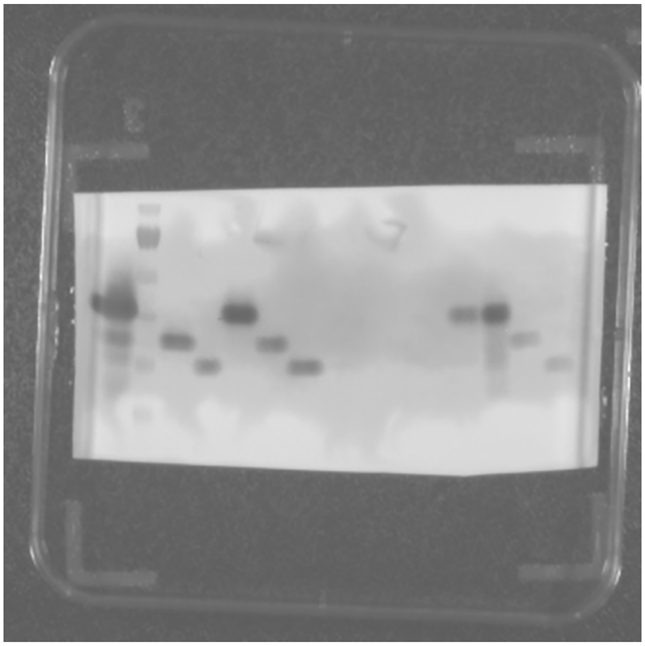

FLAG input (western + MW marker overlay; Lanes #3-4)

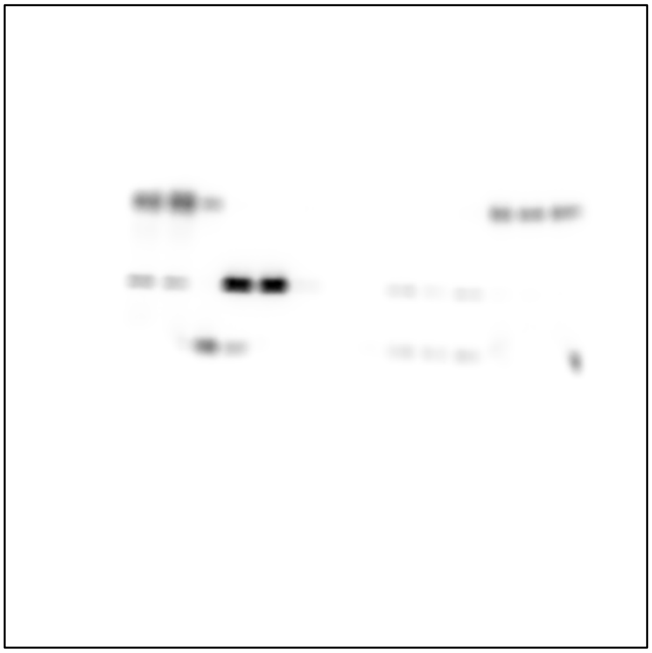

HA input western (Lanes #2-3)

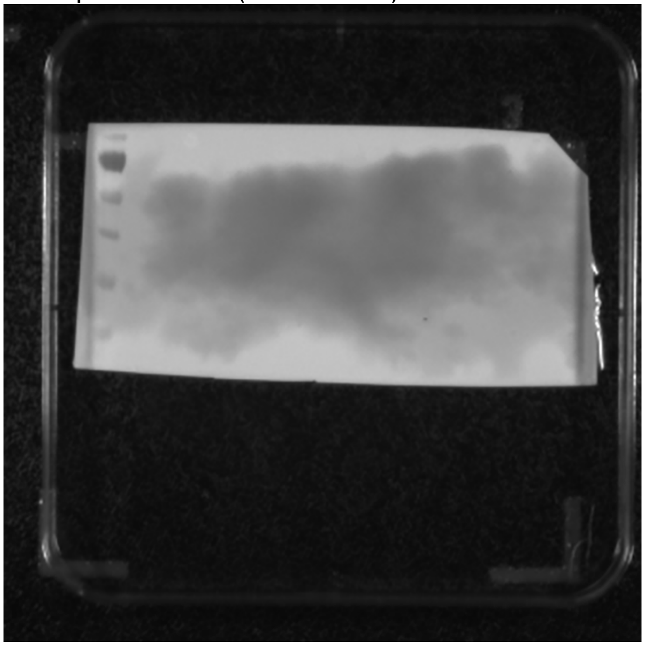

HA input (MW marker)

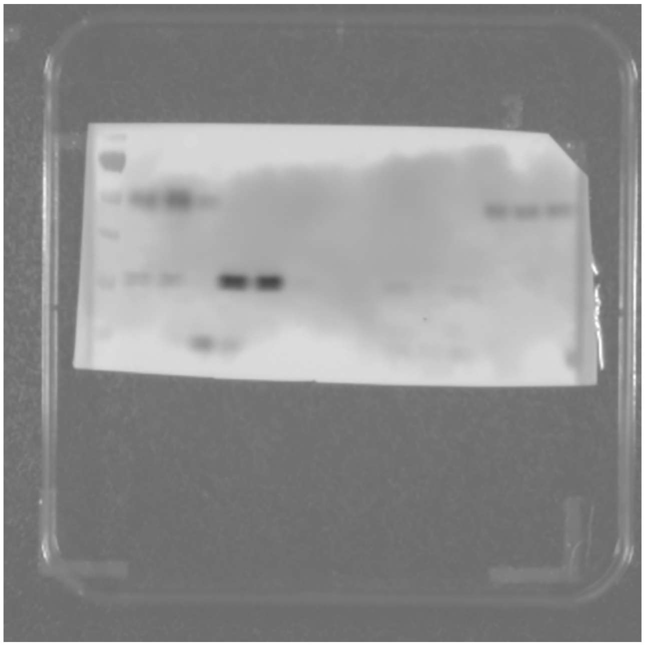

HA input (western + MW marker overlay; Lanes #2-3)

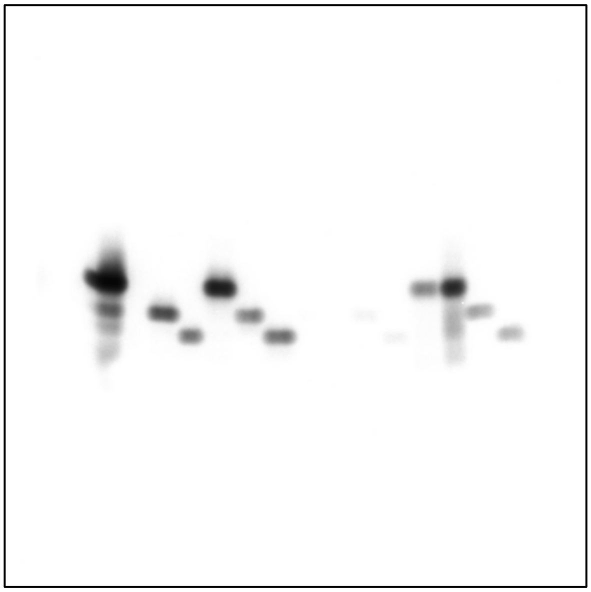

FLAG IP western (Lanes #6-7)

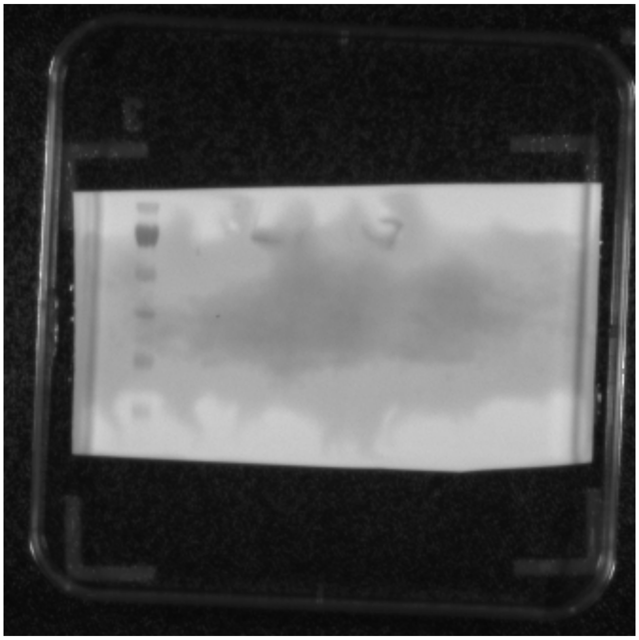

FLAG IP (MW marker)

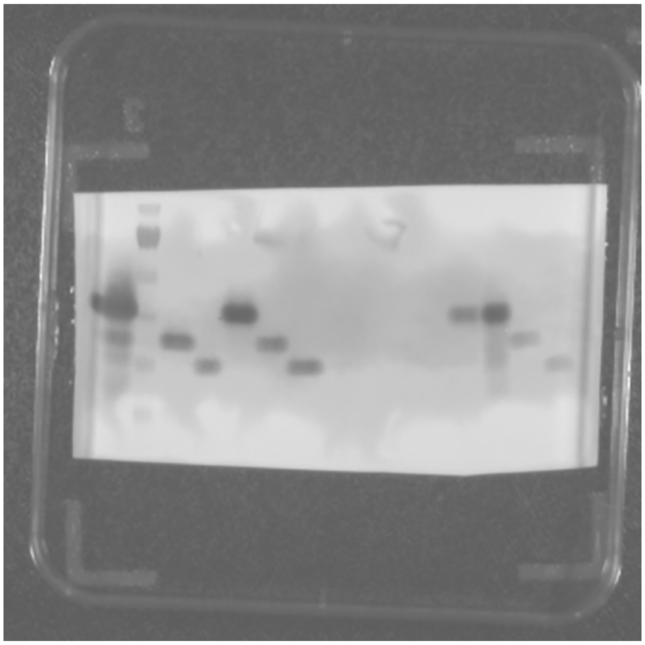

FLAG IP (western + MW marker overlay; Lanes #6-7)

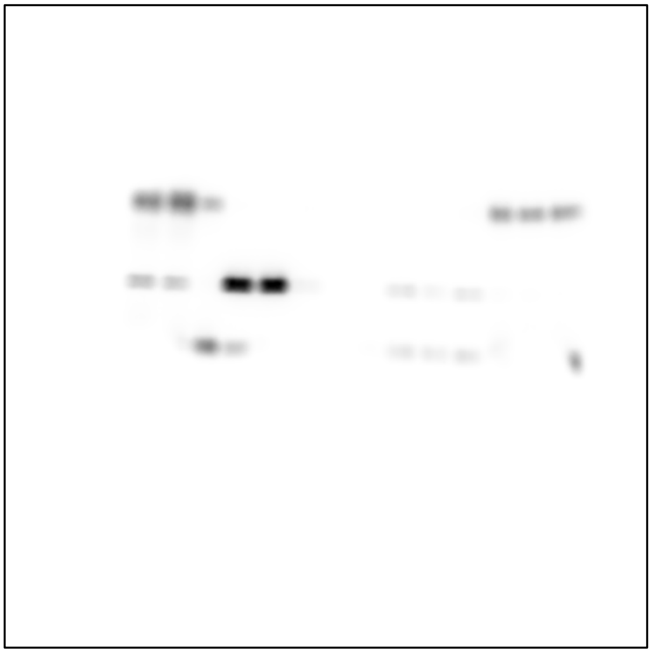

FLAG co-IP Anti HA western (Lanes #6-7)

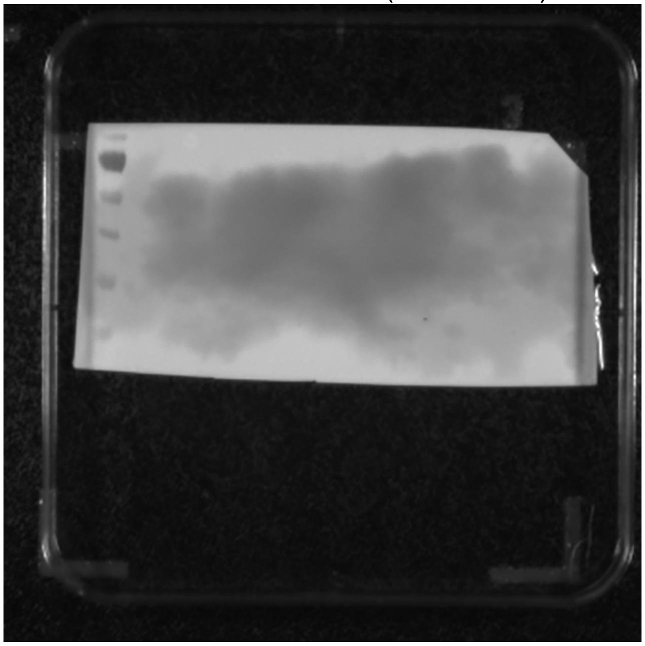

FLAG co-IP (MW marker)

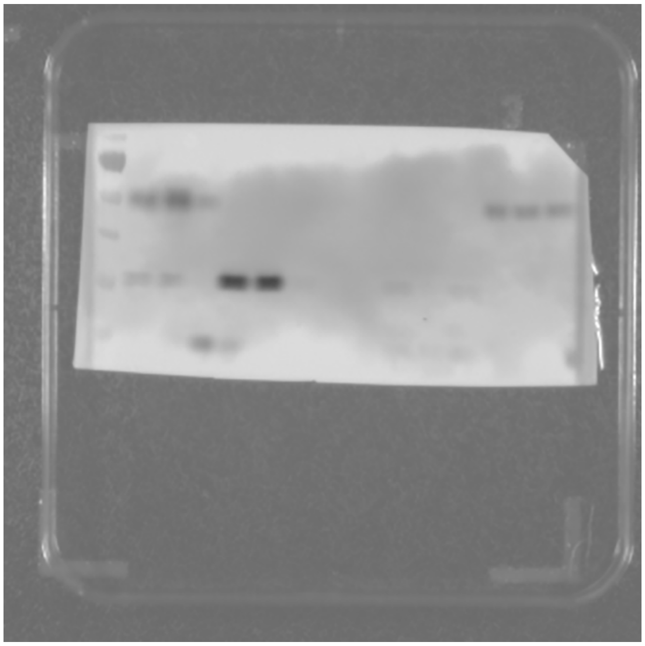

FLAG co-IP (western + MW marker overlay; Lanes #6-7)

Figure 3C

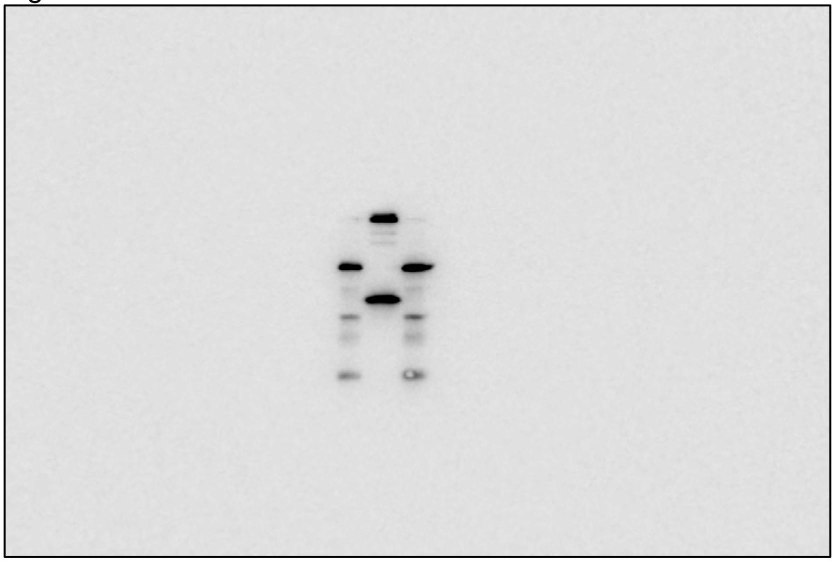

IrvR Autocleavage western (Lanes #2-3)

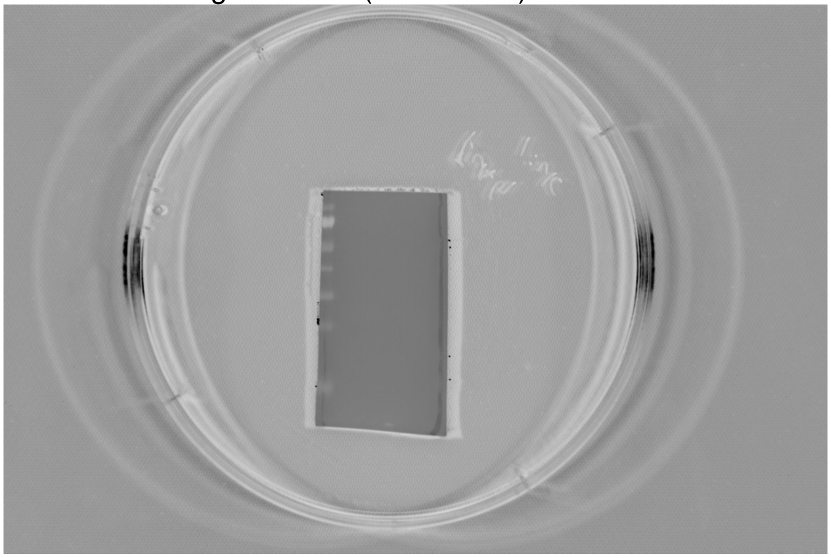

IrvR Autocleavage (MW Marker)

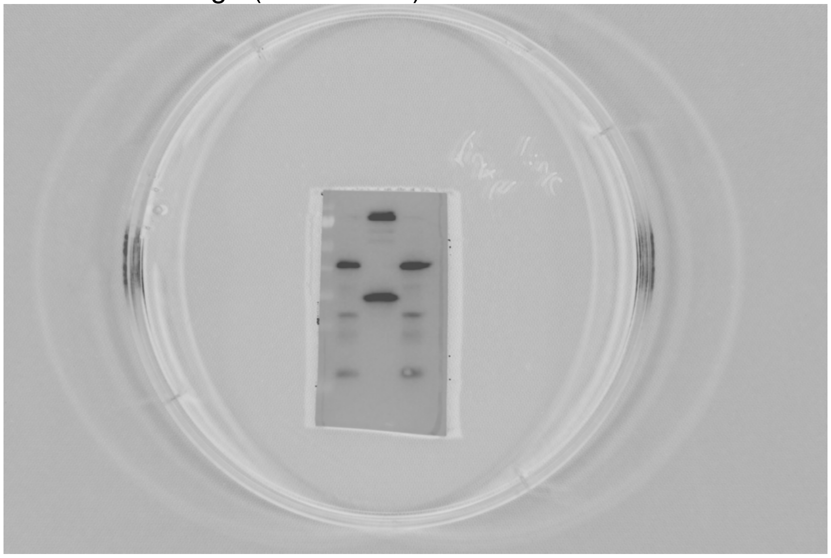

IrvR Autocleavage (western + MW marker overlay; Lanes #2-3)

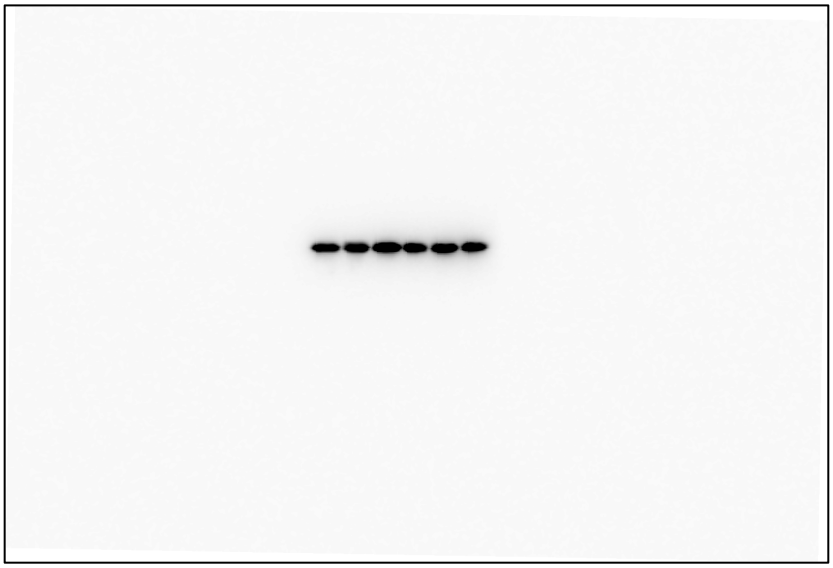

Loading control western (Lanes #3-4)

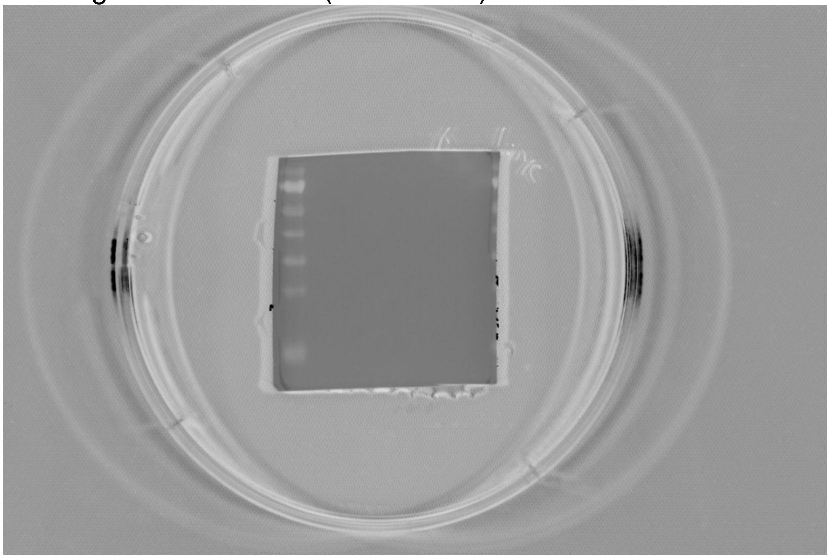

Loading control (MW marker)

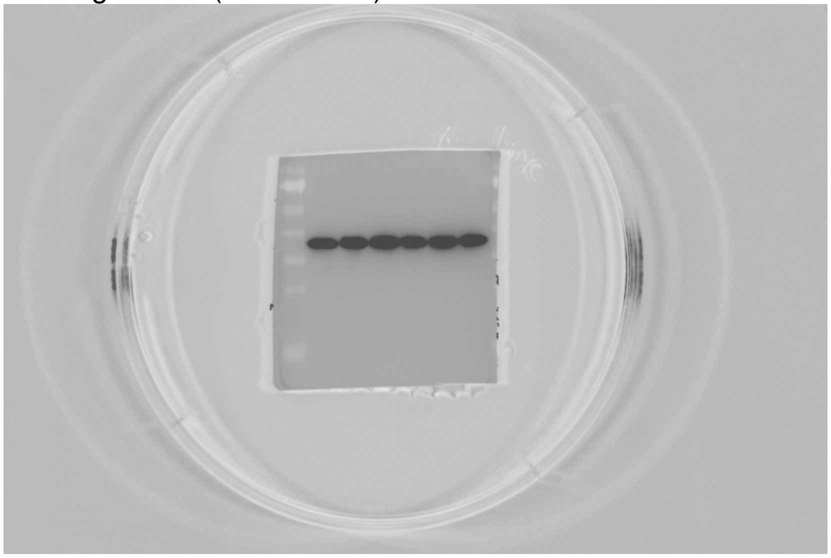

Loading control (western + MW marker overlay; Lanes #3-4)

Figure 3D

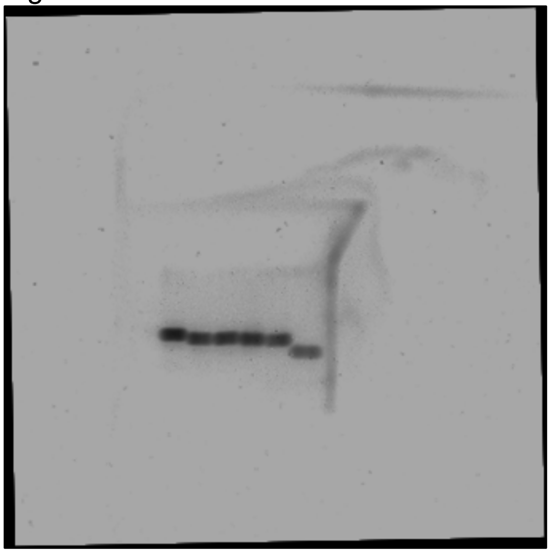

FLAG Input western

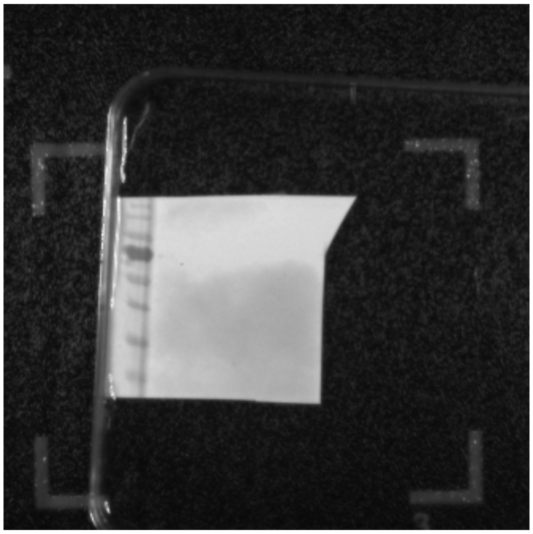

FLAG Input (MW marker)

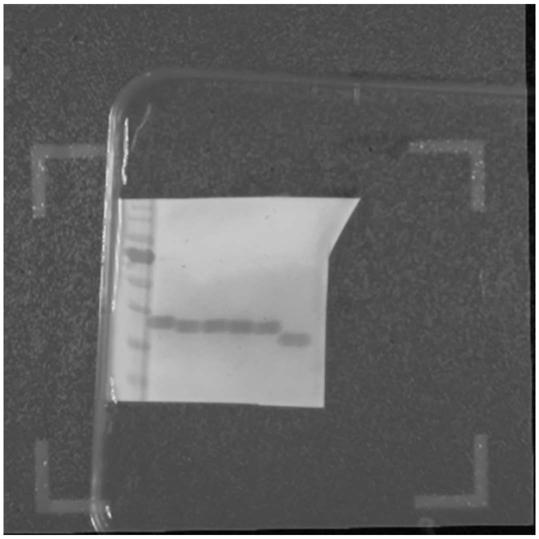

FLAG Input (western + MW marker overlay)

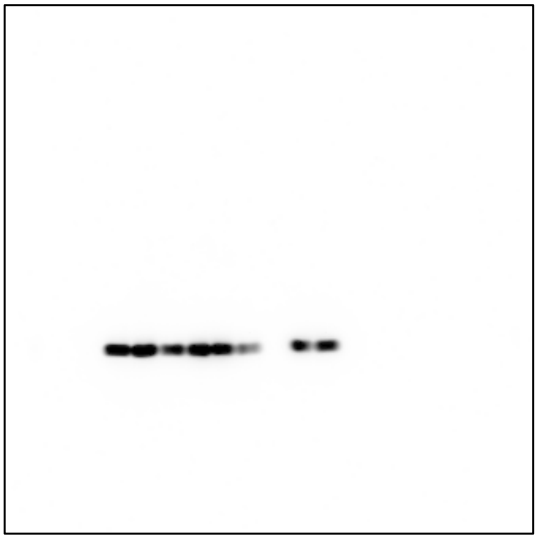

HA Input western (Lanes #2-7)

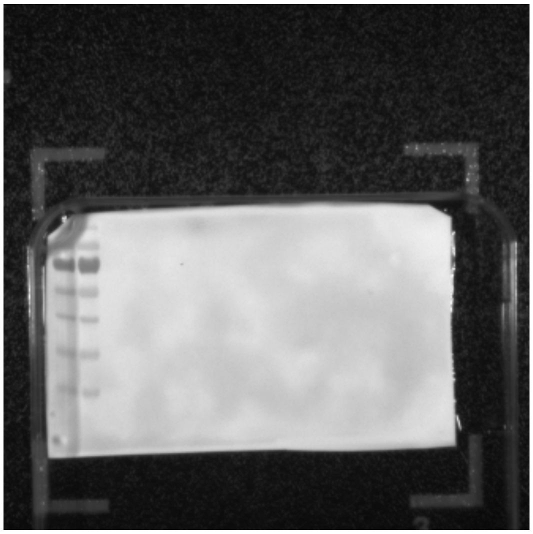

HA Input (MW marker)

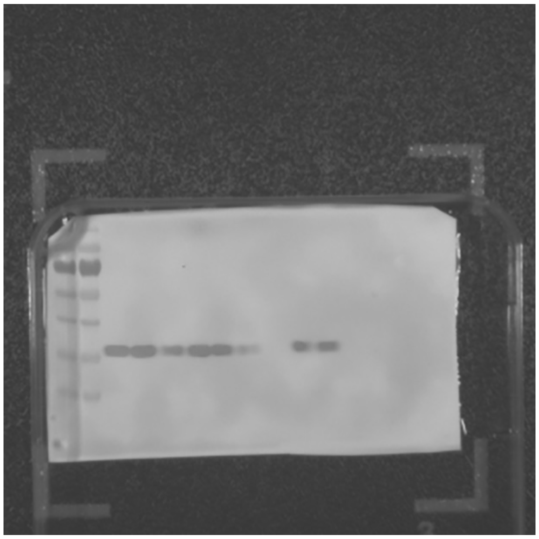

HA Input (western & MW marker overlay; Lanes #2-7)

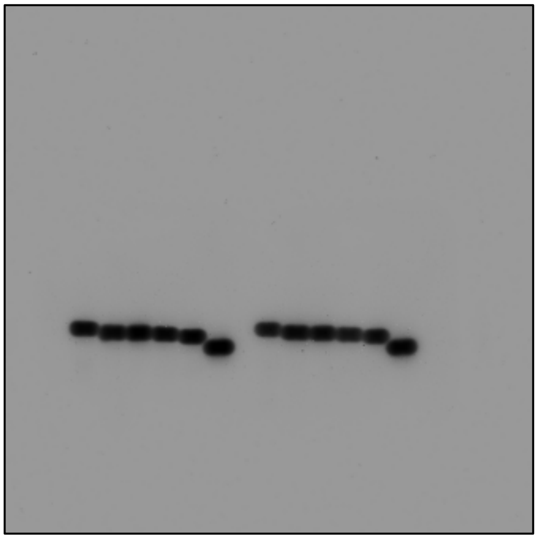
FLAG IP western (Lanes #2-7)

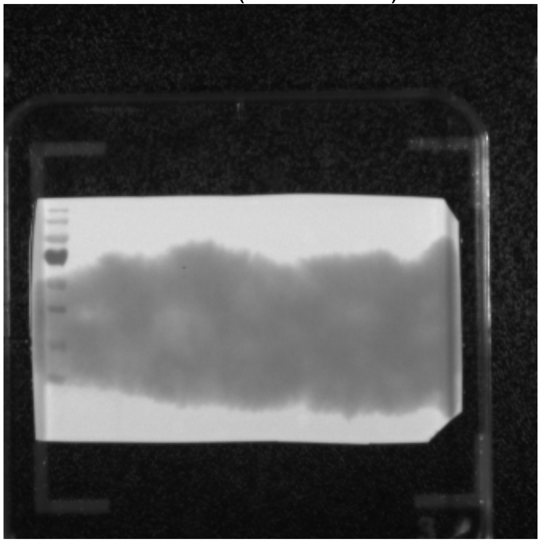
FLAG IP (MW marker)

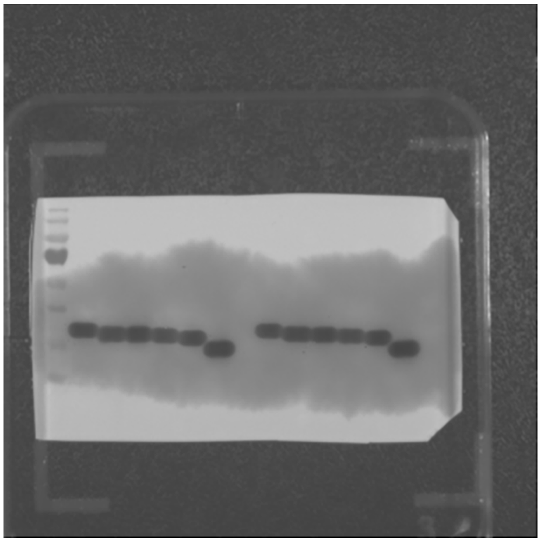
FLAG IP (western + MW marker overlay; Lanes #2-7)

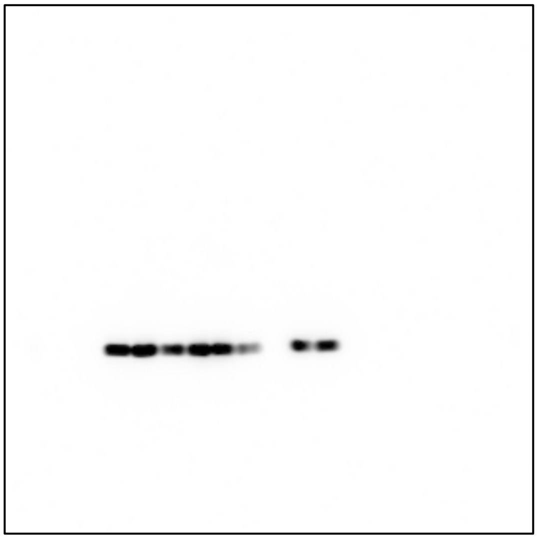

FLAG co-IP Anti-HA western (Lanes #9-14)

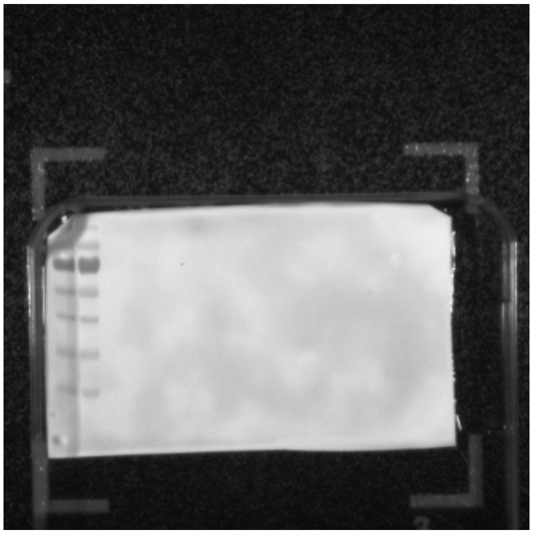

FLAG co-IP (MW marker)

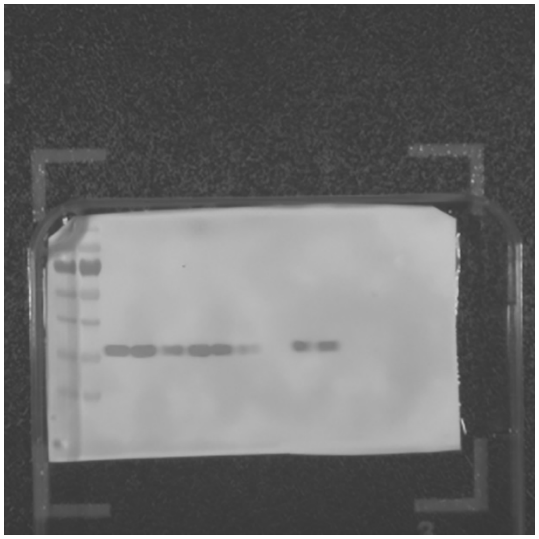

FLAG co-IP (western & MW marker overlay; Lanes #9-14)

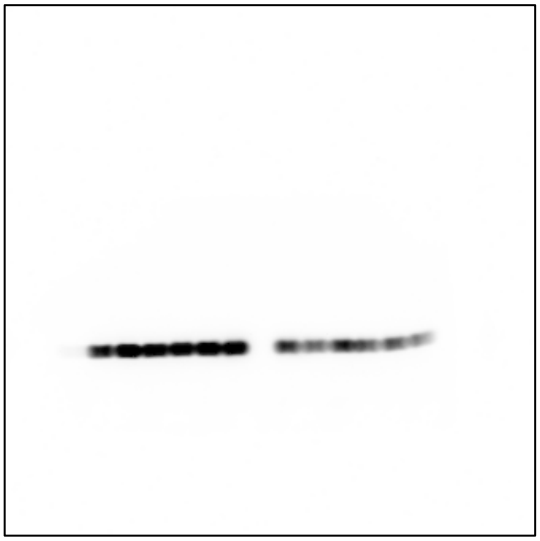
HA IP western (Lanes #2-7)

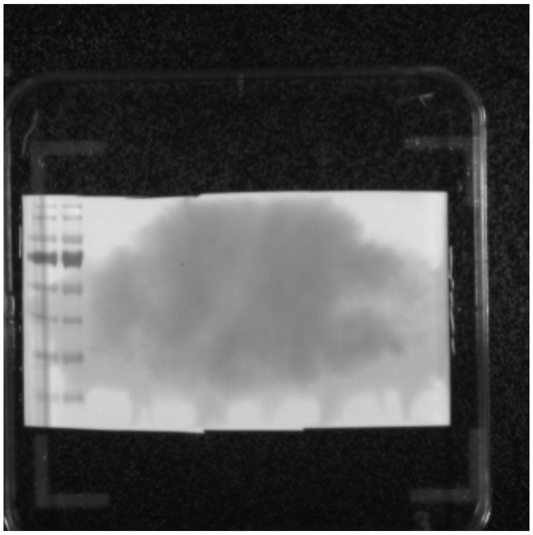
HA IP (MW marker)

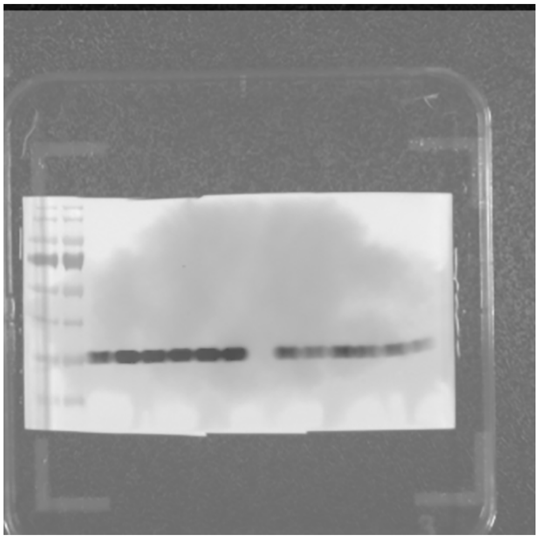
HA IP (western + MW marker overlay; Lanes #2-7)

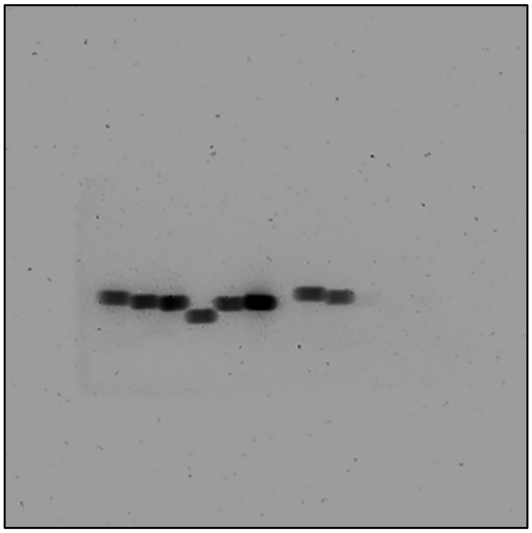

HA co-IP Anti-FLAG western (Lanes #9-14)

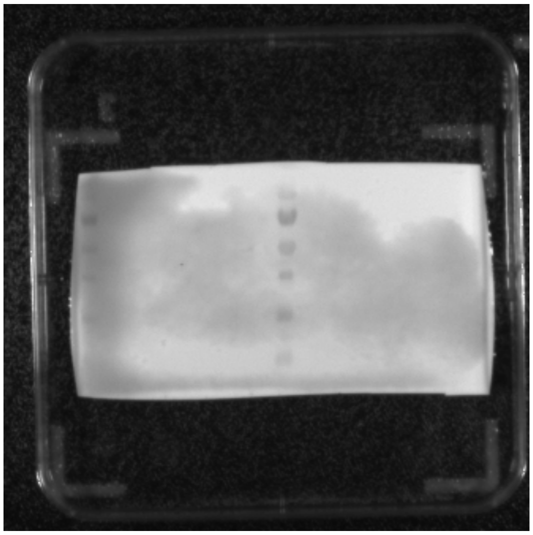

HA co-IP (MW marker)

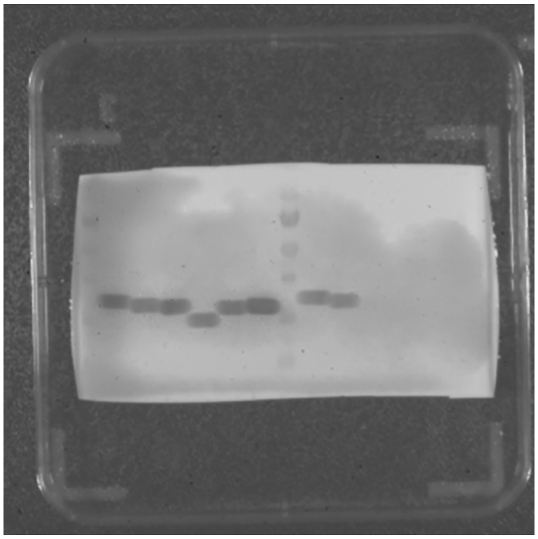

HA co-IP (western + MW marker overlay; Lanes #9-14)

Figure 4F

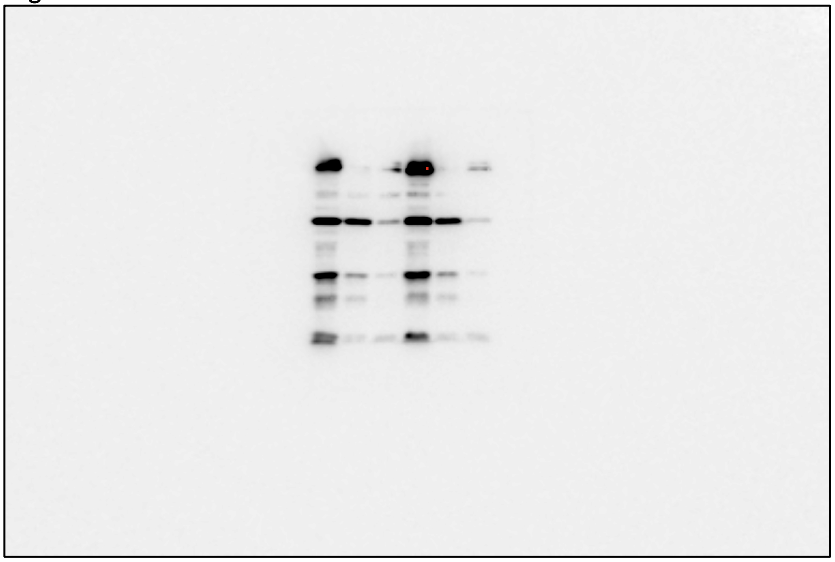

IrvR Autocleavage western (Lanes #5-7)

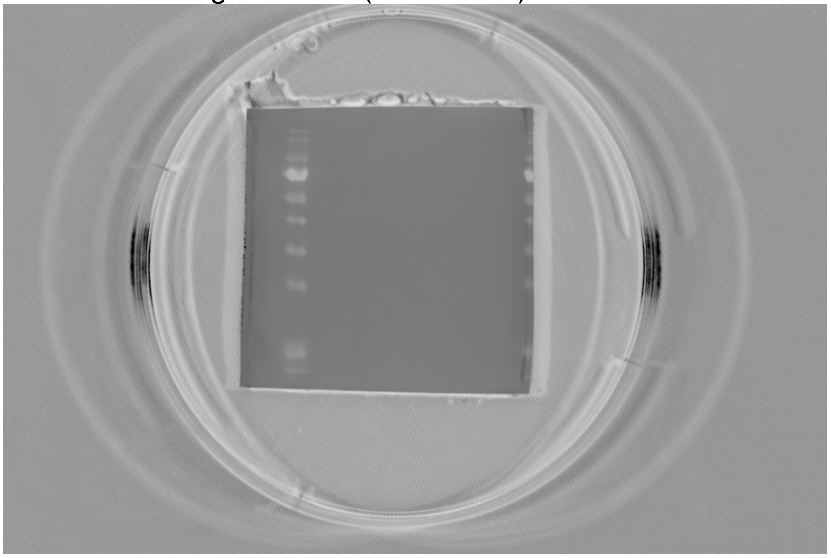

IrvR Autocleavage (MW marker)

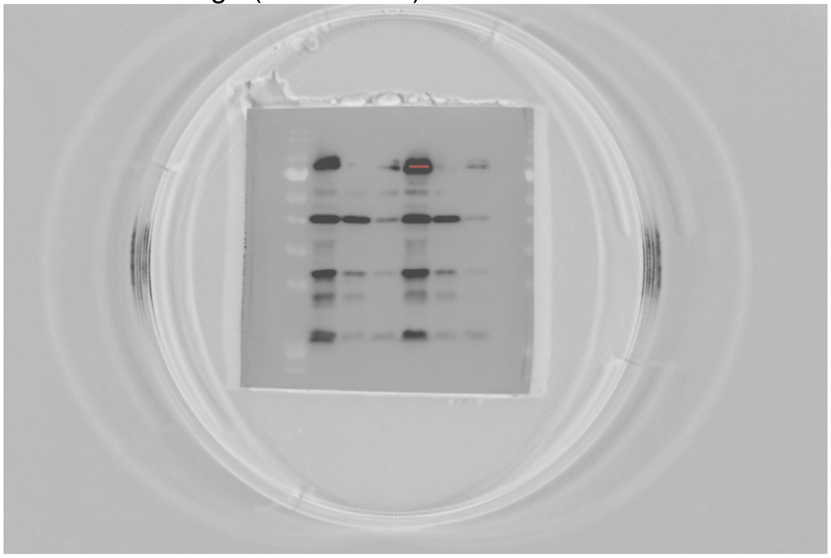

IrvR Autocleavage (western + MW marker; Lanes #5-7)

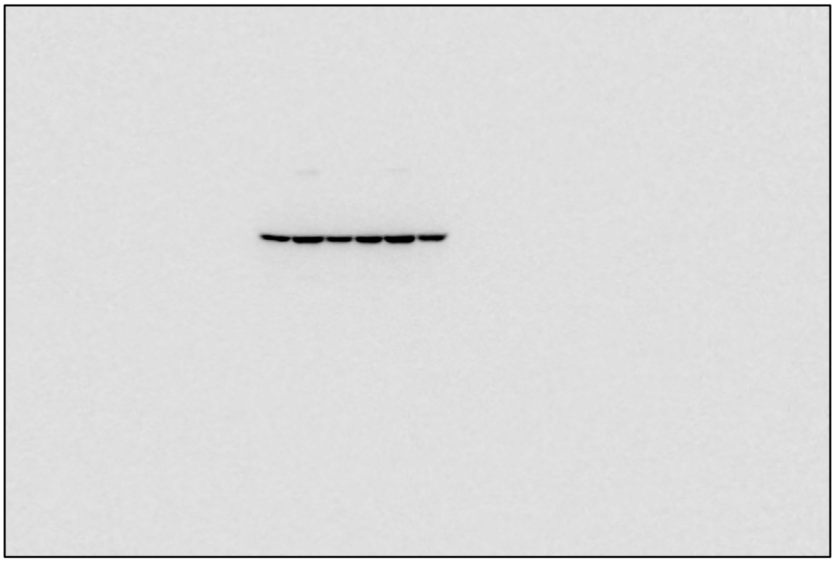
Loading control western (Lanes #5-7)
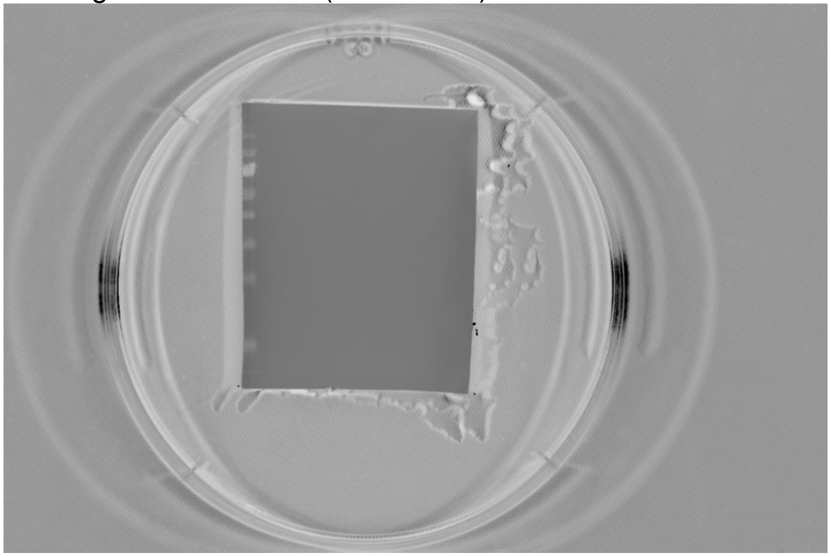
Loading control (MW marker)
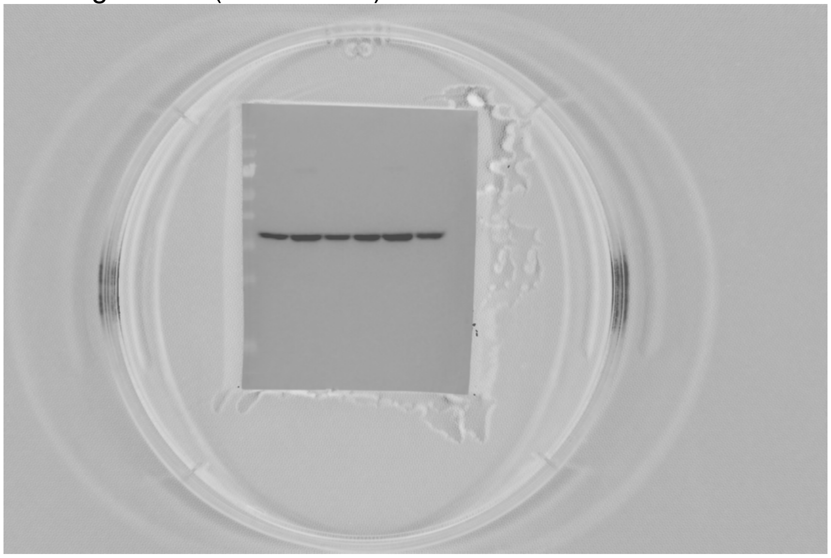
Loading control (western + MW marker overlay; Lanes #5-7)

Supplementary Figure 1A

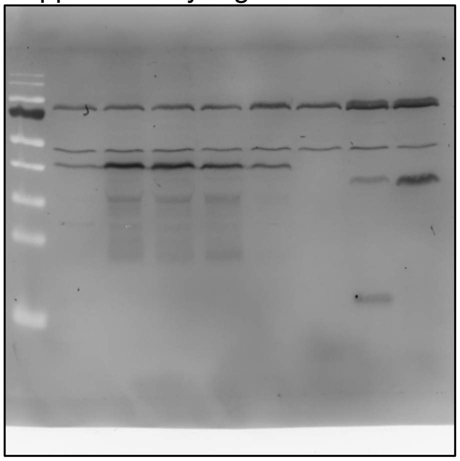

IrvR Autocleavage (Lanes #7-8)

Supplementary Figure 2A

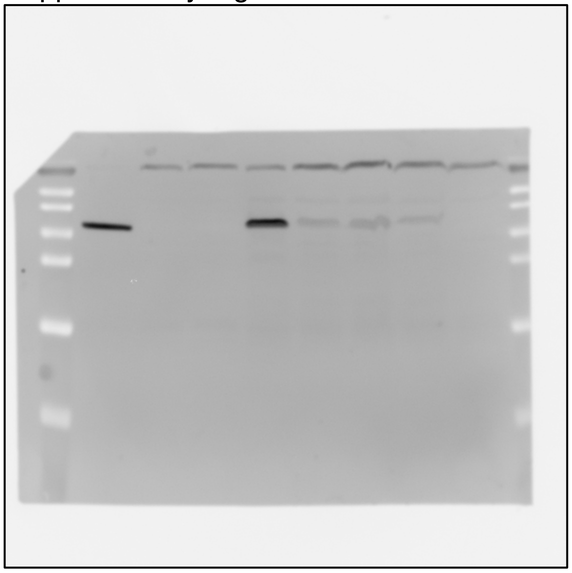

IrvR Autocleavage (Lanes #5-8)

Supplementary Figure 3C

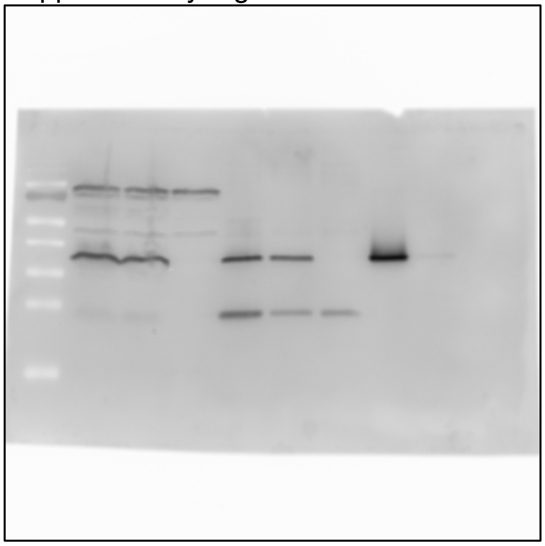

FLAG input, IP, and co-IP

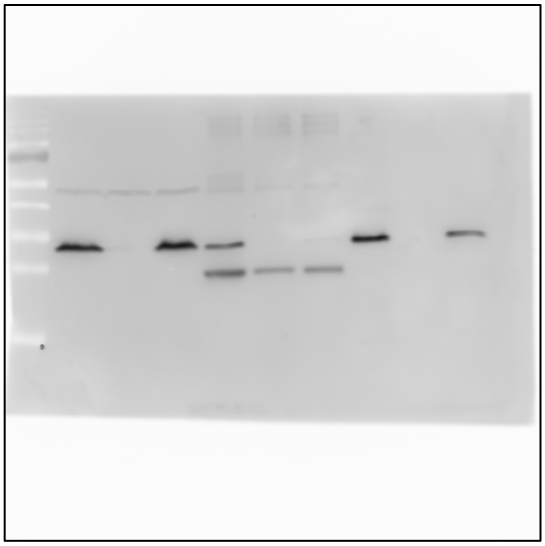

HA input, IP, and co-IP

Supplementary Figure 3D

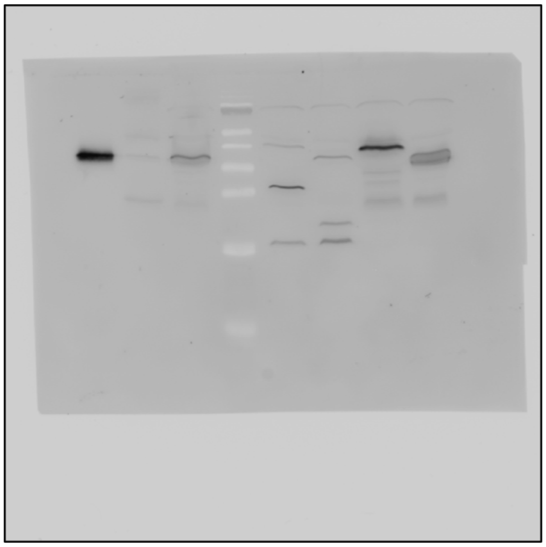

IrvR Autocleavage (Lanes #5-8)

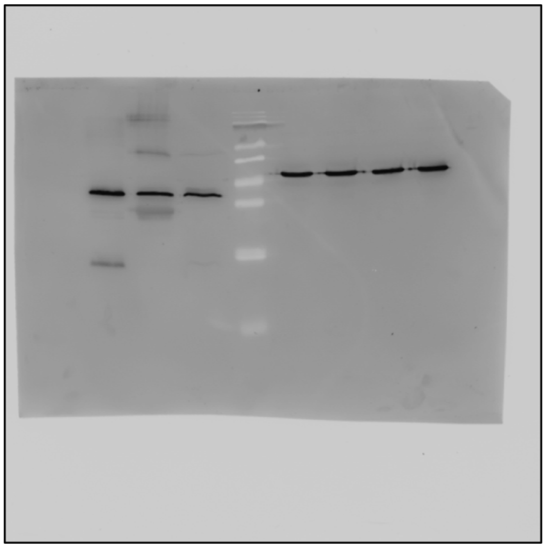

Loading control (Lanes #5-8)

Supplementary Figure 3E

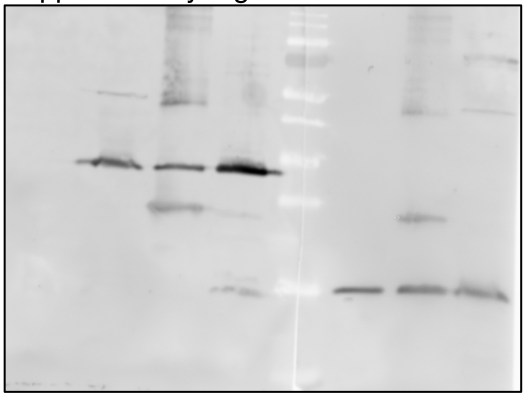

FLAG input, IP, and co-IP (Lanes #5-7)

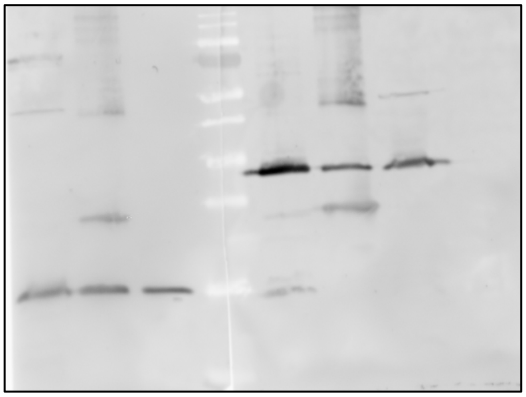

HA input, IP, and co-IP (Lanes #5-7)

Supplementary Figure 4G

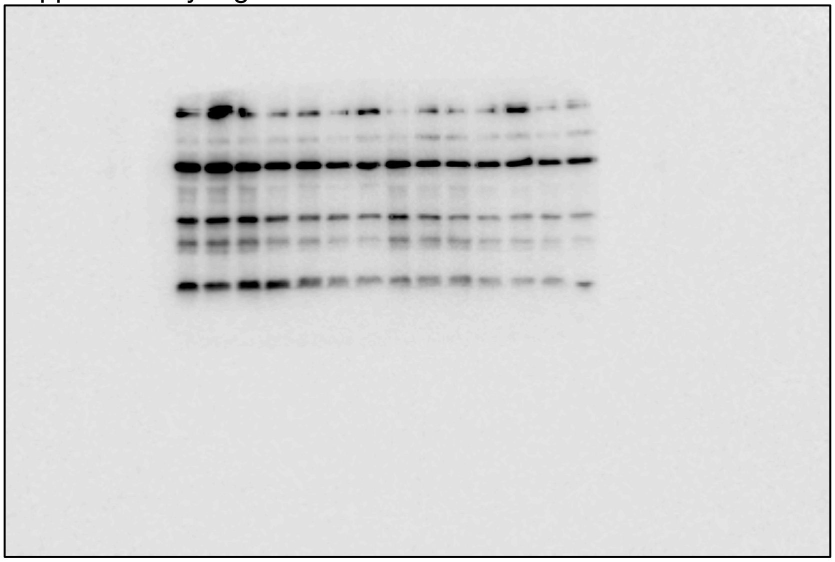

IrvR stability assay western (Lanes #3-14)

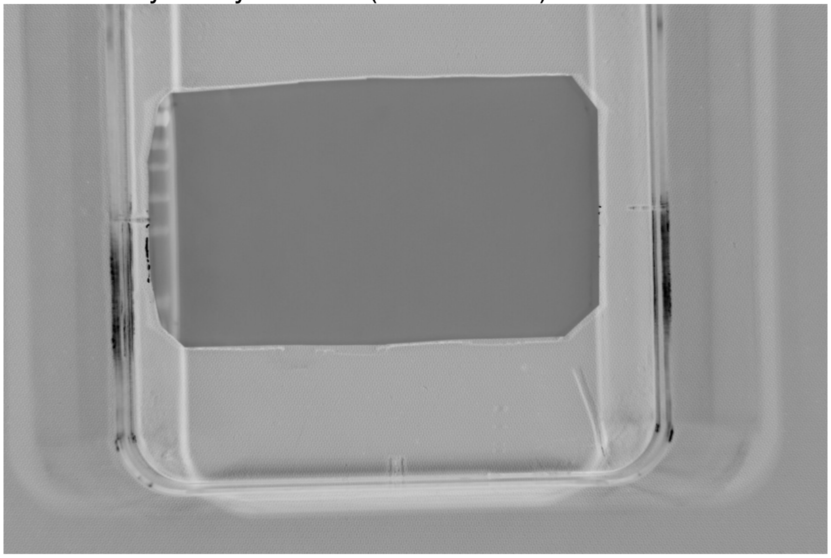

IrvR stability assay (MW marker)

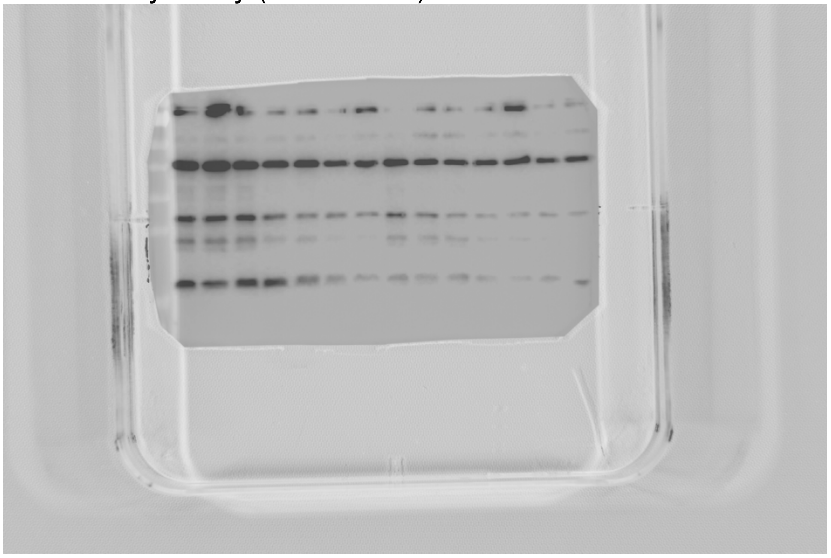

IrvR stability assay (western + MW marker overlay; Lanes #3-14)
